# Dual-Emitting Cyclometalated Platinum Compounds with Isocyanide Ligands

**DOI:** 10.1021/acsomega.5c07648

**Published:** 2025-10-13

**Authors:** Craig M. Anderson, Matthew W. Greenberg, Christopher N. LaFratta, Ryan H. Lum, Everest I. Oppenheimer, Teeka S. Duplessis, Kris M. Tulloch, Joseph M. Tanski

**Affiliations:** † Department of Chemistry & Biochemistry, 1263 Bard College, 30 Campus Road, Annandale-On-Hudson, New York, New York 12504, United States; ‡ Department of Chemistry, Vassar College, Poughkeepsie, New York 12604, United States

## Abstract

Cyclometalated platinum­(II) compounds with both C^N chelating iminic ligands and isocyanide ligands were synthesized. Two sets of compounds from the reaction of two separate HC^N ligands (derived from thiophene or benzene) with [Pt_2_Me_4_(μ-SMe_2_)_2_] were obtained, resulting in square planar compounds with an anionic C^N ligand, a methyl ligand, and a dimethyl sulfide (dms) ligand completing the coordination sphere. Subsequently, the dms ligand was easily substituted by several isocyanide ligands (2-naphthyl, adamantyl, 2,6-dimethylphenyl, *p*-toluenesulfonylmethyl). The compounds were characterized by multinuclear NMR spectroscopy, IR spectroscopy, and single-crystal X-ray diffraction (SCXRD). Their photophysical properties were explored using UV/vis, emission, and transient absorption (TA) spectroscopy. The emission spectra for the thiophene-derived compounds showed well-defined dual emission peaks, while the benzene-derived compounds’ bands appeared less resolved. DFT and TDDFT calculations were performed, and results were compared to the observed spectroscopic properties of the newly synthesized complexes.

## Graphical abstract

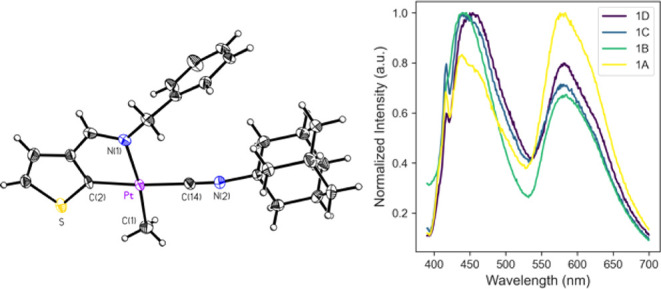

## Introduction

Transition metal compounds with cyclometalated ligands have attracted a great deal of attention as a result of their broad range of applications in both chemistry and material science.^1–3^ Cyclometalated compounds have been studied and found to be of great value for decades.^4–8^ The applications of these complexes include sensors, catalysts, potential light harvesting devices, and light-emitting diodes (LEDs).^9,10^ In fact, applications of luminescent compounds and materials cover a very wide variety of areas including information storage and anticounterfeiting.^11,12^ Among emissive compounds, dual-emitting compounds, compounds that emit photons of two different frequencies from two different states, are sought after for a variety of reasons, including ratiometric sensors and improving the turnability of devices.^13–16^ Moreover, the structure of a luminescent complex also appears to be important and germane to understanding its photophysical properties, as this may affect packing and π-π interactions in the solid state.^17^ Additionally, some compounds can exhibit mechanochromic behavior.^18,19^ Isocyanides are an interesting class of ligands that allow additional steric and electronic properties to be explored when incorporated into the coordination sphere of a metal compound.^20–22^ Furthermore, thiophene, which is well-known for its exceptional optical properties also allows further tuning of the photophysical properties when included in the cyclometalation framework architecture.^23–26^ This study reports the synthesis, characterization, and photophysical properties, including transient absorption spectroscopy of a series of dual-emitting thiophene-derived, cyclometalated platinum compounds with isocyanide auxiliary ligands.

## Results and Discussion

### Synthesis and Characterization

Eight new cyclometalated compounds (Scheme 1) with isocyanide ligands, including four based on the thiophene-derived imine ligand (**LA**) and four based on the benzene derived imine ligand (**LB**) were synthesized. The compounds were characterized by proton NMR, carbon NMR, IR, absorbance, and emission spectroscopies. Several of the compounds were characterized by single crystal X-ray diffraction (SCXRD) measurements to confirm their structure in the solid state. Scheme 1 illustrates the syntheses of the compounds where the isocyanide ligand easily substitutes for the labile dimethyl sulfide (dms) ligand. The proton NMR spectra were invaluable in the characterization of the compounds. For example, compound **1C** exhibited several diagnostic resonances and shifts compared to the starting materials, indicating its formation (Table 1). The spectrum of **1C** has a resonance at 1.05 for the Pt–Me group with a Pt coupling constant of 84 Hz, which is typical for platinum­(II) compounds.^27,28^ This methyl resonance replaced that of the precursor platinum compound **1** (Scheme 1) of 1.10 ppm. Additionally, the imine and the two methylene resonances were used to conclusively identify the product. Table 1 gives the pertinent NMR data for all eight compounds. As an additional aid in the characterization of the phenyl-derived cyclometalated compounds there exists a three-bond coupling to the cyclometalated ortho position proton. Infrared spectroscopy was used to examine the isocyanide ligand N≡C stretch (Table 2). Interestingly, the stretch frequency increases upon coordination to the platinum suggesting negligible π-backbonding in these complexes. This is corroborated by our DFT studies where either an increase or small (<20 cm^–1^) decrease in the calculated N≡C stretching frequency is observed for different complexes (Table S5). As mentioned, several compounds had their structures determined crystallographically by SCXRD. These included five of the eight compounds, **1A**, **1C**, **2B**, **2C**, and **2D** (Figures 1–5. respectively), thus at least two examples for each type of cyclometalated ligand and at least one with each isocyanide ligand. The bond lengths are normal for analogous platinum­(II) cyclometalated compounds.^24^ The angle of the Pt atom and the isocyanide triple bond is nearly linear.^21,29^ In all cases the methyl ligand is trans to the imine while the isocyanide is trans to the cyclometalated carbon atom.

**Scheme 1:**
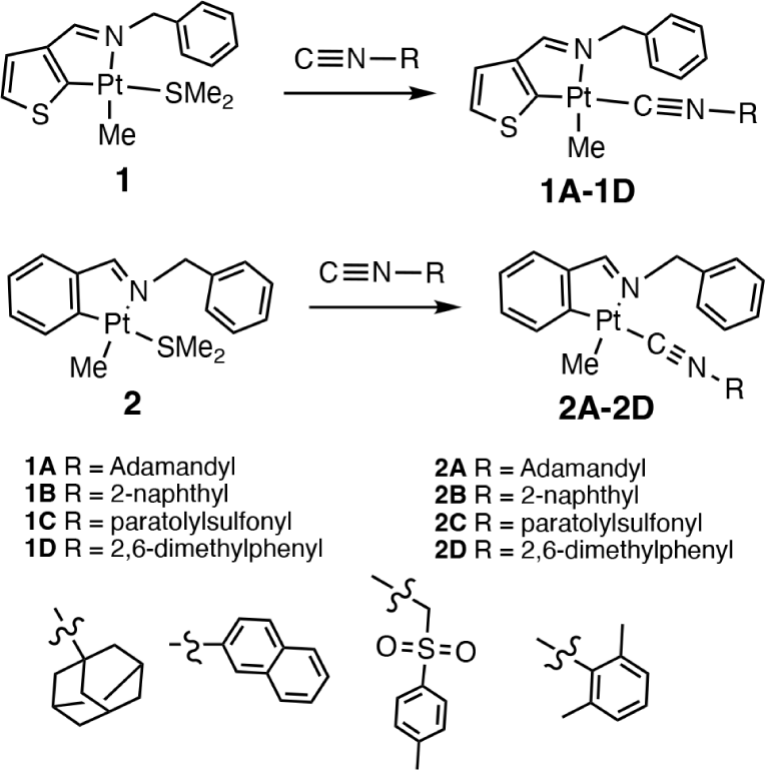
Synthesis of Platinum Compounds

**Table 1:** Selected ^1^H-NMR Data for Pt­(II) Compounds^a^

	CH=N ^3^ *J*(Pt–H)	Ph–CH_2_^3^ *J*(Pt–H)	CH_2_	CH_3_	Pt-CH_3_^2^ *J*(Pt–H)
**1A**	8.37, 51	5.12, 14	N/A	N/A	1.15, 84
**1B**	8.50, 51	5.21, 15	N/A	N/A	1.32, 86
**1C**	8.45, 53	5.05, 15	4.24	2.48	1.05, 84
**1D**	8.41, 52	5.17, 16	N/A	N/A	1.20, 83
**2A**	8.54, 54	5.23, 16	N/A	N/A	1.21, 83
**2B**	8.67, 54	5.32, 14	N/A	N/A	1.22, 87
**2C**	8.62, 54	5.17, 15	4.27	2.47	0.93, 86
**2D**	8.58, 53	5.29, 14	N/A	N/A	1.20, 85

a Columns from left to right: complex name; δ N=CH, ^3^ *J*(Pt–H); δ CH_2_, ^3^ *J*(H–H); δ S–CH_2_; δ para-CH_3_; δ PtMe, ^2^ *J*(Pt–H). δ values in ppm and *J* values in Hz.

**Table 2:** N≡C Triple Bond IR Stretching Frequency for Pt­(II) Compounds

**Free ligand N≡C (cm** ^**–1**^)	**N≡C (cm** ^**–1**^)		**Δ (cm** ^**–1**^)	
**1-Adamantyl isocyanide** 2121	**1A** 2168	**2A** 2143	**1A** 47	**2A** 22
**2-Naphthyl isocyanide** 2122	**1B** 2144	**2B** 2136	**1B** 22	**2B** 14
***p*-Toluenesulfonylmethyl Isocyanide** 2153	**1C** 2161	**2C** 2155	**1C** 8	**2C** 2
**2,6 Dimethylphenyl Isocyanide** 2120	**1D** 2143	**2D** 2130	**1D** 23	**2D** 10

**Figure 1:**
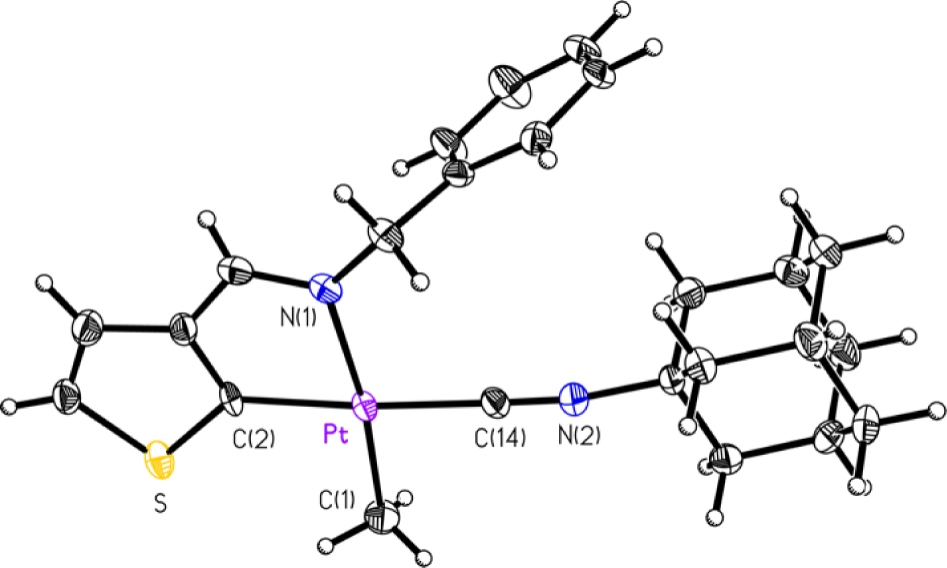
ORTEP of compound **1A** (50% probability thermal ellipsoids). Selected bond lengths (Å) and angles (°): Pt–C14: 1.954(3); Pt–C1: 2.050(4); Pt–C2: 2.021(3); Pt–N1: 2.132(3); C14–Pt–C2: 175.30(14); C14–Pt–C1: 90.04(15); C2–Pt–C1: 94.38(14); C14–Pt–N1: 97.32(13); C2–Pt–N1: 78.33(12); C1–Pt–N1: 172.23(12).

**Figure 2:**
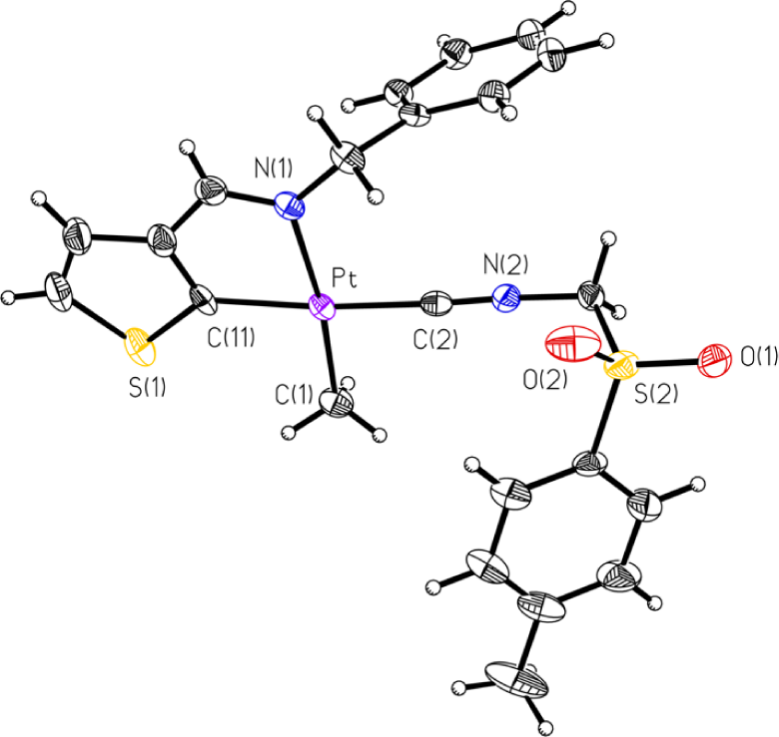
ORTEP of Compound **1C** (50% probability thermal ellipsoids). Selected bond lengths (Å) and angles (°): Pt–C2: 1.924(3); Pt–C11: 2.017(3); Pt–C1: 2.063(3); Pt–N1: 2.123(2); C2–Pt–C11: 174.44(12); C2–Pt–C1: 90.71(12); C11–Pt–C1: 94.60(12); C2–Pt–N1: 96.62(10); C11–Pt–N1: 78.12(11); C1–Pt–N1: 172.45(11).

**Figure 3:**
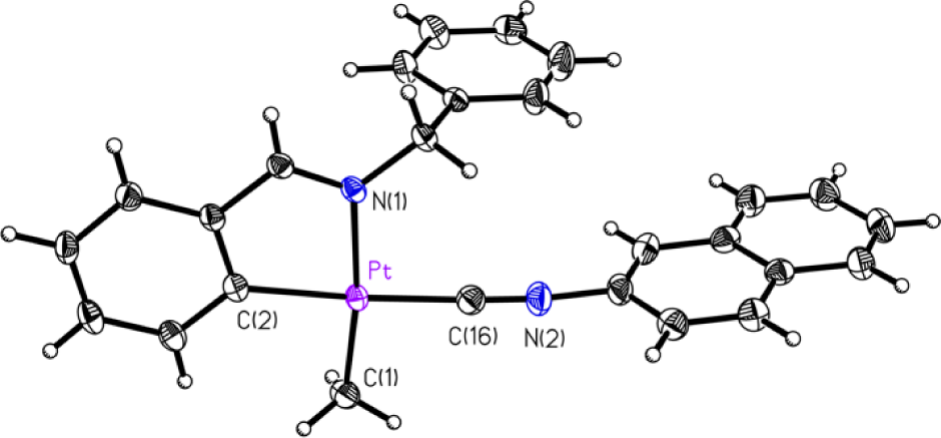
ORTEP of compound **2B** (50% probability thermal ellipsoids). Selected bond lengths (Å) and angles (°): Pt–C16: 1.944(2); Pt–C2: 2.048(2); Pt–C1: 2.061(2); Pt–N1: 2.1060(18); C16–Pt–C2: 174.61(9); C16–Pt–C1: 91.86(10); C2–Pt–C1: 93.36(9); C16–Pt–N1: 95.25(8); C2–Pt–N1: 79.59(8); C1–Pt–N1: 172.47(8).

**Figure 4:**
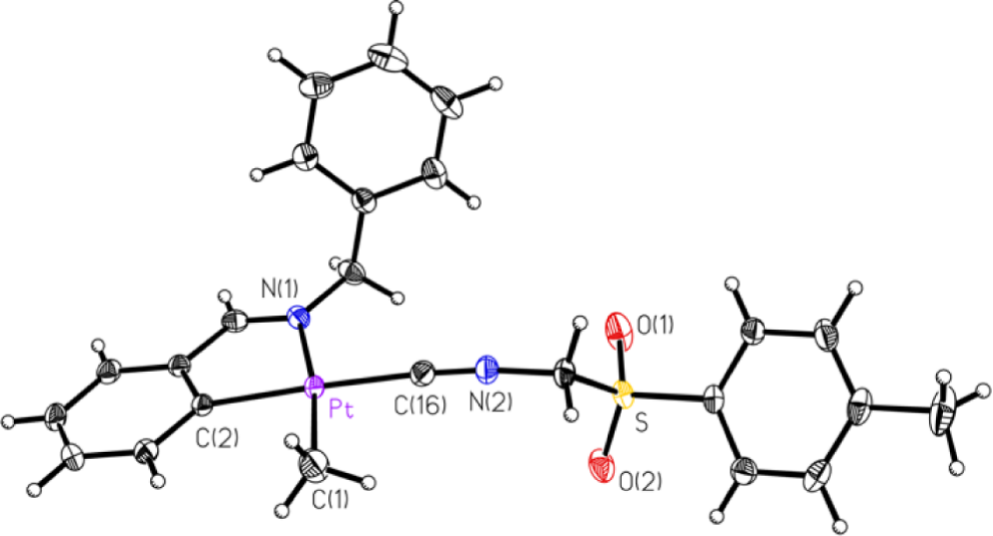
ORTEP of compound **2C** (50% probability thermal ellipsoids). Selected bond lengths (Å) and angles (°): Pt–C16: 1.948(2); Pt–C2: 2.046(2); Pt–C1: 2.057(2); Pt–N1: 2.1136(18); C16–Pt–C2: 177.97(8); C16–Pt–C1: 87.03(10); C2–Pt–C1: 91.86(9); C16–Pt–N1: 101.35(8); C2–Pt–N1: 79.74(7); C1–Pt–N1: 171.57(8).

**Figure 5:**
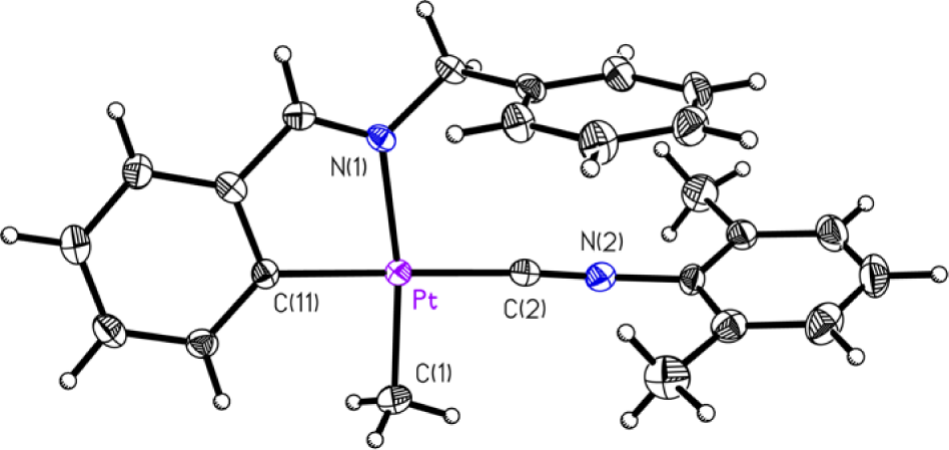
ORTEP of compound **2D** (50% probability thermal ellipsoids). Selected bond lengths (Å) and angles (°): Pt–C2: 1.951(3); Pt–C11: 2.041(3); Pt–C1: 2.051(3); Pt–N1: 2.116(2); C2–Pt–C11: 173.91(11); C2–Pt–C1: 89.46(12); C11–Pt–C1: 91.92(12); C2–Pt–N1: 99.24(10); C11–Pt–N1: 79.59(10); C1–Pt–N1: 171.14(11).

The absorbance (UV/vis) and emission spectra of the platinum compounds were measured in dichloromethane (DCM) solution. The UV/vis spectra exhibited several bands in the UV region with the lowest energy band tailing into the visible region (Figure 6). The lowest energy bands have extinction coefficients with a magnitude of around 10^3^ and are thus tentatively assigned to a MLCT transition (see TDDFT results below).^30^ The emission spectra of the thiophene-derived compounds had some interesting features, the most prominent being the presence of two well-defined emission bands (Figure 7). These emission bands are tentatively assigned to a fluorescence band and a phosphorescence band.^31^ The benzene-derived compounds also showed dual emission but with less-defined peaks (Figure S63).

**Figure 6:**
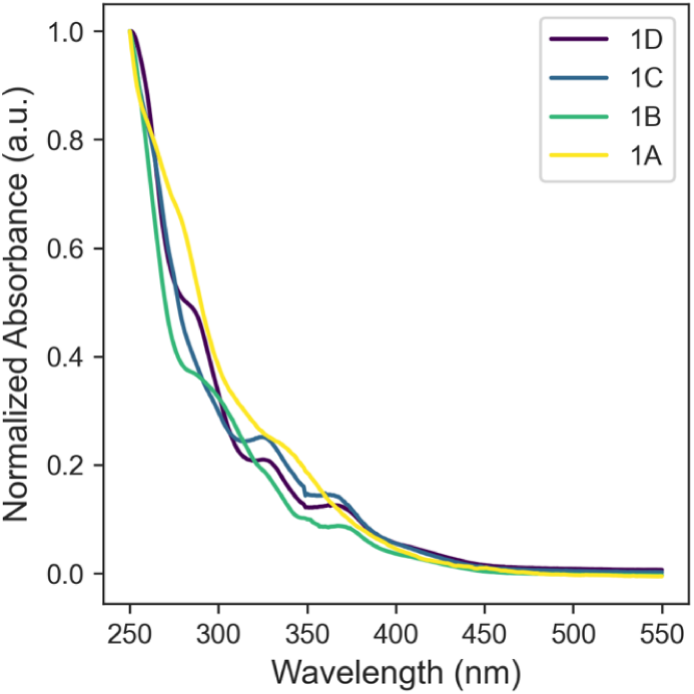
UV/vis spectra (in DCM) of compounds **1A**–**1D**.

**Figure 7:**
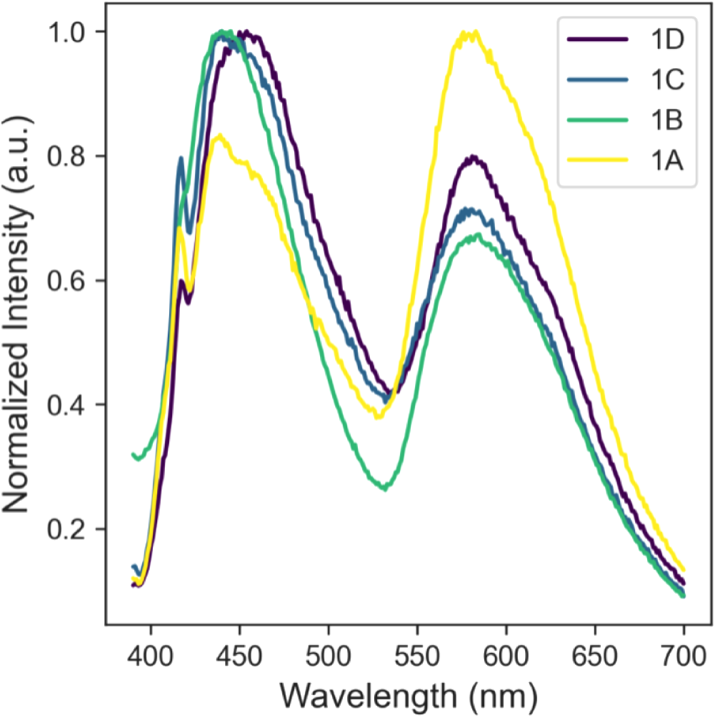
Steady state emission spectra (in degassed DCM) of **1A**–**1D** excited at 370 nm.

Several similar platinum­(II) isocyanide complexes with cyclometalated phenylpyridine have been reported along with their emission spectra in the solid state or in glassy media. These differ from our emission spectra as our samples’ emission in room temperature solution clearly contain two distinct emission bands.^22^

The excited state lifetimes were measured for the compounds by time correlated single photon counting (TCSPC). The higher energy band had a short lifetime of several nanoseconds, as would be expected of a ligand-centered fluorescence.^32^ The lower energy band had short lifetimes for the phenyl-derived compounds of several nanoseconds, while the thiophene-derived compounds approached hundreds of nanoseconds (Table S3). The extremely low values were not expected for compounds with large Stokes shifts of around a couple hundred nm for the phenyl derivatives. Unfortunately, the quantum yields were quite modest, at around 1% (Table S2), for all eight newly reported compounds.

Femtosecond transient absorption spectroscopy (fs-TA) was performed to provide insight into the excited state formation and decay dynamics in these compounds. A representative set of spectra for the fs-TA of compound **2B** for time delays of 100 fs–7 ns is shown in Figure 8. The data for all compounds measured shows a broad panchromatic positive absorption band following excitation in the UV–vis, that are similar to spectra reported for similar organometallic Pt­(II) complexes that have been assigned to overlapping absorption from S1–SN and T1–TN states.^33^ The representative spectra of **2B** show a higher energy absorption band around 435 nm and a lower band centered around 650 nm, with the higher energy band showing faster decay during the 7 ns time window then the long-lived lower energy band. Global fitting was performed for all compounds and are well fit by a model with three time constants. These consisted of: 1) a long-lived (>7 ns) “infinite,” lifetime associated with long-lived triplet relaxation; and 2) two intermediate lifetimes (∼10 ps and ∼500 ps). Very fast (<1 ps) excited state dynamics associated with solvent excitation (Figure S73) are seen at higher energies. The infinite lifetime represents the long-lived triplet excited state, and the decay associated spectrum (Figure 9) shows this is associated with the broad absorption at lower energies around 650 nm that persists after 7 ns. The intermediate lifetime can be assigned to decay processes involving the singlet excited state manifold that depopulate to the long-lived triplet state at long times and have absorption centered at higher energies, and are consistent with ultrafast ISC rates of similar complexes with a heavy Pt­(II) metal center.^34,35^ Other representative spectra, and details of global fitting and decay associated spectra are shown in Figures S65–S72.

**Figure 8:**
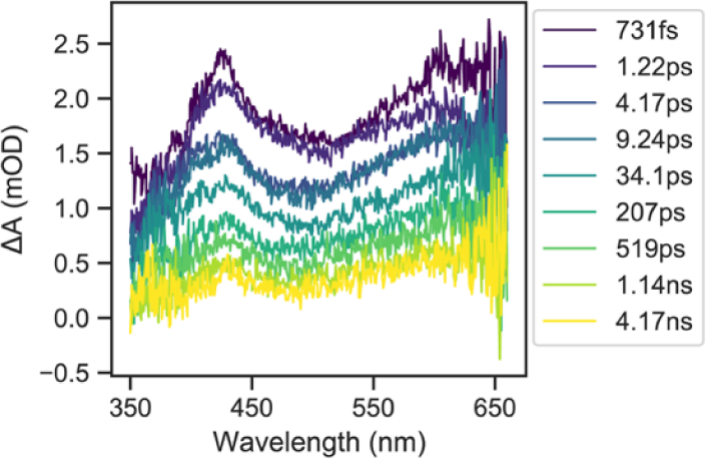
A representative set of fs-TA spectra (in degassed DCM) for compound **2B** for time delays of 100 fs–7 ns.

**Figure 9:**
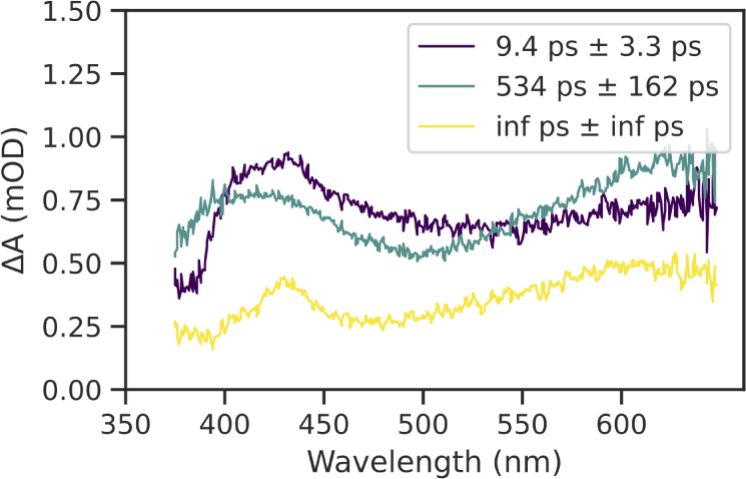
Decay-associated difference spectra (DADS) (in degassed DCM) from three-component global fitting of **2B** transient absorption surface.

The two values for ISC are consistent with certain cyclometalated platinum compounds, especially those with cyclometalated thiophene rings, as ISC can occur from the LC (π–π*) and the MLCT states.^36^ Thiophenes also are known to slow down ISC making fluorescence competitive in these dual-emitting compounds.^36^ Alternatively, thermally activated delayed fluorescence (TADF) could explain three lifetimes as there would be both an ISC and thermally accessible reverse intersystem crossing (RISC) process repopulating the single excited state.^37^ This should be accompanied by a temperature dependency of the ratio of fluorescence intensity to phosphorescence intensity in the steady state emission spectra, favoring phosphorescence as the temperature decreases. This was observed for spectra recorded at 77 K versus room temperature with increased phosphorescence intensity relative to fluorescence being observed (Figures S74 and S75). However, this behavior could reflect rigidification effects of the frozen glassy matrix on the photoluminescence.^8^ Furthermore, this second possibility of TADF appears less likely than the two ps lifetimes representing ISC from distinct states as has been previously assigned, given the TDDFT calculations (*vide infra*) indicate an energy gap between triplet and singlet that is too large for thermally accessible RISC. In total, the ultrafast decay dynamics are consistent with the observed dual emission, showing a singlet excited state that can facilitate fluorescence to the ground state.

## TDDFT and DFT Calculations

Density Functional Theory (DFT) and Time Dependent Density Functional Theory (TDDFT) calculations were performed to investigate the electronic spectra for all eight Pt­(II) isocyanide complexes. The DFT calculated frontier molecular orbitals for compound **1A** are shown in Figure 10 and other frontier and near frontier molecular orbitals for all compounds are shown in Figure S76. The occupied HOMO and near-HOMO orbitals for the complexes show clear Pt 5d orbital character with other electron density seen on the π system of the metalated ligand and isocyanide-N p orbital, while the LUMO and near LUMO orbitals are predominantly located in the π* orbitals of the ligands. Electronic transitions involving the frontier molecular orbitals and near frontier molecular orbitals of these complexes would be expected to be of a symmetry allowed mixed MLCT/ligand π-π* character.

**Figure 10:**
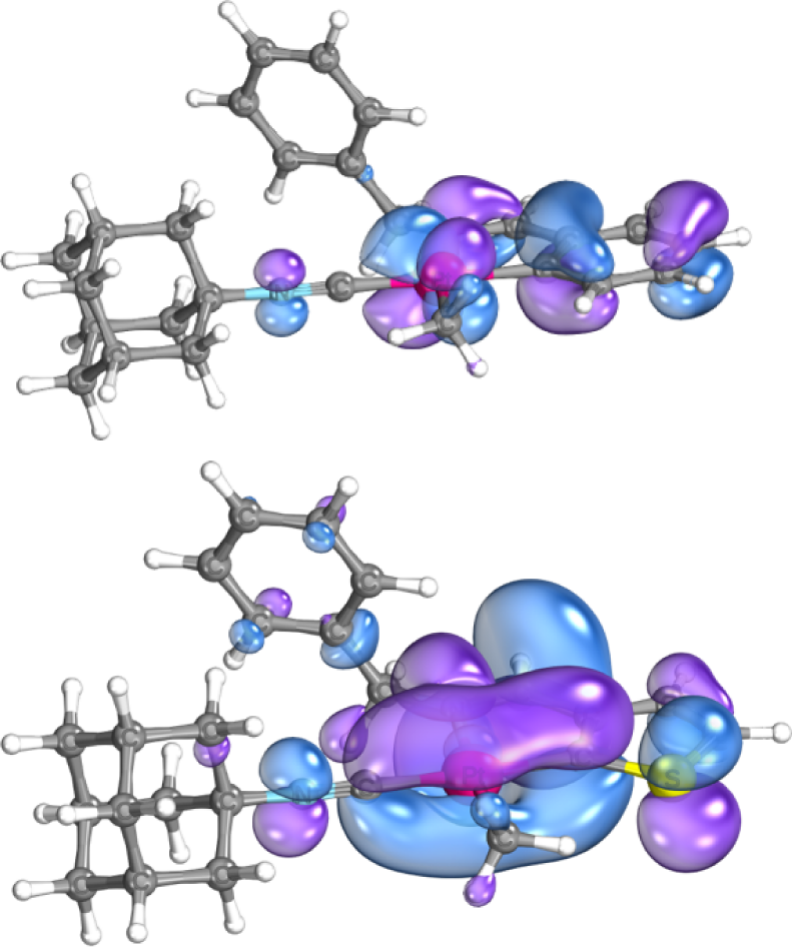
DFT calculated HOMO and LUMO of **1A** plotted at the 80% isosurface threshold using IboView.

As summarized in Table S4 for each of the complexes, UV/vis absorption peaks with extinction coefficients (10^3^) consistent with MLCT character are observed. For each complex, these absorbance peaks are located at ∼325 nm and ∼375 nm. TDDFT calculations are consistent with the experimental data, with high oscillator strength transitions seen around these energies, as can be seen in the simulated spectrum plotted alongside the experimental UV/vis for one of the complexes shown in Figure 11. All vertical excitations are shown in Figures S77 and S78. For the first three singlet transitions above 300 nm, that are in good agreement with the experimentally observed absorbance peaks at these wavelengths, representative Natural Transition Orbitals^38^ are shown and orbital contributions to each excitation are summarized in Figures S79–S84. The first excitation is the HOMO/LUMO transition at around ∼375 nm, while the next two higher energy transitions involve lower energy near frontier orbitals around the HOMO to the LUMO orbital. In each case, we assign these excitations as having MLCT/ligand π–π* character.

**Figure 11:**
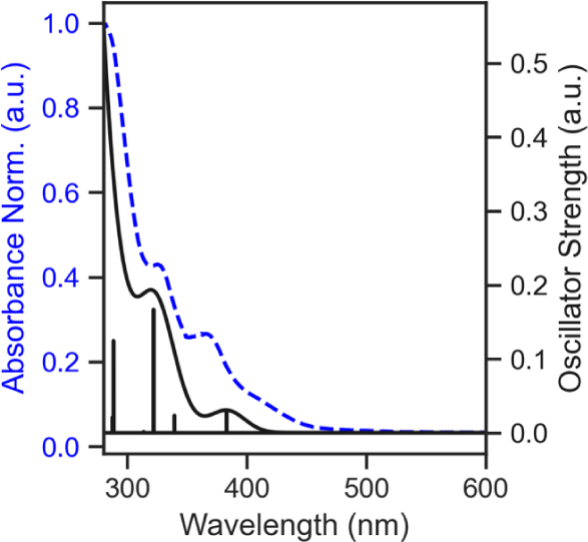
Normalized experimental absorbance and TDDFT simulated spectrum of **1D** from 280 to 600 nm. Calculated singlet vertical excitations are broadened with a 30 nm line width for generating the simulated spectrum.

Complexes were optimized in the ground singlet state, the first excited singlet state, and lowest energy triplet state to compare these energies to the observed dual emissive behavior of the compounds. The vibrational calculations of ground state structures are consistent with the experimental shift to higher frequency for the isocyanide N≡C stretch upon coordination to the metal. This is corroborated by our DFT studies which show either an increase or only a small (<20 cm^–1^) decrease in the calculated N≡C stretching frequency relative to the free ligands (), indicating negligible π-backbonding to the isocyanide in these complexes. Bond lengths obtained for the gas phase optimized geometry are in good agreement with the results from solid state SCXRD (Table S6). Comparison of the energy difference between these optimized S1–S0, and T1–S0 states (Tables S7–S8) show a good match with the observed energies of the emission bands we assign as phosphorescence and fluorescence. Previous studies of cyclometalated Pt­(II) compounds have also shown a similar correspondence between DFT calculated 0–0 transition energies and fluorescence and phosphorescence energies in dual emitting compounds.^39^ Additionally, excited state dynamics simulations of fluorescence and phosphorescence spectra for **1A** are in good agreement with the two bands in the experimentally obtained spectrum (Figures S85 and S86).

## Concluding Remarks

A series of thiophene-derived cyclometalated compounds were synthesized and characterized, and computationally modeled. These compounds exhibited dual emission in the visible region. Albeit their quantum yields were quite modest, these compounds may contribute to the understanding of dual-emissive compounds and aid in the development of devices that utilize ratiometric properties.

## Experimental Section

### General

Solvents and reagents were purchased from Sigma-Aldrich unless otherwise noted. K_2_PtCl_4_ was purchased from J and J Materials (NJ). NMR spectra were recorded at Bard College using a Varian NMR-400 MHz spectrometer (^1^H, 400 MHz; ^13^C, 100.6 MHz). Shifts are given in ppm and coupling constant *J* values are given in Hz. Abbreviations used: s = singlet; d = doublet; t = triplet; m = multiplet.

### Computational Details

TDDFT and DFT calculations were performed using Orca *ab initio* quantum chemistry program version 5.0.4.^40,41^ All calculations were performed using the B3LYP functional.^42,43^ The ZORA^44^ scalar relativistic approximation was used with ZORA-def2-TZVP^45^ basis sets for S, N, C, H, and O atoms and the SARC-ZORA-TZVP^46^ basis set was used for Pt, and SARC/J decontracted def2/J^47^ and SARC^48^ auxiliary basis sets. Geometry optimizations were carried out in the gas phase and confirmed to be an energetic minimum by harmonic vibrational analysis. UV–vis simulation TDDFT calculations of the singlet excitations were performed without the use of the Tamm–Dancoff approximation using 30 singlet excitations and a conductor-like polarizable continuum model (CPCM) for solvation effects.^49^ The T_1_ and S_1_ state calculations are the DFT geometry optimized lowest energy triplet and TDDFT geometry optimized lowest energy excited singlet, respectively. Excited state dynamics module calculations for phosphorescence and fluorescence spectra^50,51^ simulation were performed using the vertical gradient approximation for excited state Hessians and CPCM. For phosphorescence calculations, RI-SOMF­(1X) was employed to accelerate the SOC integrals.^52^

### Photophysical Measurements

Steady-state emission spectra were recorded using a PTI QM-40 instrument with a PMT detector, which is sensitive up to 850 nm. In these experiments, the concentration of the platinum complexes ranged from 67 to 75 μM in degassed solvents. The fluorimeter emission spectrum was corrected using a method described in the literature which uses four standard fluorophores to calibrate the response of the instrument.^53^ The slits were set at 2.5 nm bandpass for all solution measurements. The luminescence lifetimes of the complexes were measured by time-correlated single-photon counting (TCSPC) following excitation with a 365, 405, or 450 nm LED. For TCSPC measurements the slits were adjusted such that <3% of the LED flashes resulted in a detection event ensuring such events are single photons. Quantum yield (QY) measurements were taken using a PTI QM400 (Horiba) equipped with a petite integrating sphere and a 920C cooled PMT detector. During quantum yield measurements, slit sizes were adjusted and a neutral density filter with an optical density of 1.0 was used to keep the signal in the linear range of the detector (<1 Mcps). Excitation and emission slits were typically set to ∼1 nm bandpass. The solvent, DCM, was used as a reference. The integrating sphere was corrected by scaling the solvent emission curve by the ratio of the integrated excitation peaks for the sample and the blank. The accuracy of the integrating sphere was checked using [Ru­(bpy)_3_]­Cl_2_ in water as a standard, with known quantum yield of 0.028.^54–56^ Its quantum yield was measured five times and determined to be 0.035 ± 0.008. Femtosecond Transient Absorption (fs-TA) spectroscopy was performed at Brookhaven National Laboratory using a Helios Fire (Ultrafast Systems) spectrometer that used a Spitfire SpectraPhysics regenerative amplifier and two TOPAS OPAs operating at 1 kHz repetition rate. fs-TA data reduction and analysis was performed using Surface Xplorer 4.3.0 software (Ultrafast Systems). Raw data were background subtracted and chirp corrected. Singular value decomposition was used to determine the number of principal components. Multiexponential global analysis fitting was then performed to extract decay associated difference spectra and their corresponding lifetimes.

### X-ray Diffraction

**1A**, **2B**, **2C**, and **2D** were crystallized by slow diffusion of pentane into an acetone solution, **1C** was crystallized by slow diffusion of pentane into a chloroform solution. X-ray diffraction data were collected on a Bruker APEX 2 CCD platform diffractometer (Mo Kα (*l* = 0.71073 Å)) at 125 K with crystals mounted in a nylon loop with Paratone-N cryo-protectant oil. The structures were solved using direct methods (SHELXT 2018/2)^57^ and standard difference map techniques, and were refined by full-matrix least-squares procedures on F2 (SHELXL 2017/1).^58^ All non-hydrogen atoms were refined anisotropically.

### Preparation of Compounds

See the Supporting Information for additional experimental details, including copies of NMR spectra, UV/vis spectra, and emission spectra. [Pt_2_Me_4_(μ-SMe_2_)_2_] was prepared according to the literature.^59^ **1** and **2** were synthesized according to the literature.^60,61^

#### [C_24_H_28_N_2_PtS], **1A**

**Compound 1** (0.0731 g, 154.70 μmol) and 1-adamantyl isocyanide (0.0251 g, 156 mmol) were dissolved in 10 mL of acetone and stirred at room temperature for 60 min. The solvent was then removed by rotary evaporator and the resulting orange powder was washed with cold pentane (3 × 1.5 mL) and cold ether (2 × 2 mL), and dried under vacuum. Yield: 0.094 g, 95%. ^1^H NMR (400 MHz, CDCl_3_): δ 1.15 (s, 3H, MePt, ^2^ *J*(Pt–H) = 85), 1.57 (d, 3H, CH_2_, ^2^ *J*(H–H) = 13), 1.64 (d, 3H, CH_2_, ^2^ *J*(H–H) = 13), 1.80 (br s, 6H, CH_2_), 2.05 (br s, 3H, CH), 5.12 (s, 3H, CH_2_, ^3^ *J*(Pt–H) = 14), 7.15–7.4 (aromatics), 8.37 (s, 1H, N=C, ^3^ *J*(Pt–H) = 51) ppm. ^13^C NMR (CDCl_3_): δ −6.83 (^1^ *J*(Pt–C) = 591 Hz), 28.9, 35.5, 42.9, 43.2, 125.3, 126.2, 127.3, 127.6, 128.6, 129.2, 168.8 ppm. Elemental analysis, % calculated for C_24_H_28_N_2_PtS: C, 50.43; H, 4.94; N, 4.90; found: C, 50.13; H, 5.43; N, 4.12.

#### [C_24_H_20_N_2_PtS], **1B**

**1** (0.0779 g, 165 μmol) and 2-naphthyl isocyanide (0.0254 g, 166 μmol) were dissolved in 10 mL of acetone and stirred at room temperature for 60 min. The solvent was removed by rotary evaporation, and the product was washed with cold pentane (3 × 1.5 mL) and cold ether (2 × 2 mL), and dried under vacuum. Yield: 0.093 g, 89.9%. ^1^H NMR (400 MHz, CDCl_3_): δ 1.32 (s, 3H, MePt, ^2^ *J*(Pt–H) = 86), 5.21 (s, 2H, CH_2_, ^3^ *J*(Pt–H) = 15), 7.8–7.1 (aromatics), 8.50 (s, 1H, N=C, ^3^ *J*(Pt–H) = 51) ppm. ^13^C NMR (CDCl_3_): δ, −26.0, 29.9, 123.2, 125.5, 126.8, 127.5, 127.7, 127.8, 128.0, 128.2, 128.9, 129.7, 130.0, 168.6 ppm. Elemental analysis, % calculated for C_24_H_20_N_2_PtS·1.45 H_2_O: C, 48.88; H, 3.91; N, 4.75; found: C, 49.23; H, 3.91; N, 4.33.

#### [C_22_H_22_N_2_O_2_PtS_2_], **1C**

**1** (0.0713 g, 151 μmol) and *p*-toluenesulfonylmethyl isocyanide (0.0295 g, 151 μmol) were dissolved in 10 mL of acetone and stirred at room temperature for 60 min. The solvent was removed by rotary evaporation, and the resulting orange powder was washed with cold pentane (3 × 1.5 mL) and cold ether (2 × 2 mL), and dried under vacuum. Yield: 0.097 g, 96%. ^1^H NMR (400 MHz, CDCl_3_): δ 1.05 (s, 3H, MePt, ^2^ *J*(Pt–H) = 83.4), 2.48 (s, 3H, CH_3_), 4.24 (s, 2H, CH_2_, ^3^ *J*(Pt–H) = 11), 5.06 (s, 2H, CH_2_, ^3^ *J*(H–H) = 15), 7.8–7.2 (aromatics), 8.45 (s, 1H, N=CH, ^3^ *J*(Pt–H) = 53) ppm. ^13^C NMR (CDCl_3_): δ 168.4, 147.1, 130.6, 129.5, 128.7, 127.9. 127.6, 125.4, 122.6, 65.5, 62.4, 21.9, −25.7 ppm. Elemental analysis, % calculated for C_22_H_22_N_2_O_2_PtS_2_ **·**1.25 H_2_O: C, 42.07; H, 3.93; N, 4.46; found: C, 42.80 H, 4.26 N, 3.67.

#### [C_22_H_22_N_2_PtS], **1D**

**1** (0.0758 g, 160 μmol) and 2,6 dimethylphenyl isocyanide (0.02104 g, 160 μmol) were dissolved in 10 mL of acetone and stirred at room temperature for 60 min. The solvent was removed by rotary evaporator, and the resulting orange powder was washed with cold pentane (3 × 1.5 mL) and cold ether (2 × 2 mL), and dried under vacuum (0.094 g, 93%). ^1^H NMR (400 MHz, CDCl_3_): δ 1.21 (s, 3H, MePt, ^2^ *J*(Pt–H) = 83), 2.21 (s, 6H, CH_3_), 5.17 (s, 3H, CH_2_, ^3^ *J*(Pt–H) = 16), 7.24–7.04 (aromatics), 8.41 (s, 1H, N=C, ^3^ *J*(Pt–H) = 52) ppm. Elemental analysis, % calculated for C_22_H_22_N_2_PtS: C, 48.79; H, 4.09; N, 5.17; found: C, 48.70; H, 4.72; N, 4.45.

#### [C_26_H_30_N_2_Pt], **2A**

**2** (0.0573 g, 123 μmol) and 1-adamantyl isocyanide (0.0198 g, 123 μmol) were dissolved in acetone and stirred at room temperature for 60 min. The solvent was removed by rotary evaporation and the resulting yellow powder was washed with cold pentane (3 × 1.5 mL) and cold ether (2 × 2 mL), and dried under vacuum. Further purification processes were needed and the product was then crystallized utilizing the vial in a jar technique. Yield: 0.064 g, 92%. ^1^H NMR (400 MHz, CDCl_3_): δ 1.21 (s, 3H, MePt, ^2^ *J*(Pt–H) = 83), 1.59 (d, 3H, CH_2_, ^2^ *J*(H–H) = 16), 1.66 (d, 3H, CH_2_, ^2^ *J*(H–H) = 16), 1.83 (br s, 6H, CH_2_), 2.06 (br s, 3H, CH), 5.23 (s, 2H, CH_2_, ^3^ *J*(Pt–H) = 14), 7.45–7.27 (Aromatics), 7.76 (d, 1H, ^2^ *J*(H–H) = 8, ^3^ *J*(Pt–H) = 49), 8.54 (s, 1H, N=C, ^3^ *J*(Pt–H) = 54) ppm. ^13^C NMR (CDCl_3_): δ −20.1, 28.9, 35.6, 43.1, 124.2, 127.4, 127.7, 128.8, 131.0, 132.9 (*J* = 93 Hz), 137.8, 150.3, 165.4 ppm. Elemental analysis, % calculated for C_26_H_30_N_2_Pt·2.25 H_2_O: C, 51.52; H, 5.74; N, 4.62. Found: C, 50.93; H, 5.13; N, 4.21.

#### [C_26_H_22_N_2_Pt], **2B**

**2** (0.0579 g, 120 μmol) and 2-naphthyl isocyanide (0.01846 g, 120 μmol) were dissolved in 10 mL of acetone and stirred at room temperature for 60 min. The solvent was removed by rotary evaporation, and the resulting yellow powder was washed with cold pentane (3 × 1.5 mL) and cold ether (2 × 2 mL) and dried under vacuum. Yield: 0.059 g, 88%. ^1^H NMR (400 MHz, CDCl_3_): δ 1.21 (s, 3H, MePt, ^2^ *J*(Pt–H) = 87), 5.32 (s, 2H, CH_2_, ^3^ *J*(Pt–H) = 14), 7.10–7.85 (Aromatics), 8.67 (s, 1H, N=CH, ^3^ *J*(Pt–H) = 54) ppm. ^13^C NMR (CDCl_3_): δ −19.7 (*J* = 678), 66.4, 123.1, 124.7, 125.4, 127.6, 127.7, 127.7, 127.9, 127.9, 128.2, 128.9, 129.7, 131.2, 132.9, 133.1, 137.7, 150.5, 165.9, 177.5 ppm. Elemental analysis, % calculated for C_26_H_22_N_2_Pt·1.1 H_2_O: C, 54.09; H, 4.22; N, 4.85; found: C, 54.59; H, 4.07; N, 4.27.

#### [C_24_H_24_N_2_O_2_PtS], **2C**

**2** (0.0579 g, 120 μmol) and p-toluenesulfonylmethyl isocyanide (0.02353 g, 120 μmol) were dissolved in 10 mL of acetone and stirred at room temperature for 60 min. The solvent was removed by rotary evaporation, and the resulting yellow powder was washed with cold pentane (3 × 1.5 mL) and cold ether (2 × 2 mL), and dried under vacuum. Yield: 0.070 g, 96. %. ^1^H NMR (400 MHz, CDCl_3_): δ 0.93 (s, 3H, MePt, ^2^ *J*(Pt–H) = 86), 2.47 (s, 3H, CH_3_), 4.27 (s, 2H, CH_2_, ^4^ *J*(Pt–H) = 13), 5.16 (s, 2H, CH_2_, ^3^ *J*(Pt–H) = 15), 7.12–7.81 (aromatics), 8.62 (s, 1H, N=CH, ^3^ *J*(Pt–H) = 54) ppm. ^13^C NMR (CDCl_3_): δ −19.3 ^1^ *J*(Pt–C) = 677), 22.0, 62.5, 66.1, 125.0, 127.7, 127.9, 128.0, 128.7, 129.5, 130.6, 131.2, 132.3, 133.0, 137.6, 147.0, 150.6, 155.1, 165.4, 177.4 (^1^ *J*(Pt–C) = 70). Elemental analysis, % calculated for C_24_H_24_N_2_O_2_PtS·1.15 H_2_O: C, 46.47; H, 4.27; N, 4.52. Found: C, 46.88; H, 3.95; N, 4.05.

#### [C_24_H_24_N_2_Pt], **2D**

**2** (0.0585 g, 121 μmol) and 2,6 dimethylphenyl isocyanide (0.01597 g, 121 μmol) were dissolved in 10 mL of acetone and stirred at room temperature for 60 min. The solvent was removed by rotary evaporator and the resulting yellow powder was washed with cold ether (2 × 2 mL) and cold pentane (3 × 1.5 mL), and dried under vacuum. Yield: 0.054 g, 83.%. ^1^H NMR (400 MHz, CDCl_3_): δ 1.20 (s, 3H, MePt, ^2^ *J*(Pt–H) = 85), 2.22 (s 6H, CH_3_), 5.29 (s, 2H, CH_2_, ^3^ *J*(Pt–H) = 14), 7.0–7.45 (aromatics), 7.81 (d, 1H, CH, ^2^ *J*(H–H) = 8, ^3^ *J*(Pt–H) = 49), 8.58 (s, 1H, N=C, ^3^ *J*(Pt–H) = 53) ppm. ^13^C NMR (CDCl_3_): δ −19.6 ^1^ *J* = 699 Hz), 18.7, 66.0, 124.5, 127.4, 127.7, 127.8, 128.0, 128.3, 128.6 (*J* = 92), 131.0 (*J* = 58), 132.8, 134.6, 137.2, 150.3, 156.0, 165.35, 177.2 (^2^ *J*(Pt–C) = 69). Elemental analysis, % calculated for C_24_H_24_N_2_Pt: C, 53.83; H, 4.52; N, 5.23; Found: C, 53.15; H, 4.41; N, 4.68.

## Supplementary Material













## Notes

NMR spectra, IR spectra, UV/vis and emission spectral data, and orbital plots (PDF)
CIF of **1A** (CIF)
CIF of **1C** (CIF)
CIF of **2B** (CIF)
CIF of **2C** (CIF)
CIF of **2D** (CIF)
