# Supplementary material for: Dual-Emitting Cyclometalated Platinum Compounds with Isocyanide Ligands
Source: ACS Omega. 2025 Oct 13;10(41):49052–60. doi: 10.1021/acsomega.5c07648 (PMC12547795; doi:10.1021/acsomega.5c07648)
Supplement: Supplementary file 1 [file ao5c07648_si_001.pdf]

Supporting Information

Dual-Emitting Cyclometalated Platinum Compounds with Isocyanide Ligands

Craig M. Anderson,*[a] Matthew W. Greenberg,[a] Christopher N. LaFratta,[a] Ryan Lum,[a] Everest I. Oppenheimer,[a] Teeka Duplessis,[a] Kris M. Tulloch,[a] Joseph M. Tanski,[b]

[a] Department of Chemistry & Biochemistry, Bard College, 30 Campus Road, Annandale-on-Hudson, NY, 12504, USA.

Email: canderso@bard.edu Phone: 845-752-2356. FAX: 845-752-2339

[b] Department of Chemistry, Vassar College, Poughkeepsie, NY, 12604, USA.

KEYWORDS: luminescence, TD-DFT; cyclometalation, dual emission, isocyanides

Table of Contents

Spectra of Compounds 1A-2D

**NMR, IR, UV/Vis, Emission Spectra**	**1-43**
**TA Traces**	**44-52**
**Low Temperature Emission Spectra**	**52-53**
**DFT Orbitals**	**54-60**
**TD-DFT**	**61-62**
**NTOs**	**63-64**
**Excited State Dynamics**	**65**
**DFT Data Tables**	**66**
**References**	**67**

Table S1. Compound names and structures

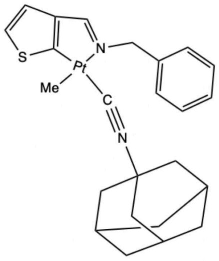 **1A**	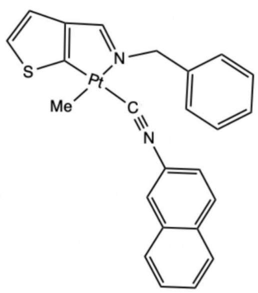 **1B**	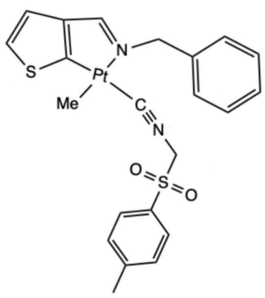 **1C**	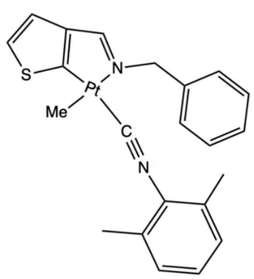 **1D**
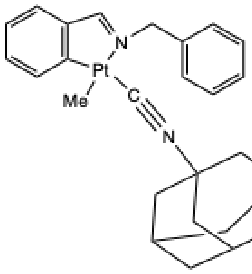 **2A**	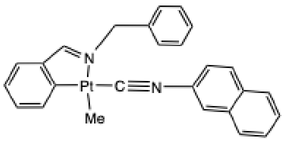 **2B**	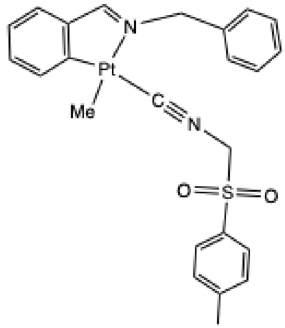 **2C**	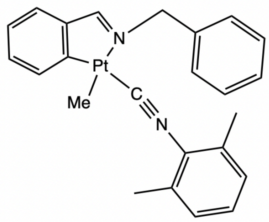 **2D**

Figure S1. Compound **1A** ^{1}H NMR

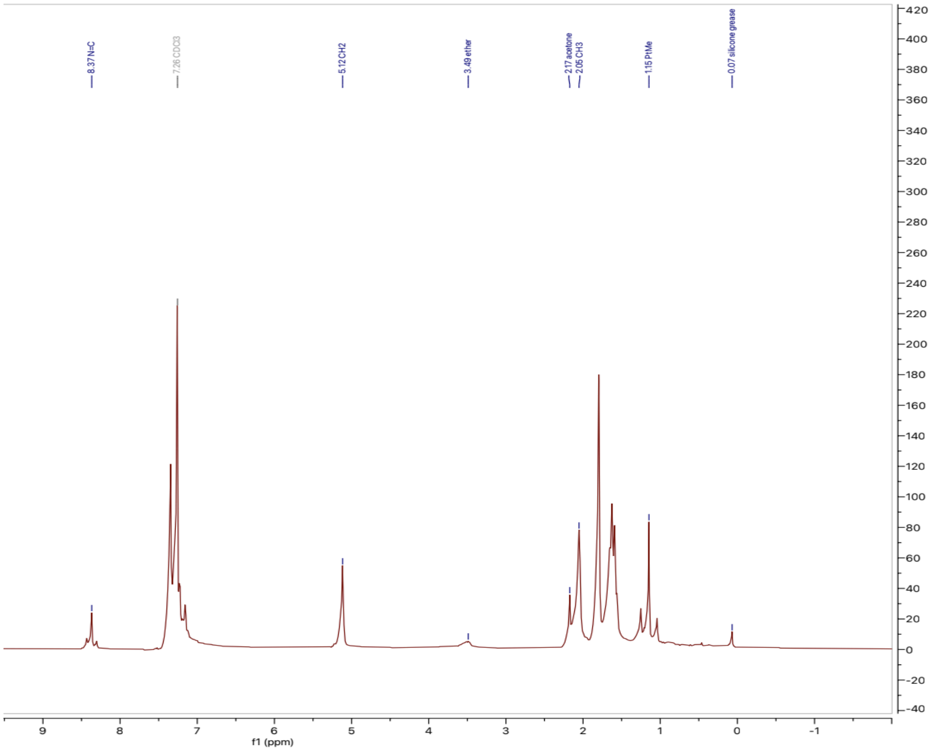

Figure S2. Compound **1A** COSY

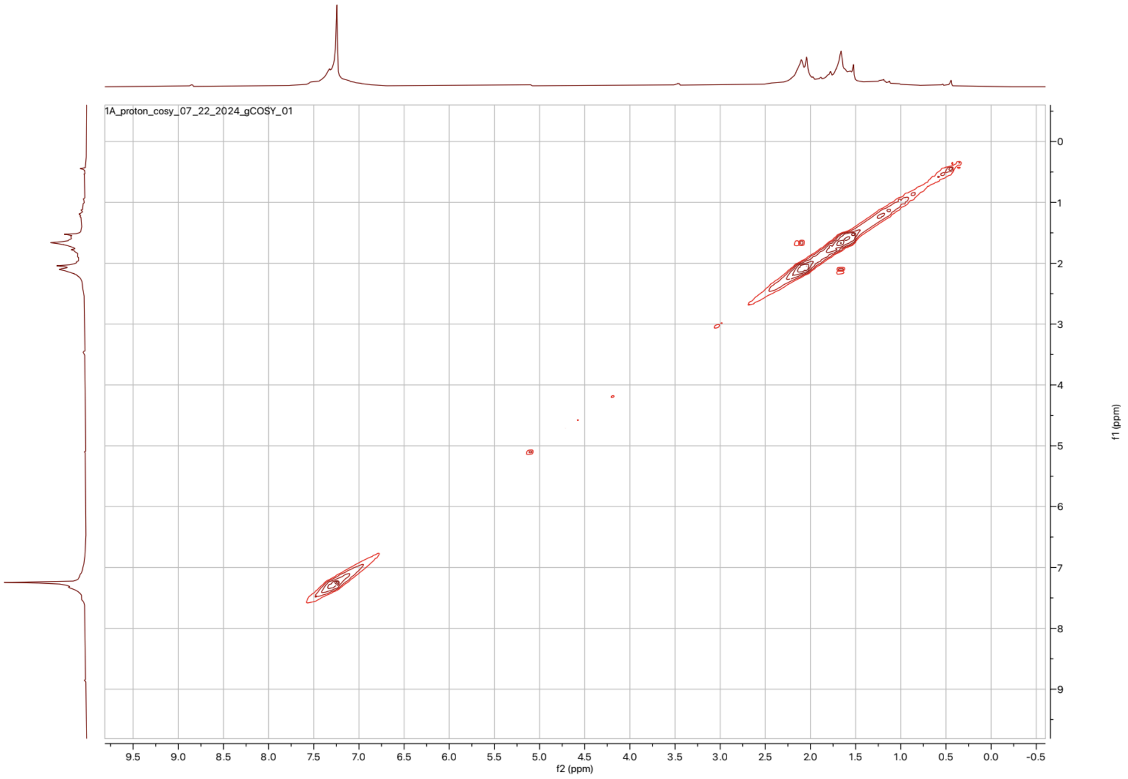

Figure S3. Compound **1A** ^{13}C-NMR

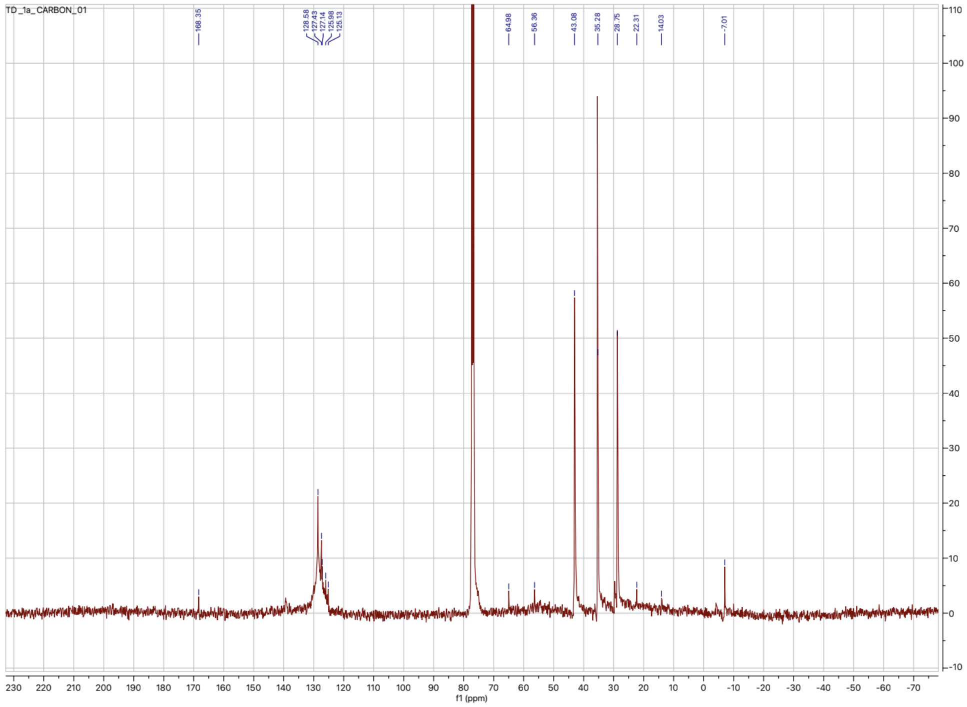

Figure S4. Compound **1A** HSQC

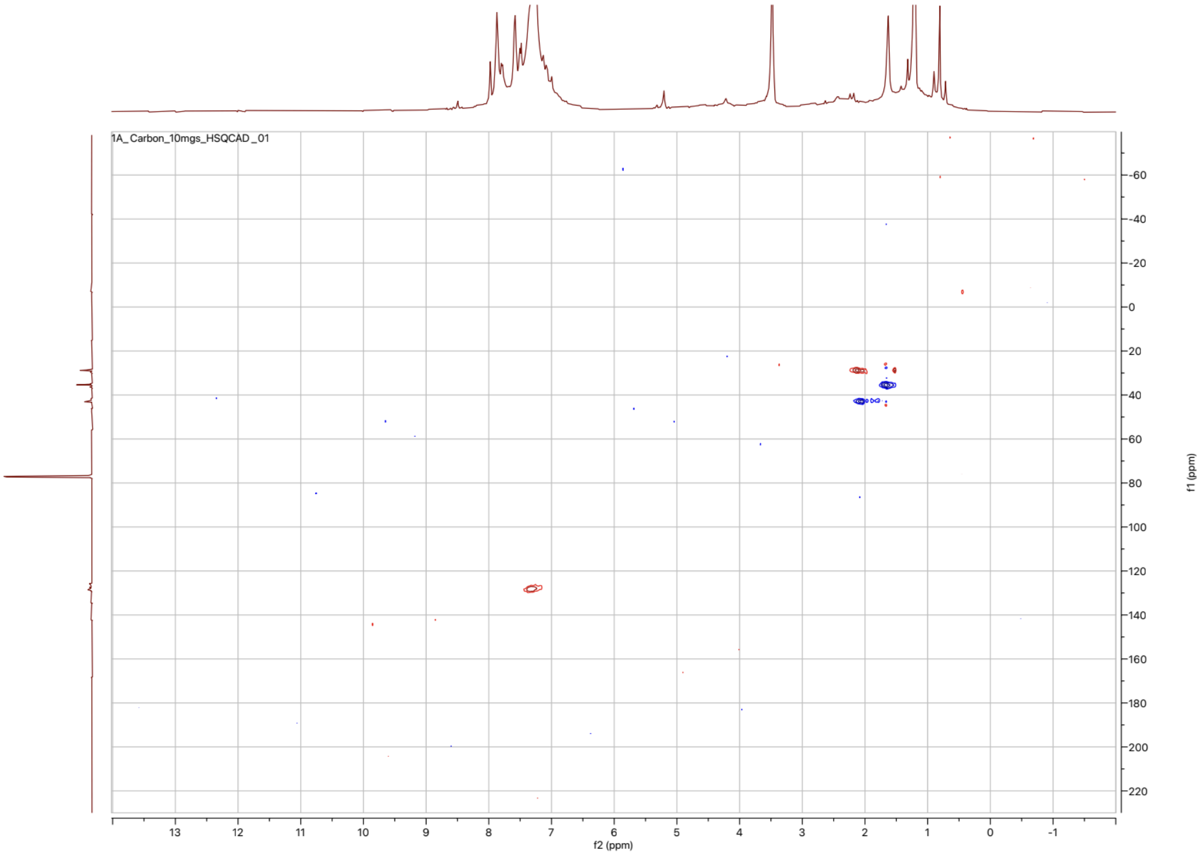

Figure S5. Compound **1A** emission (DCM)

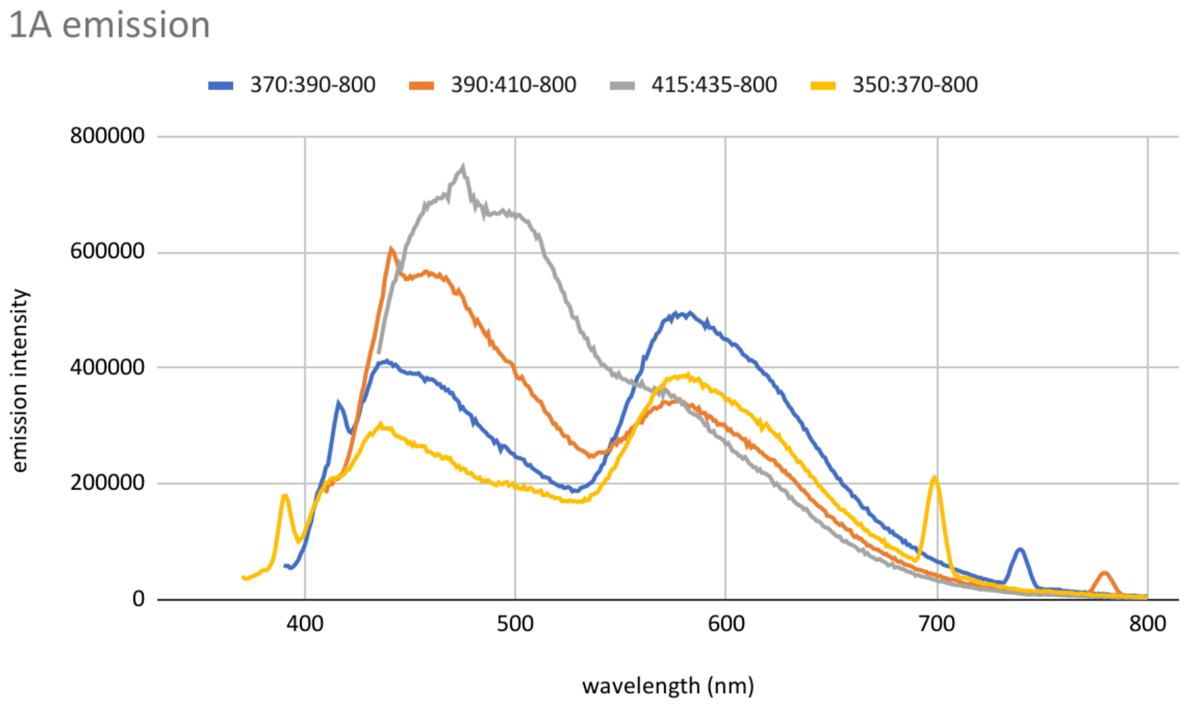

Figure S6. Compound **1A** Uv-vis (DCM)

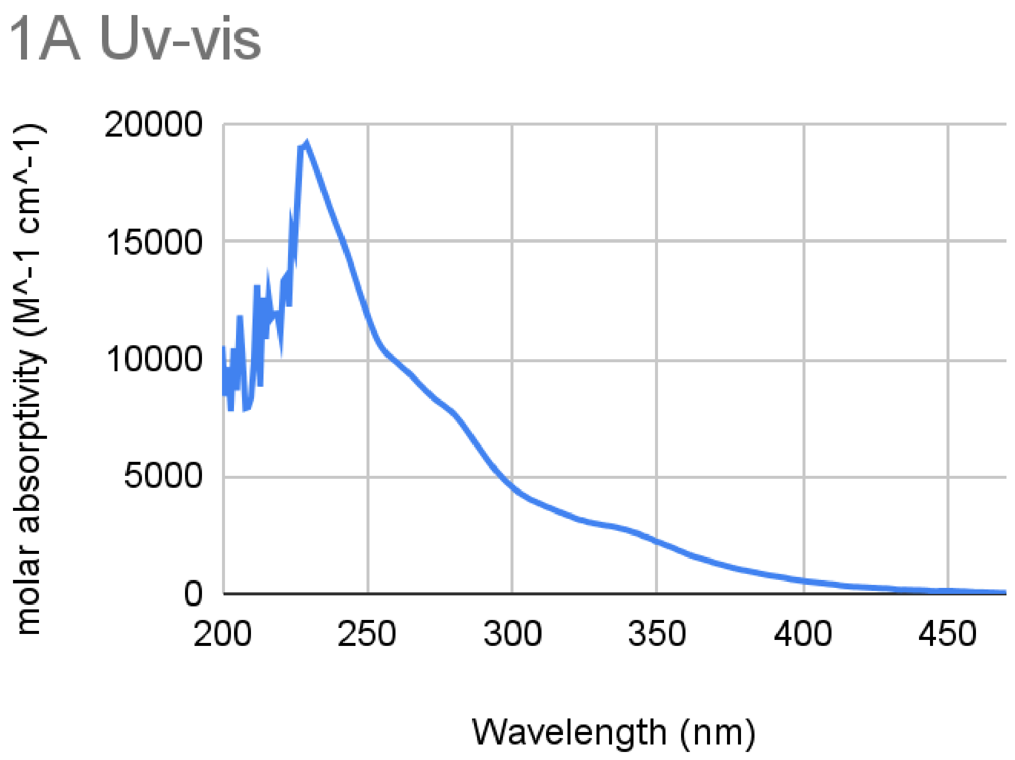

Figure S7. Compound **1A** IR (diamond)

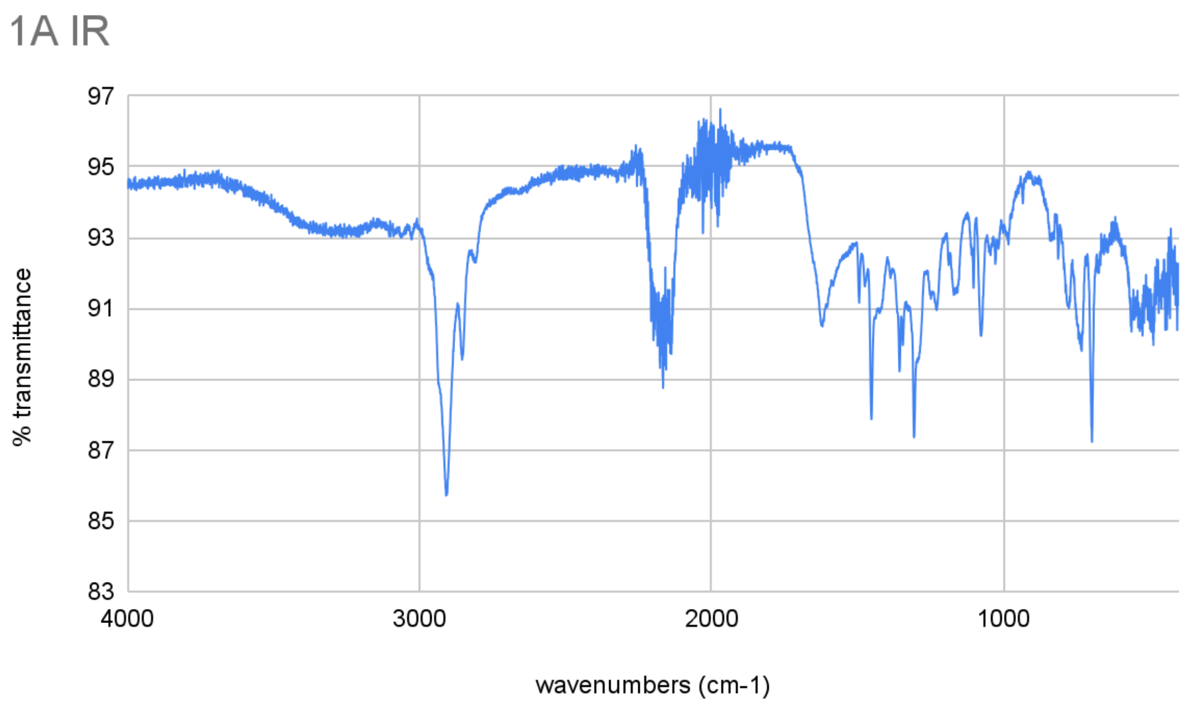

Figure S8. Compound **1A** IR (Ge)

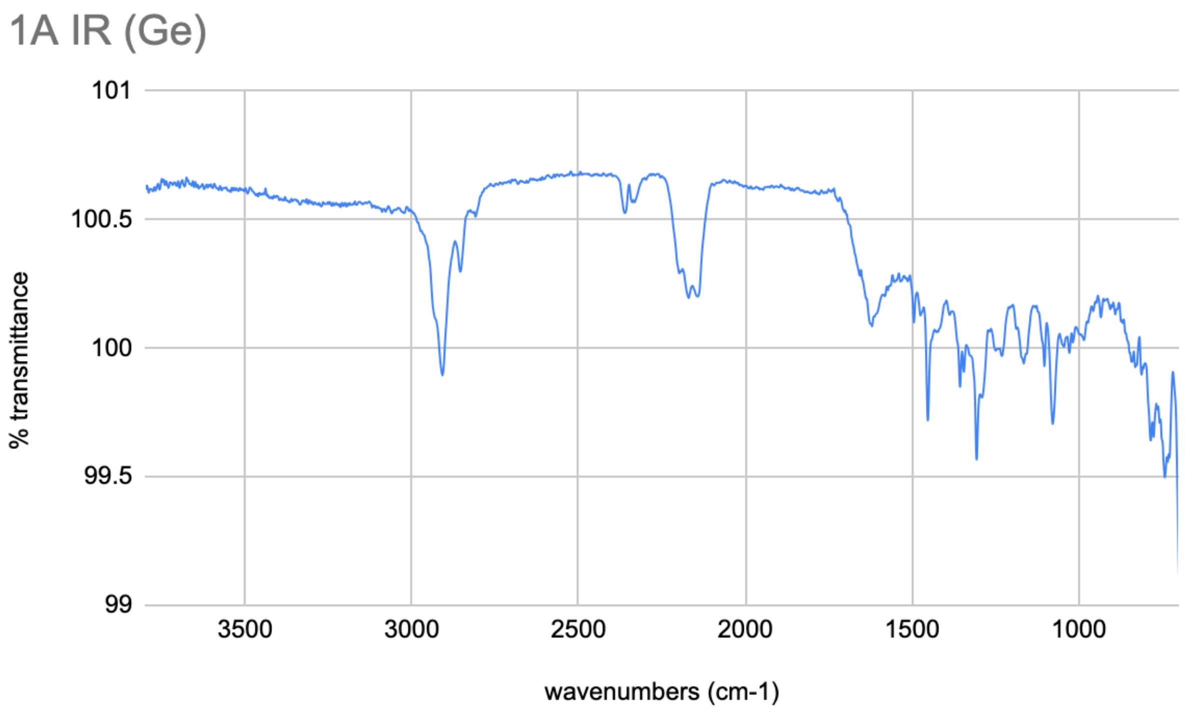

Figure S9. Compound **1B** ^{1}H NMR

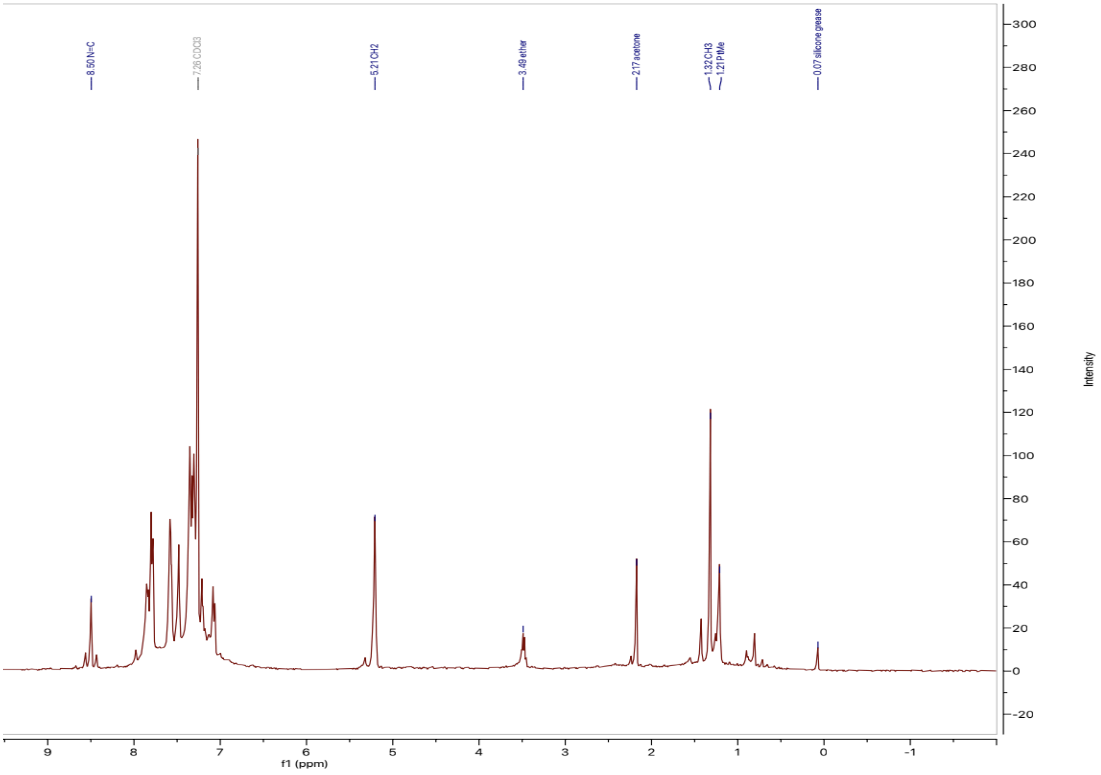

Figure S10. Compound **1B** COSY

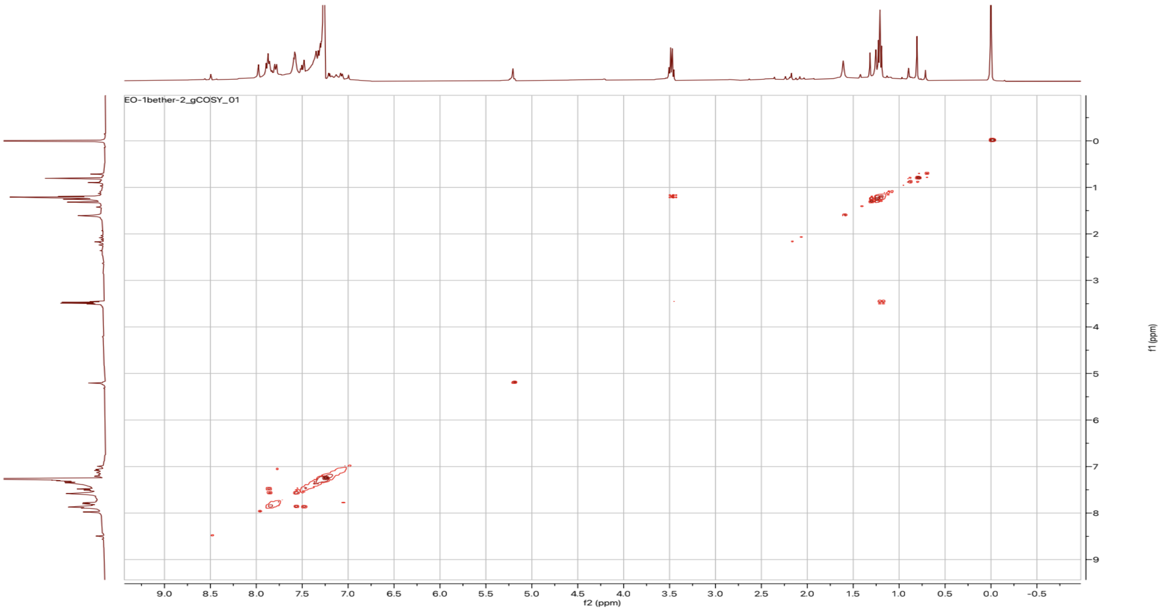

Figure S11. Compound **1B** ^{13}C-NMR

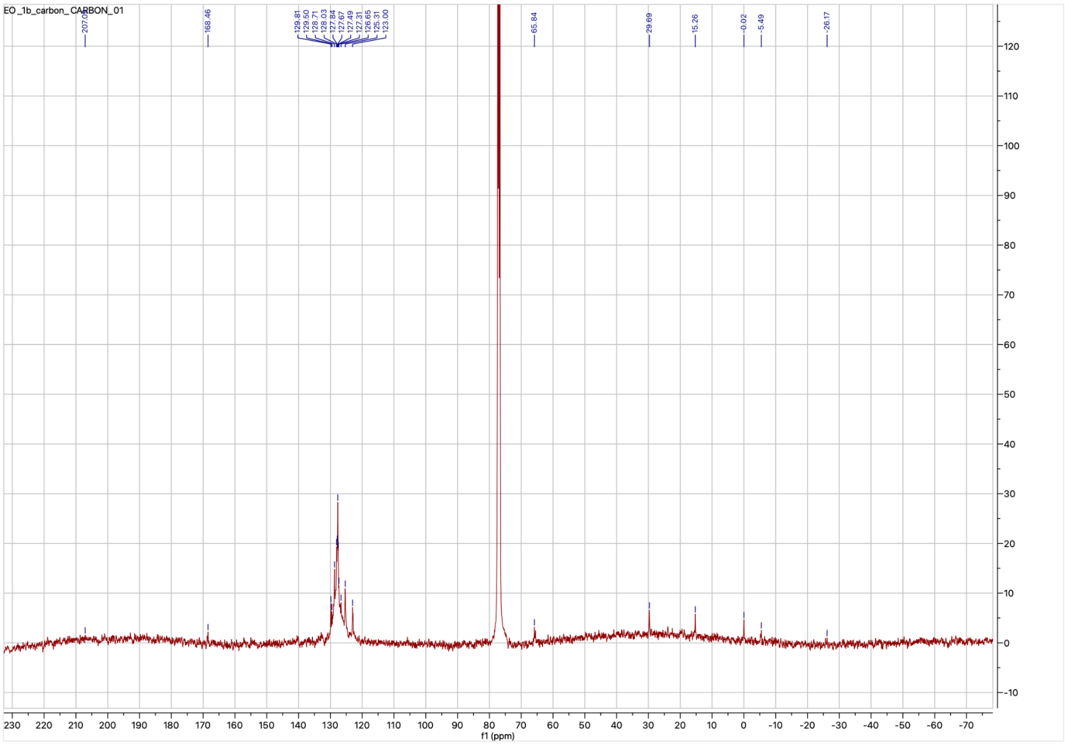

Figure S12. Compound **1B** emission (DCM)

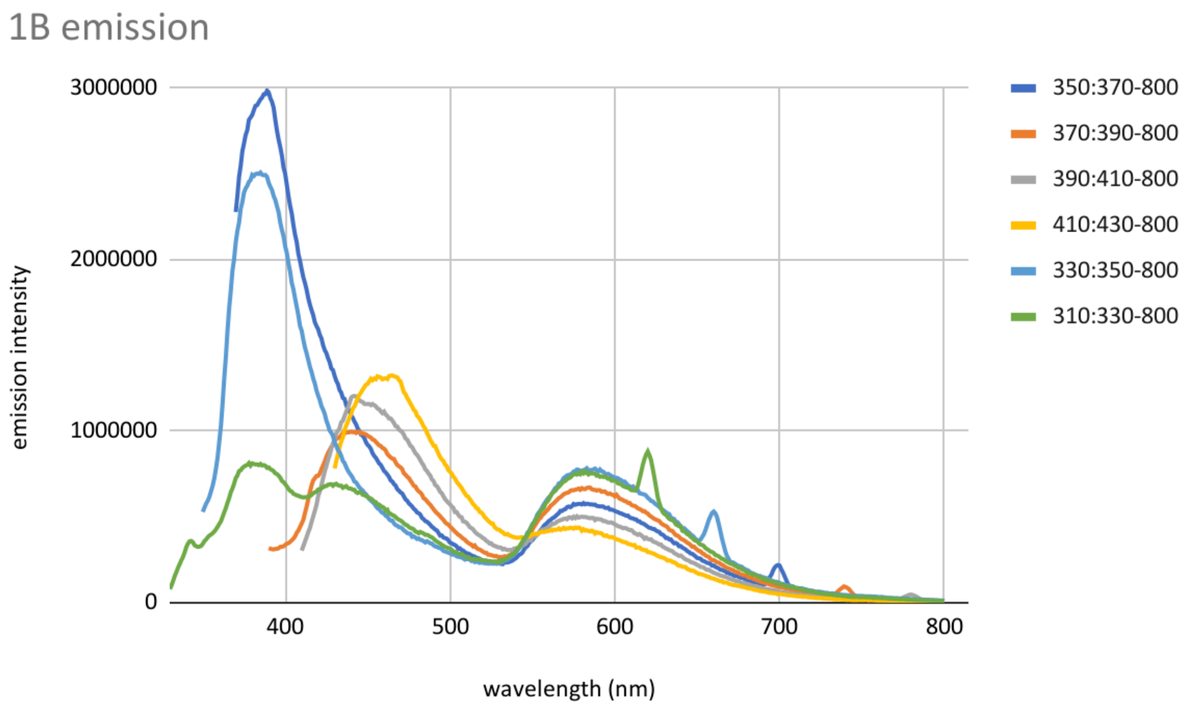

Figure S13. Compound **1B** Uv-vis (DCM)

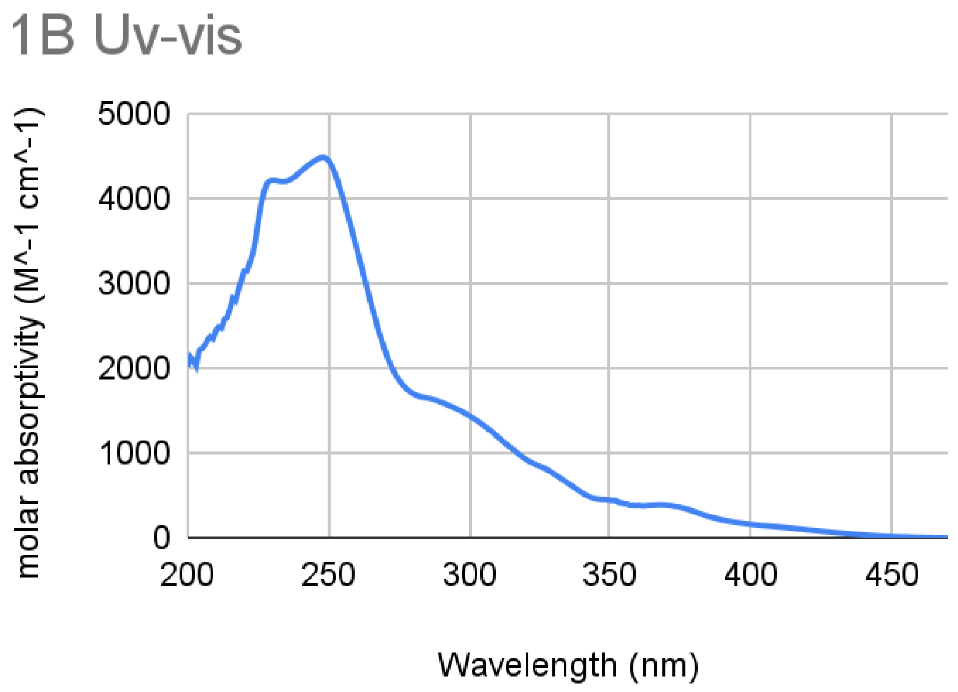

Figure S14. Compound **1B** IR (diamond)

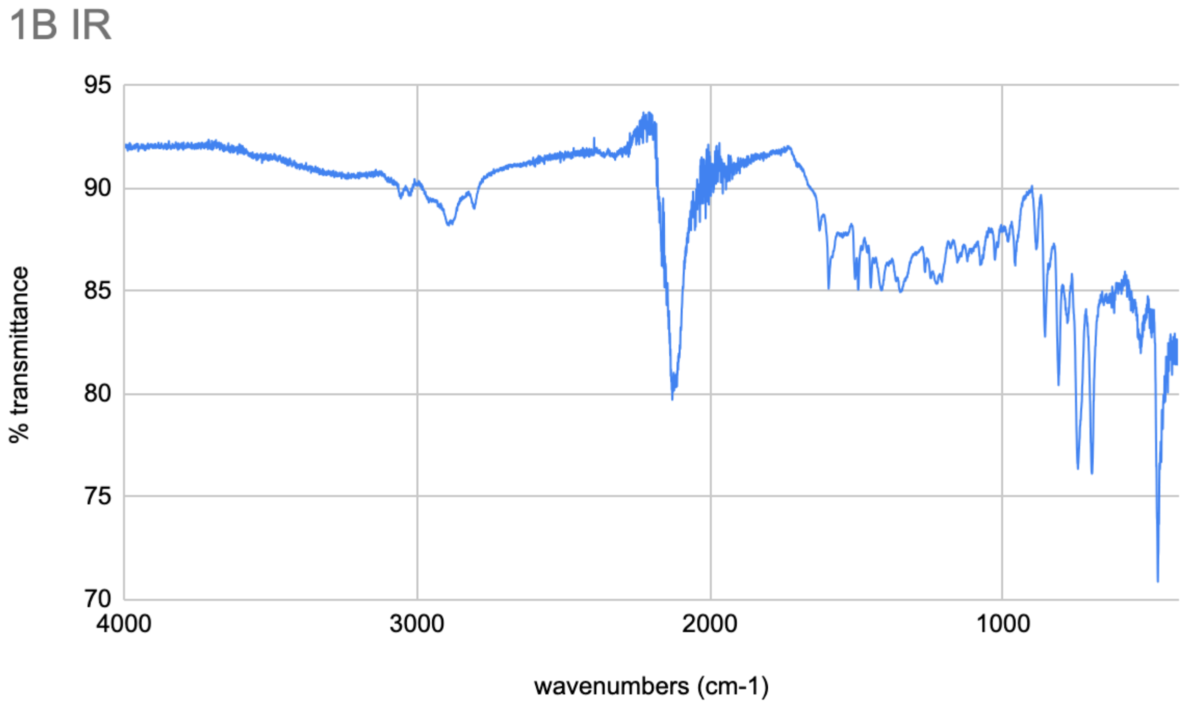

Figure S15. Compound **1B** IR (diamond)

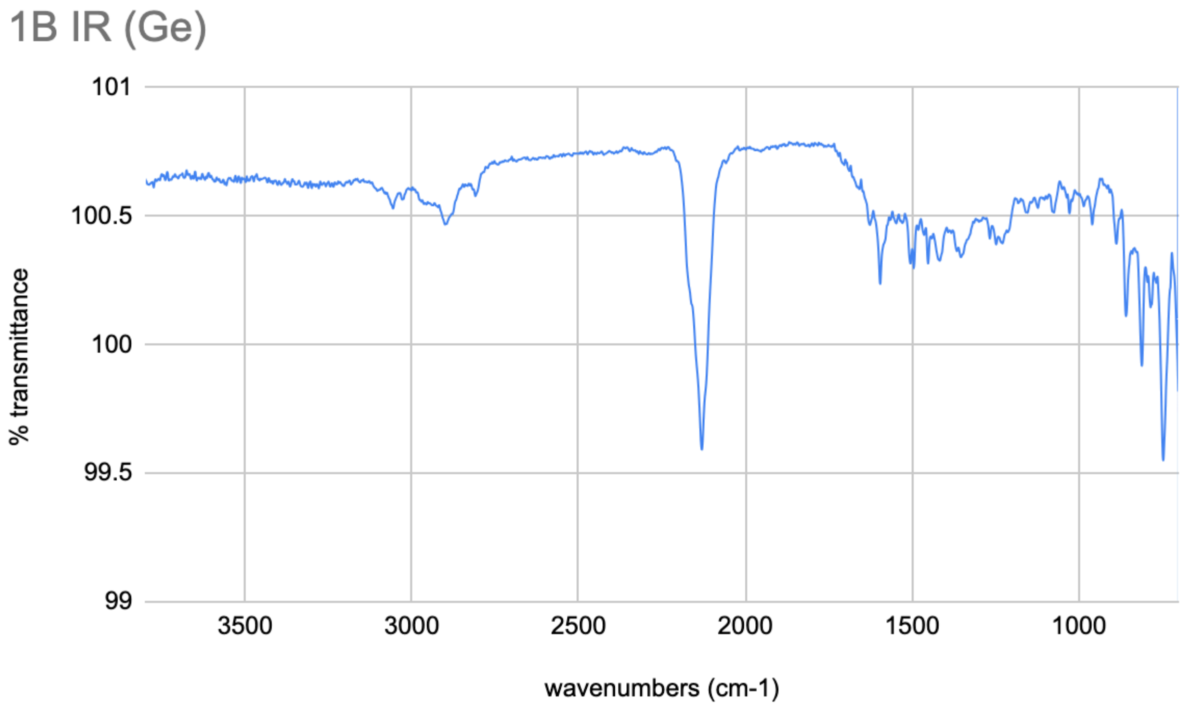

Figure S16. Compound 1C ^{1}H NMR

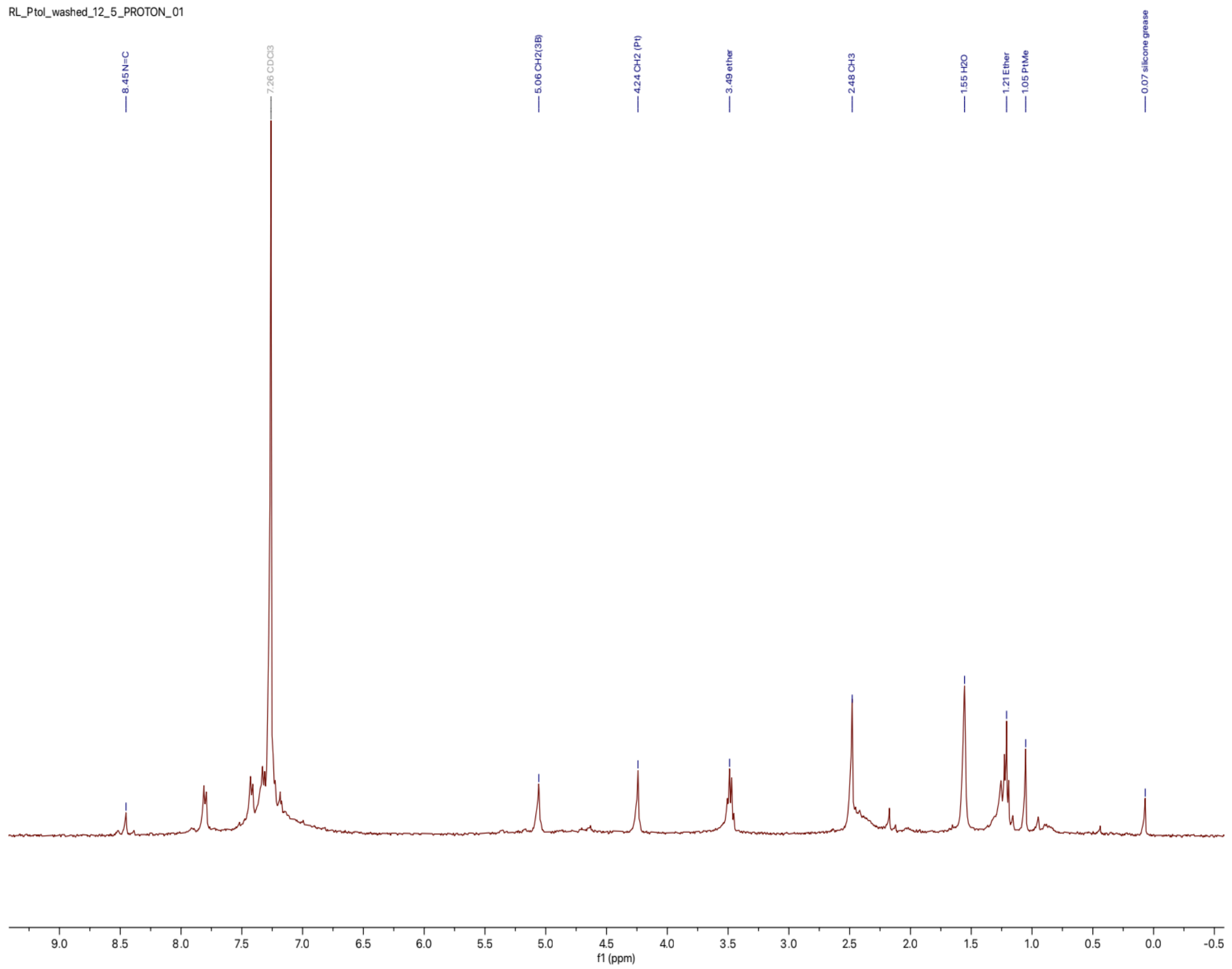

Figure S17. Compound **1C** COSY

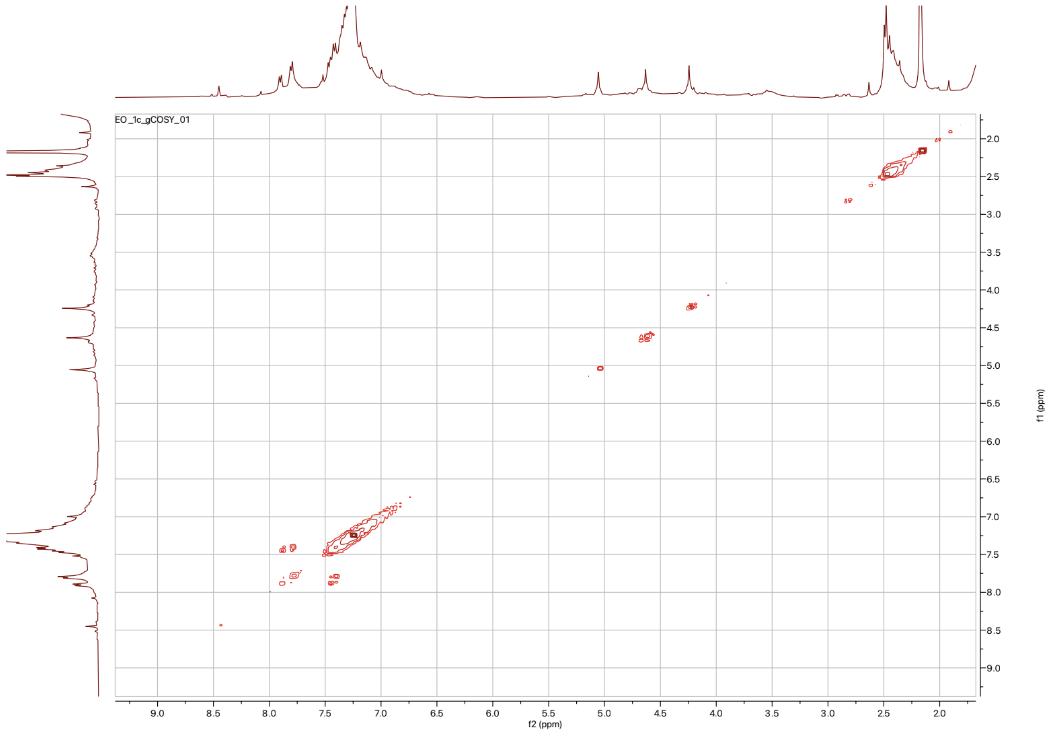

Figure S18. Compound **1C** ^{13}C-NMR

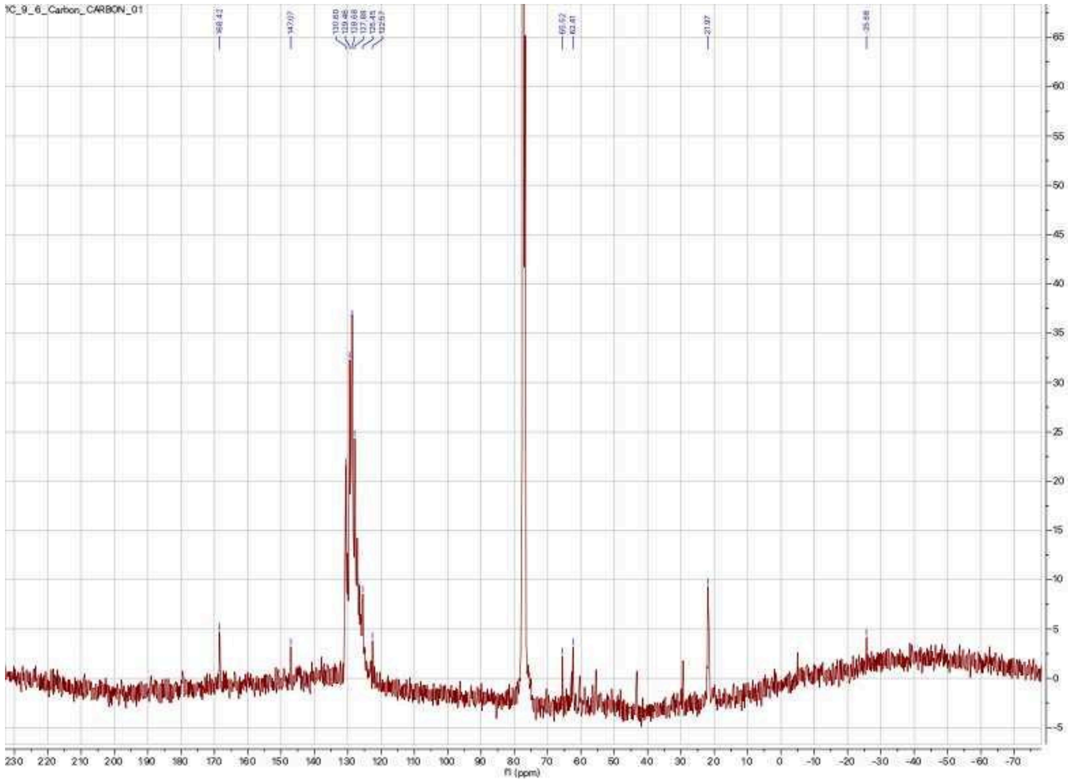

Figure S19. Compound **1C** HSQC

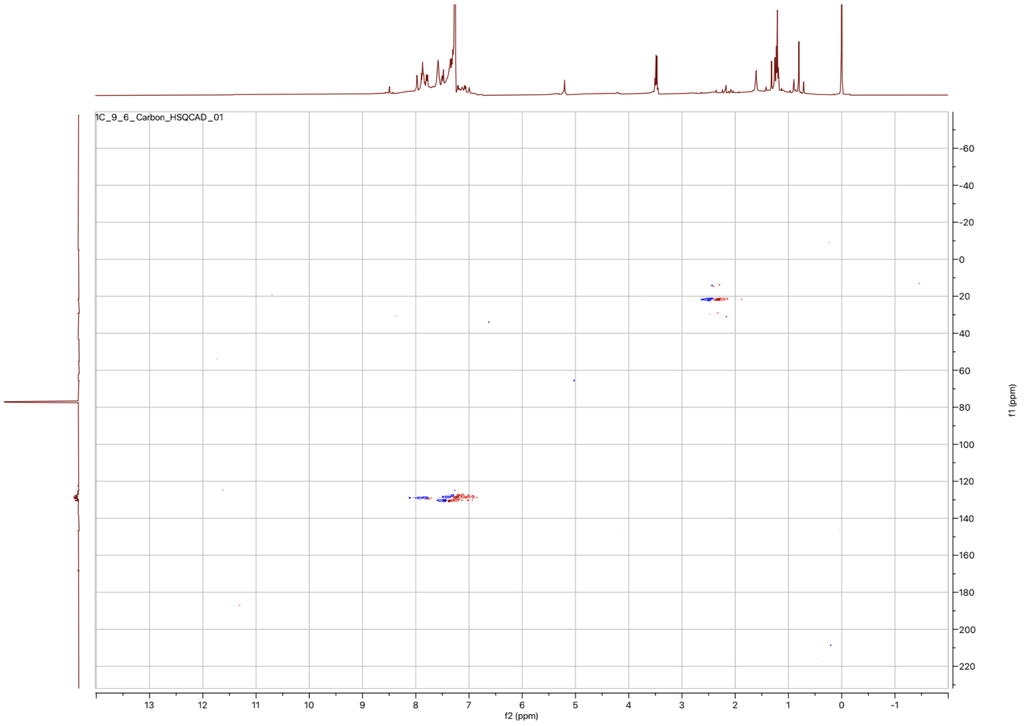

Figure S20. Compound **1C** emission (DCM)

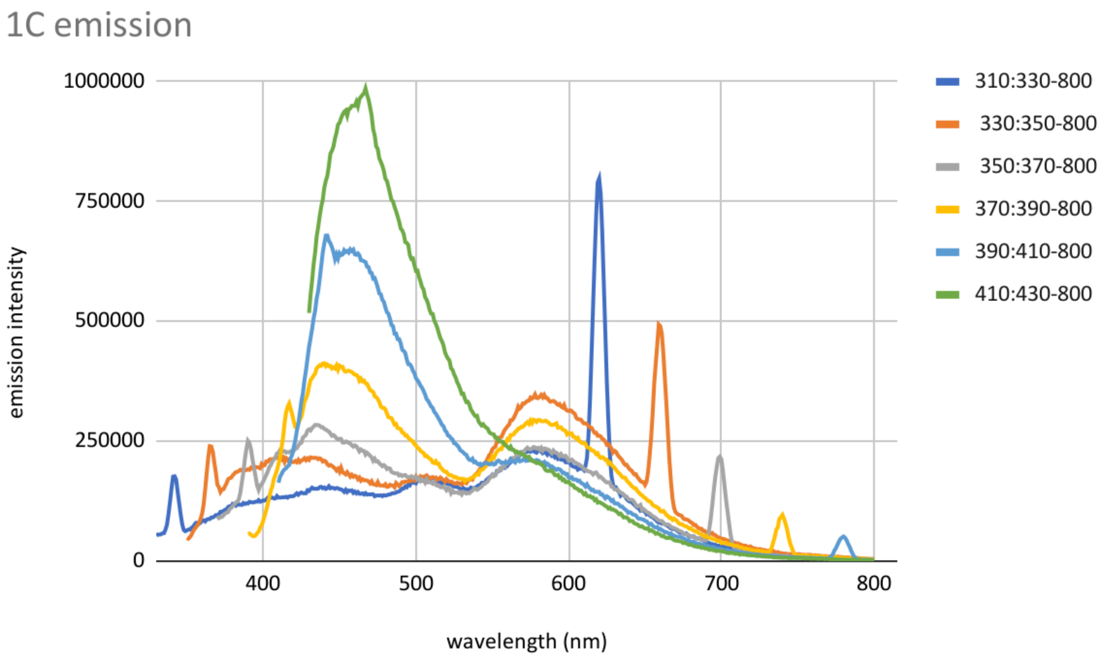

Figure S21. Compound **1C** Uv-vis (DCM)

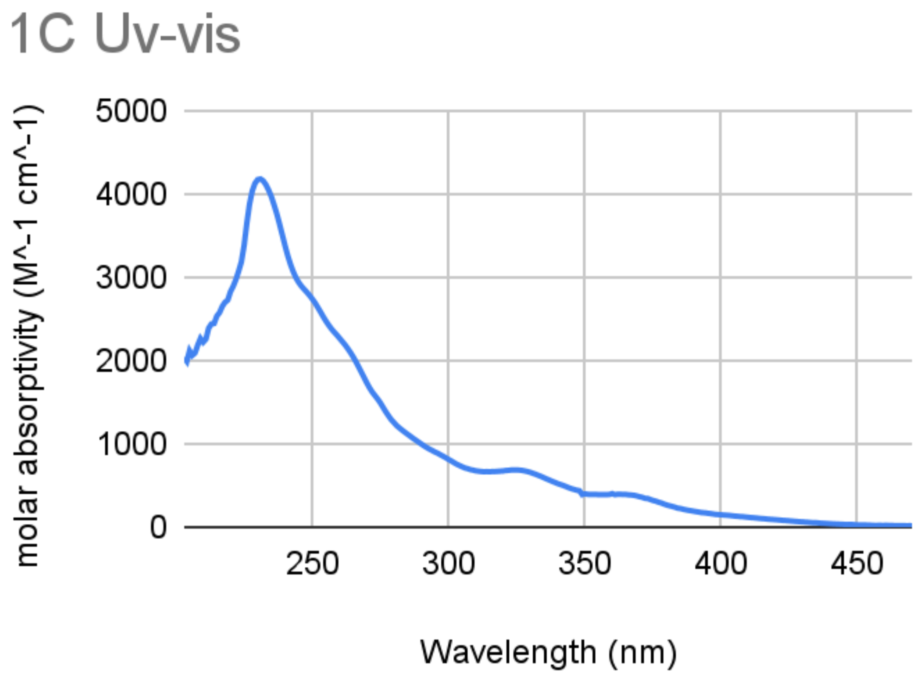

Figure S22. Compound **1C** IR (diamond)

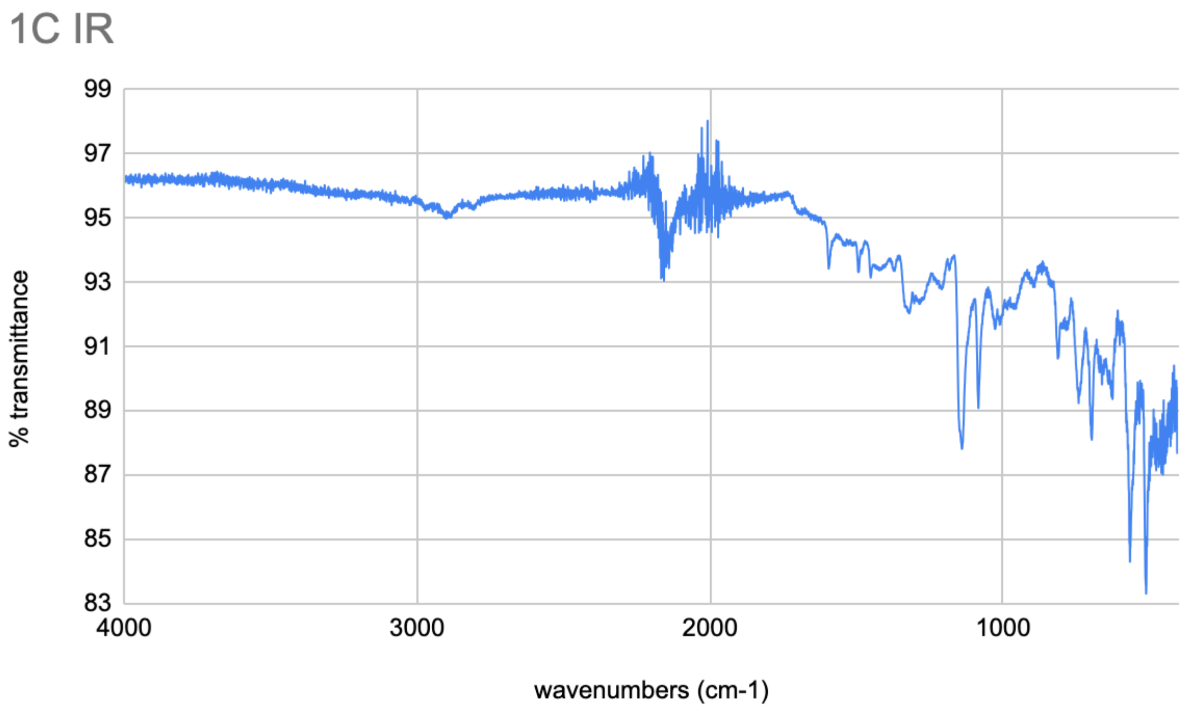

Figure S23. Compound **1C** IR (Ge)

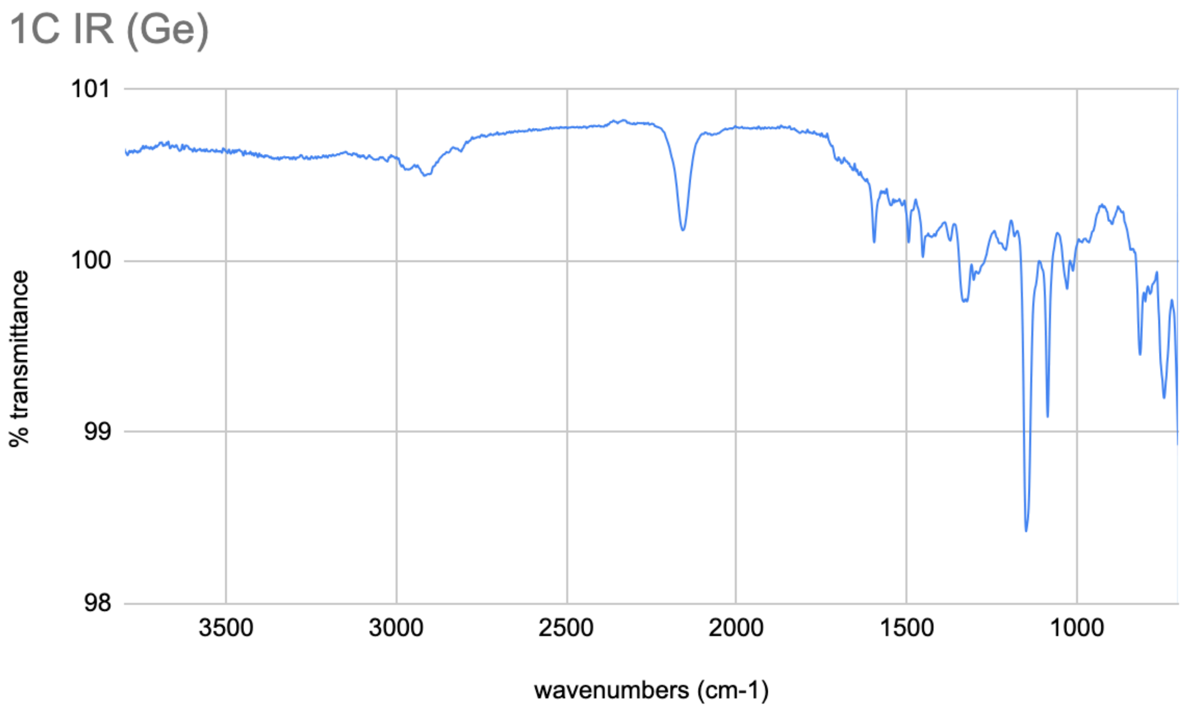

Figure S24. Compound **1D** ^{1}H NMR

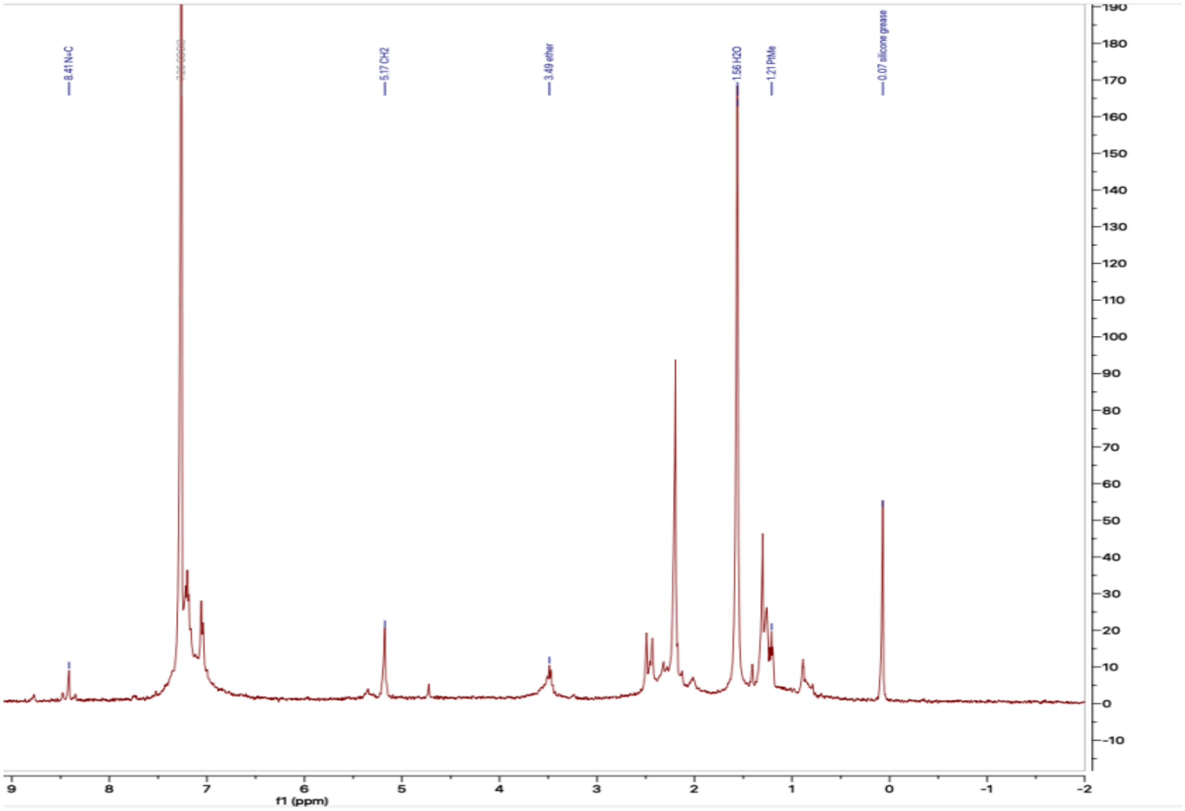

Figure S25. Compound **1D** COSY

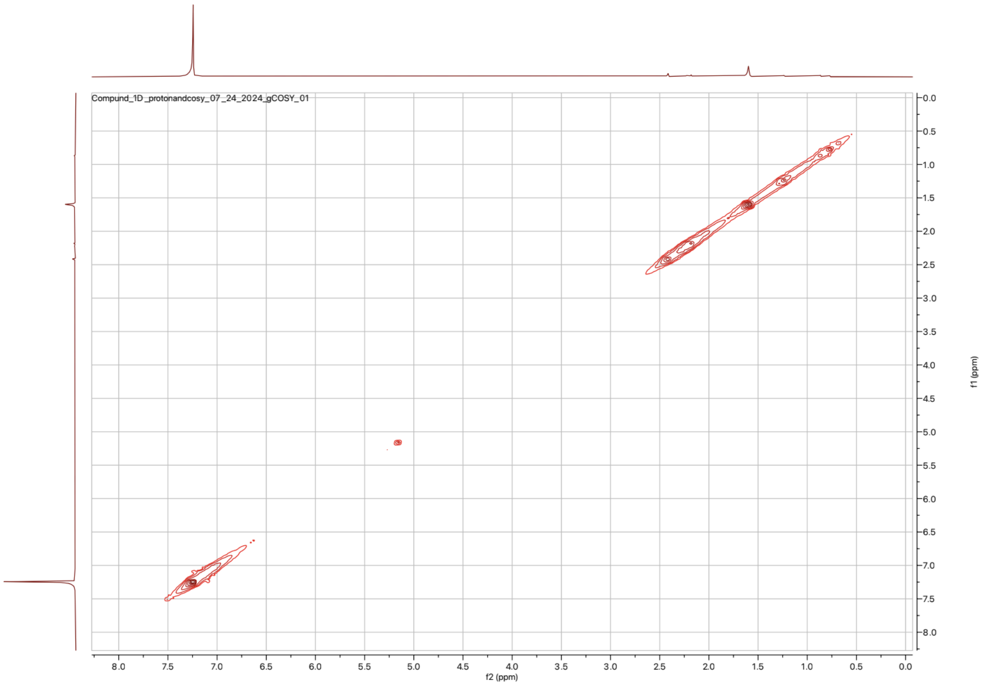

Figure S26. Compound **1D** emission (DCM)

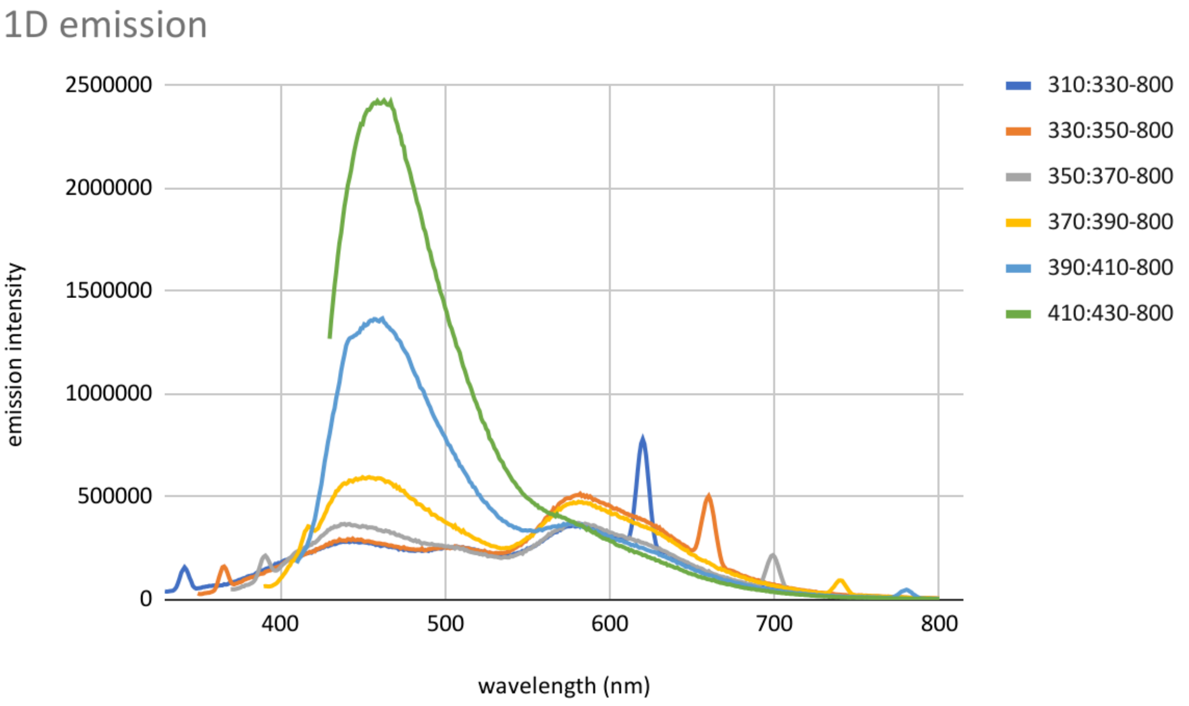

Figure S27. Compound **1D** Uv-vis (DCM)

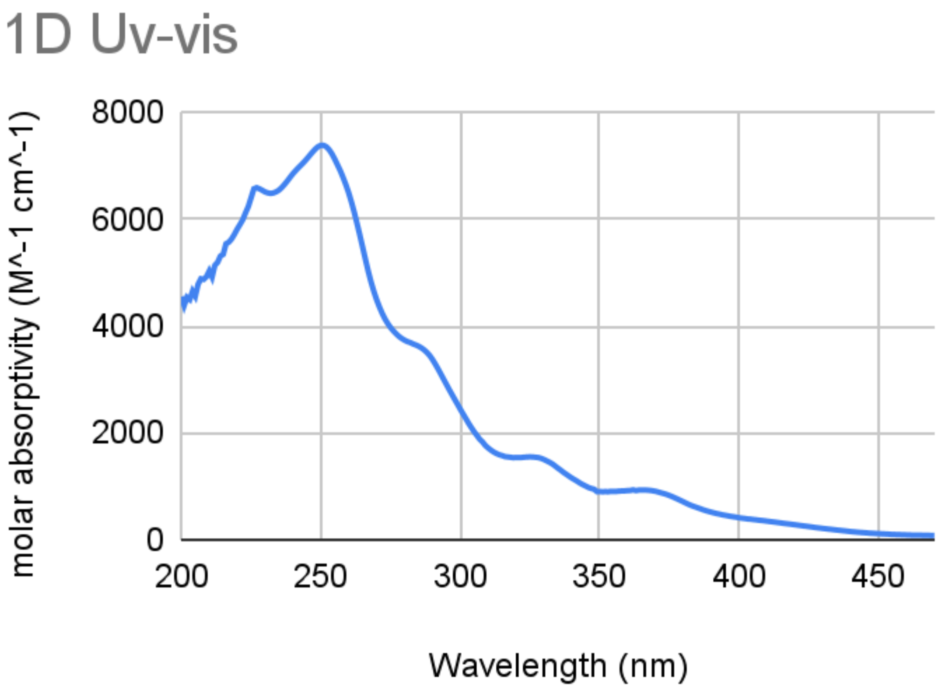

Figure S28. Compound **1D** IR (diamond)

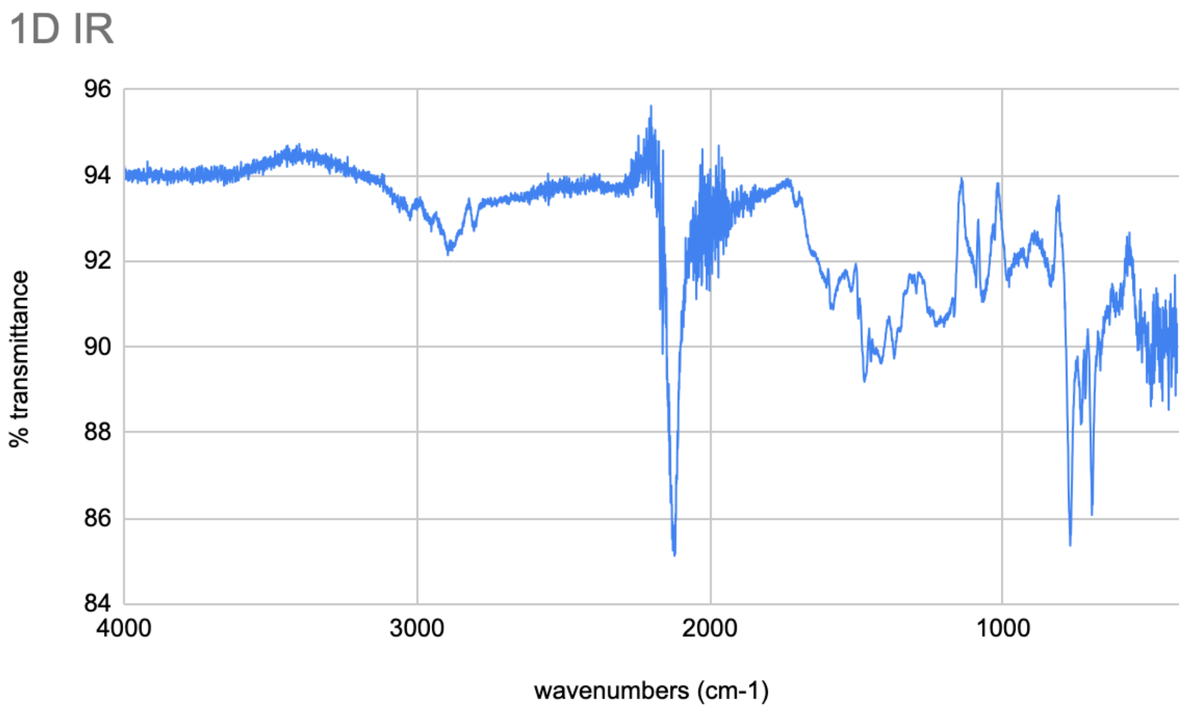

Figure S29. Compound **1D** IR (Ge)

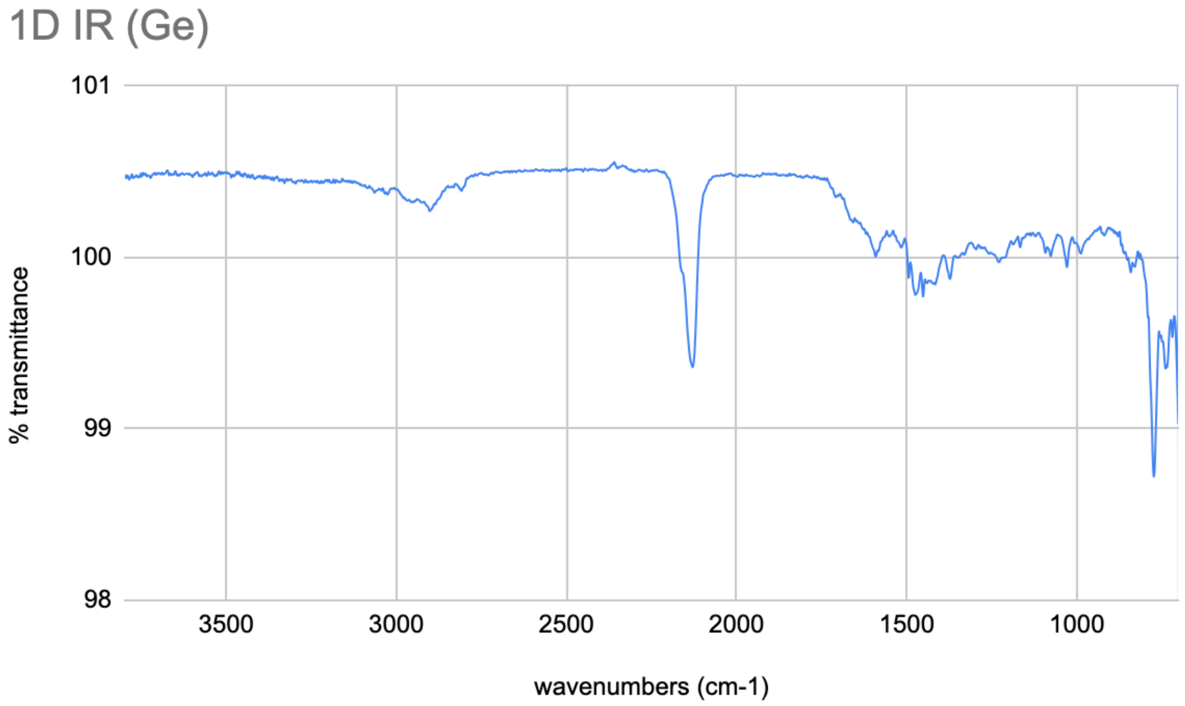

Figure S30. Compound **2A** ^{1}H NMR

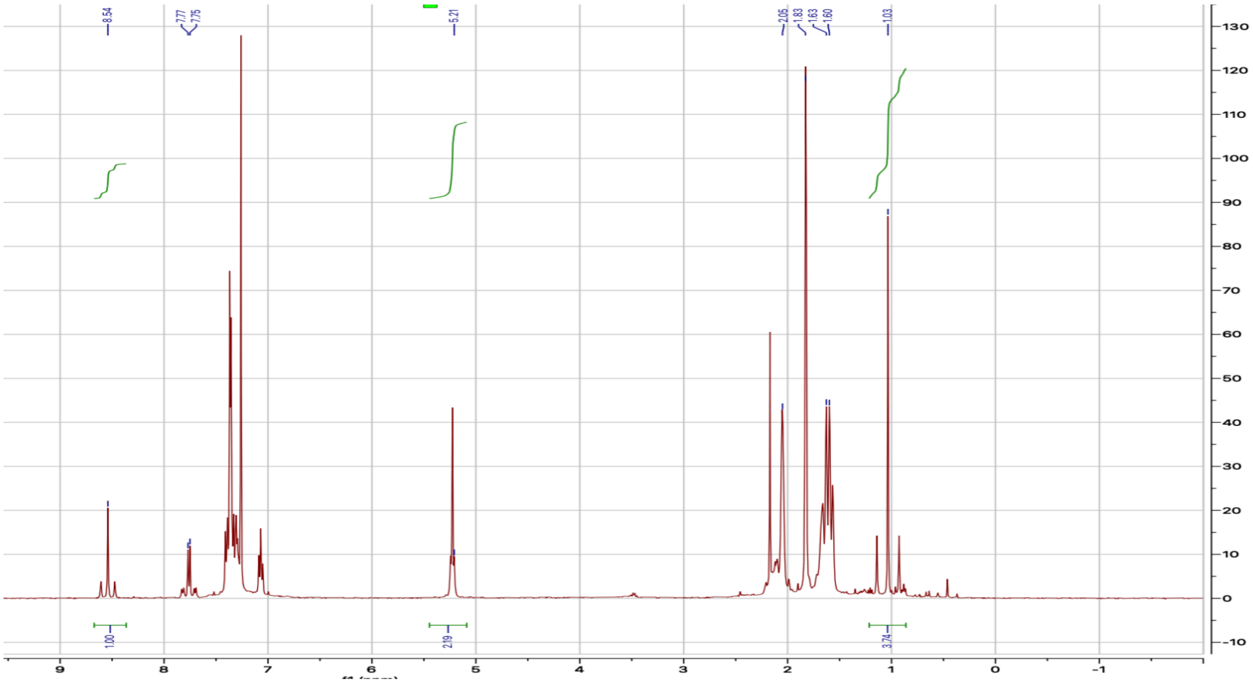

Figure S31. Compound **2A** COSY

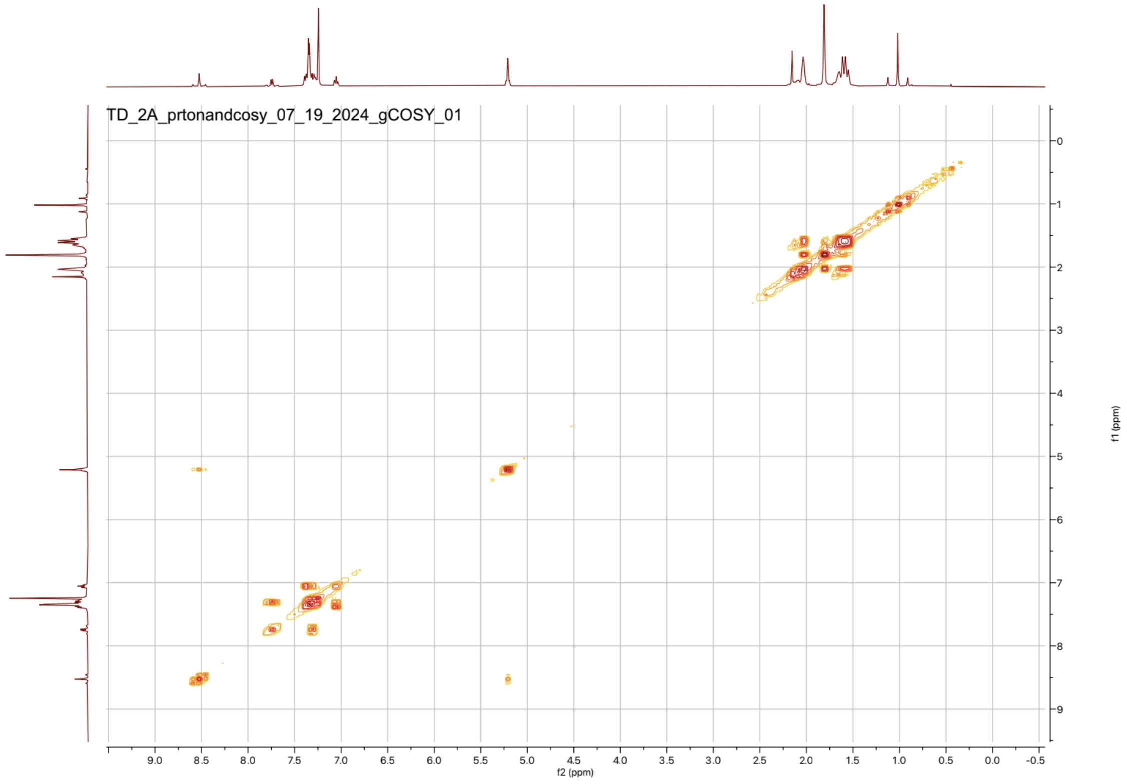

Figure S32. Compound **2A** ^{13}C-NMR

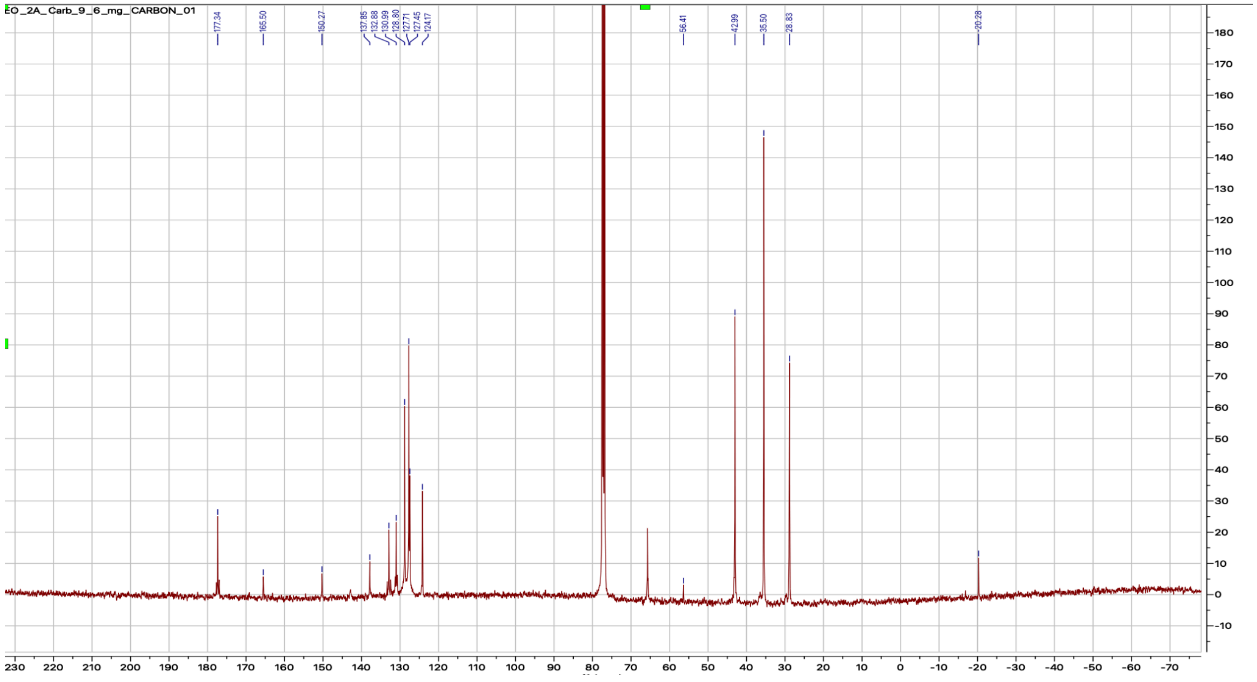

Figure S33. Compound **2A** HSQC

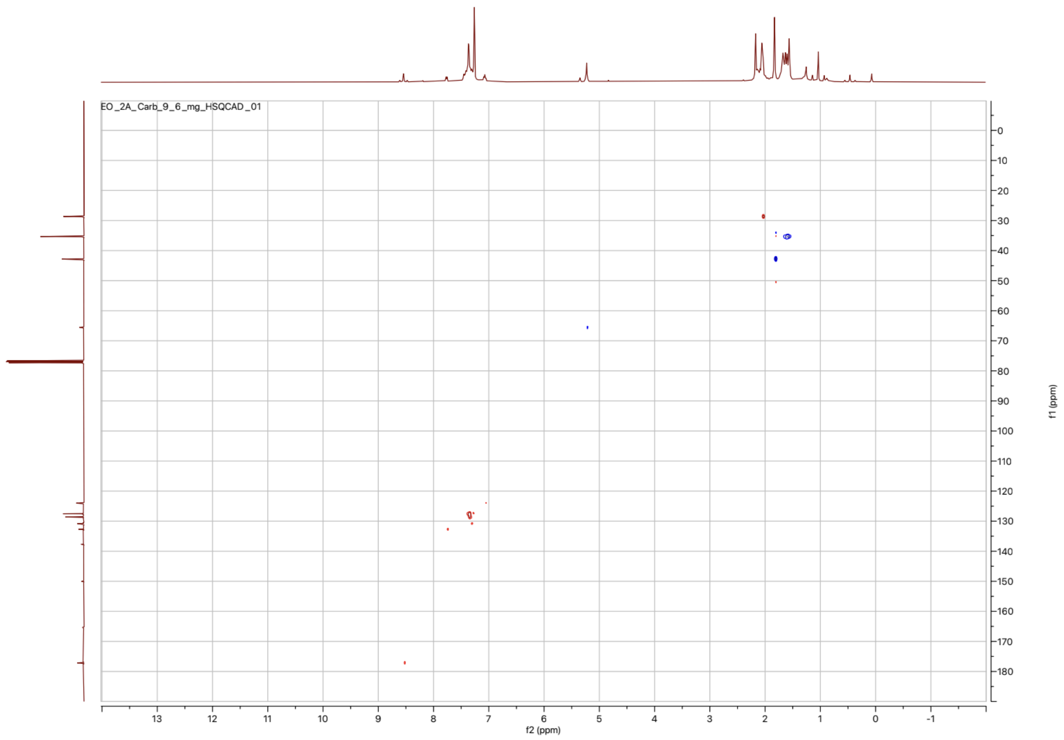

Figure S34. Compound **2A** emission (DCM)

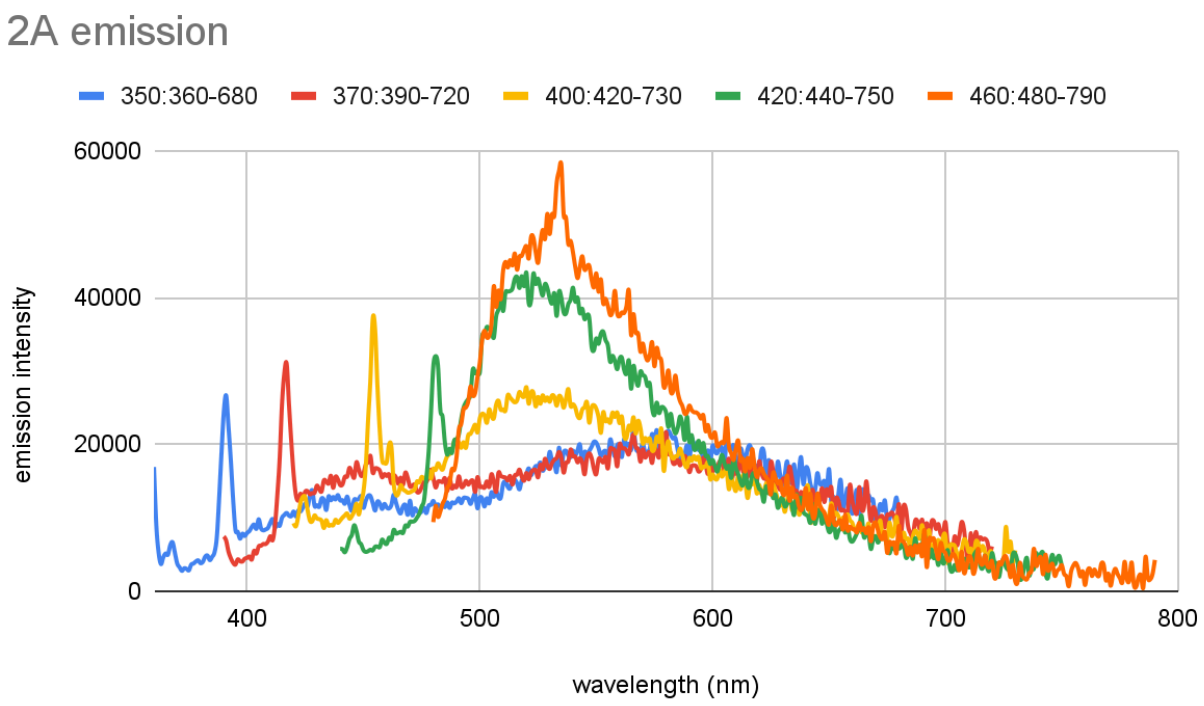

Figure S35. Compound **2A** Uv-vis (DCM)

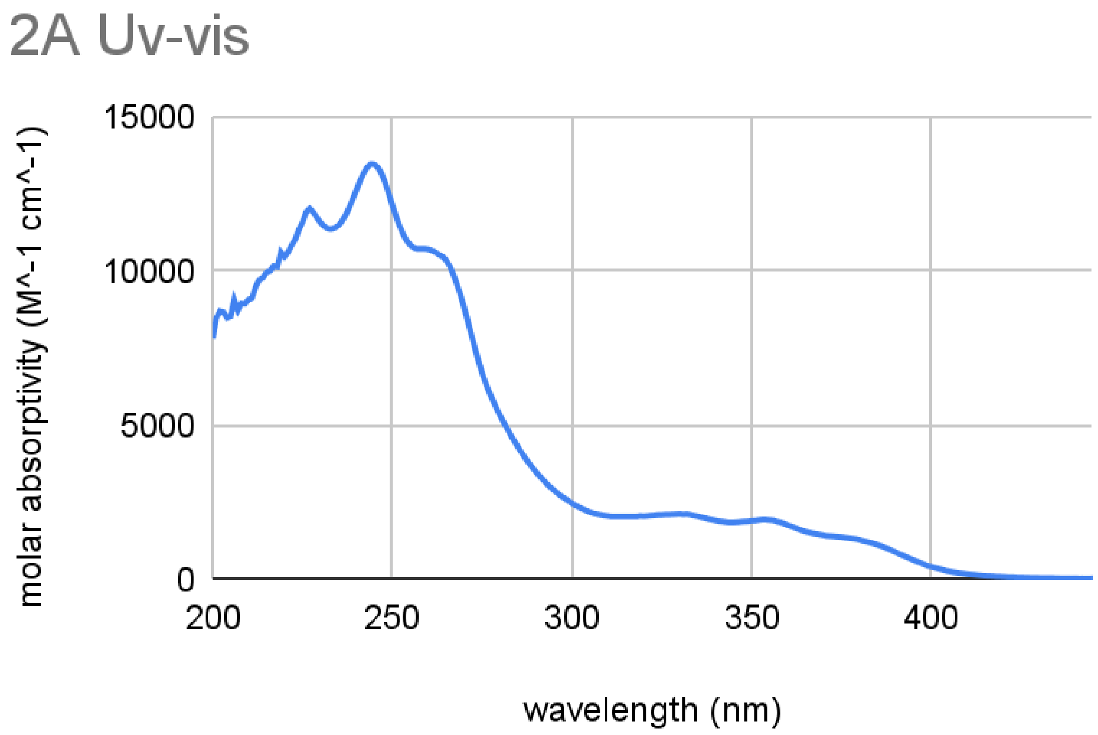

Figure S36. Compound **2A** IR (diamond & Ge)

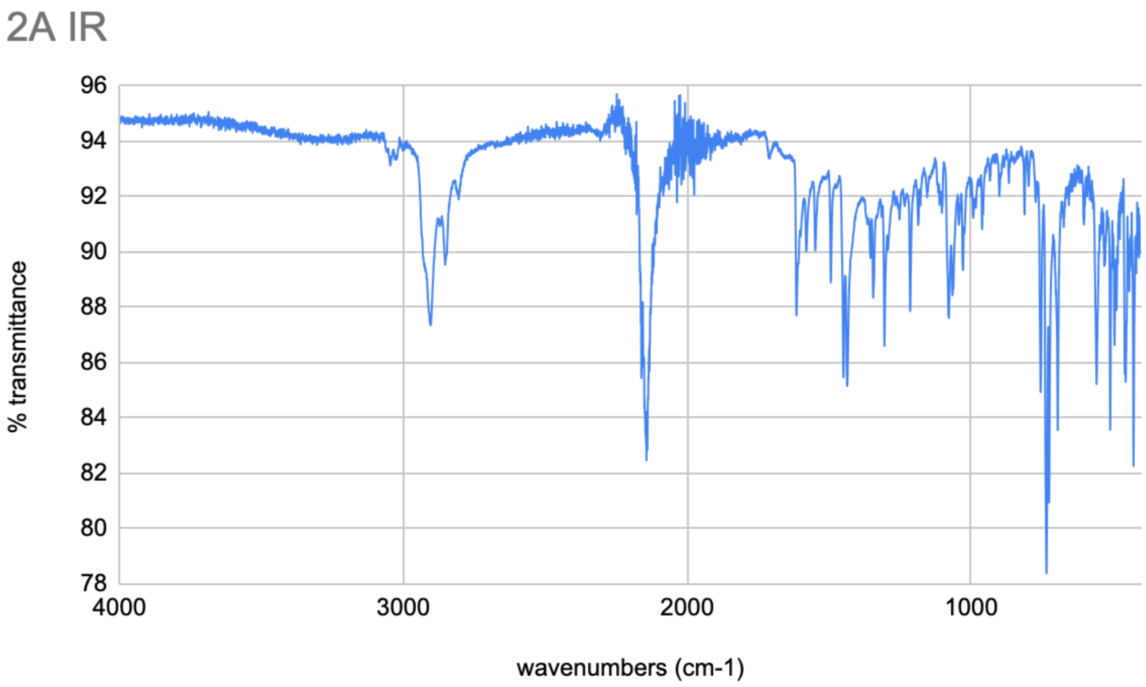

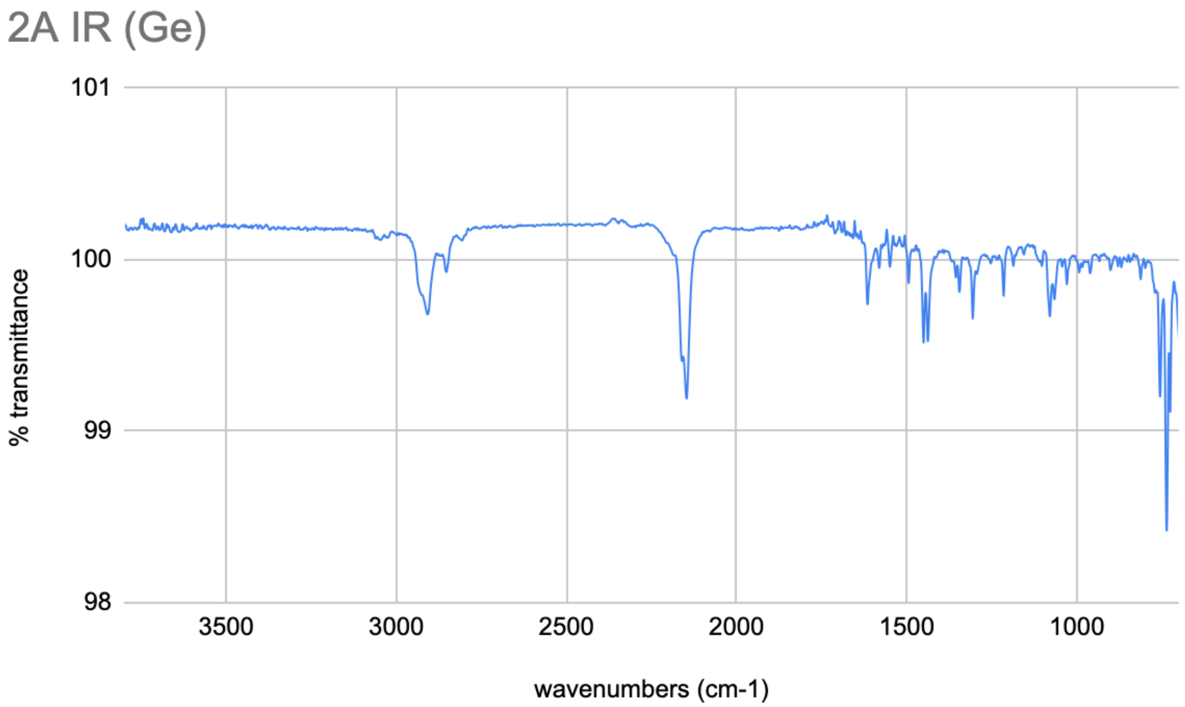

Figure S37. Compound **2B** ^{1}H NMR

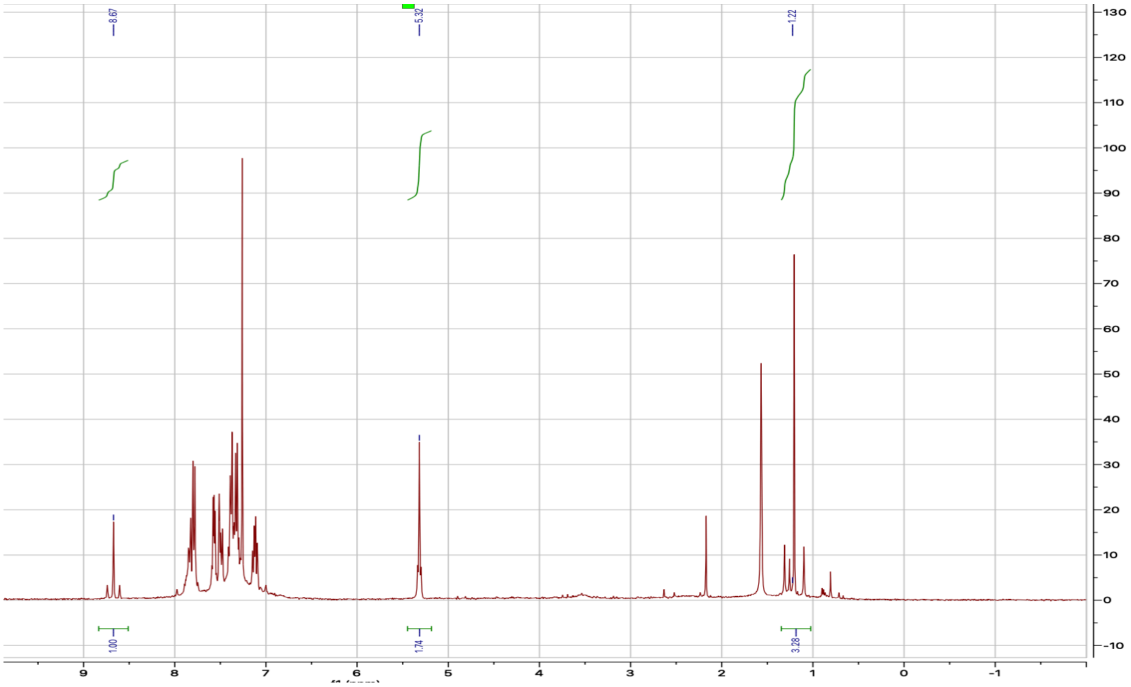

Figure S38. Compound **2B** COSY

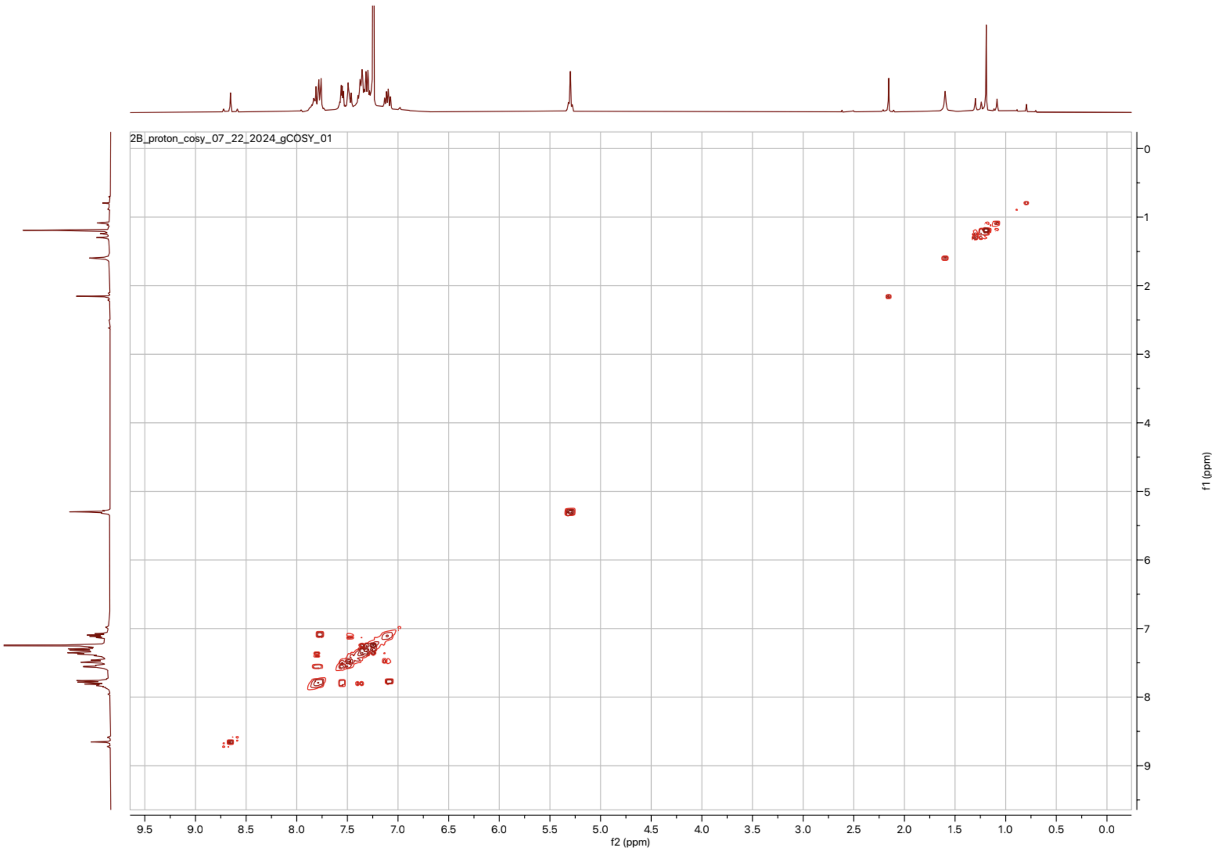

Figure S39. Compound **2B** ^{13}C-NMR

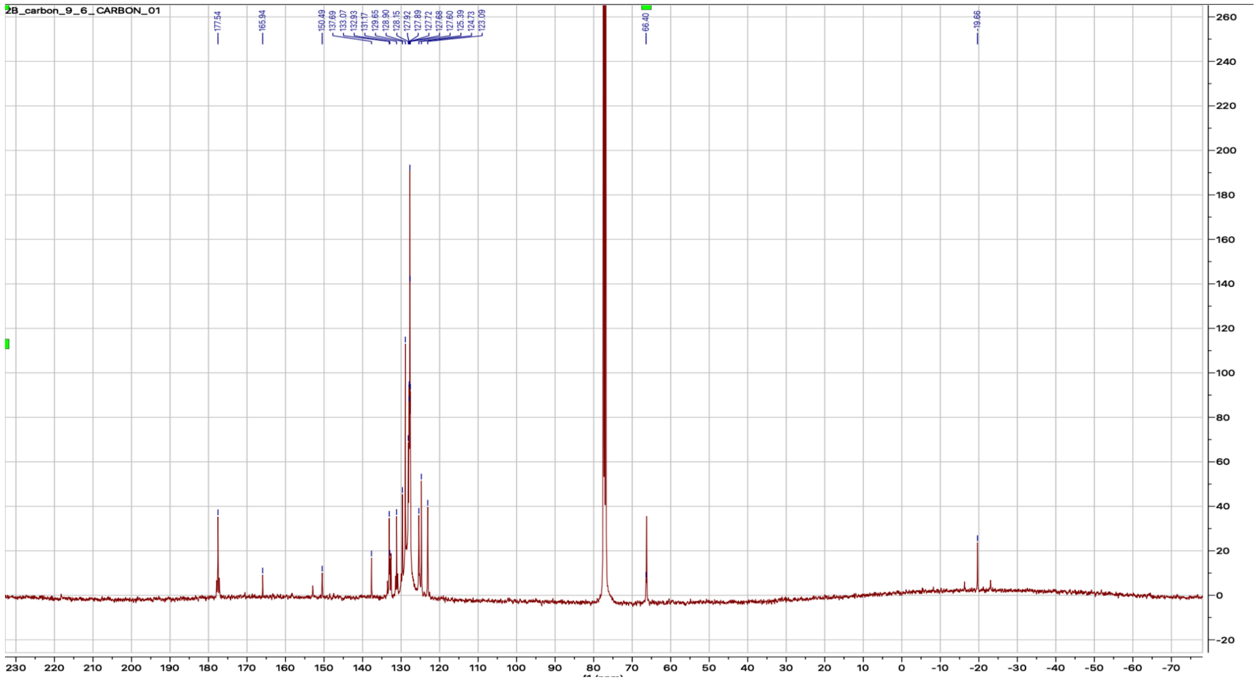

Figure S40. Compound **2B** HSQC

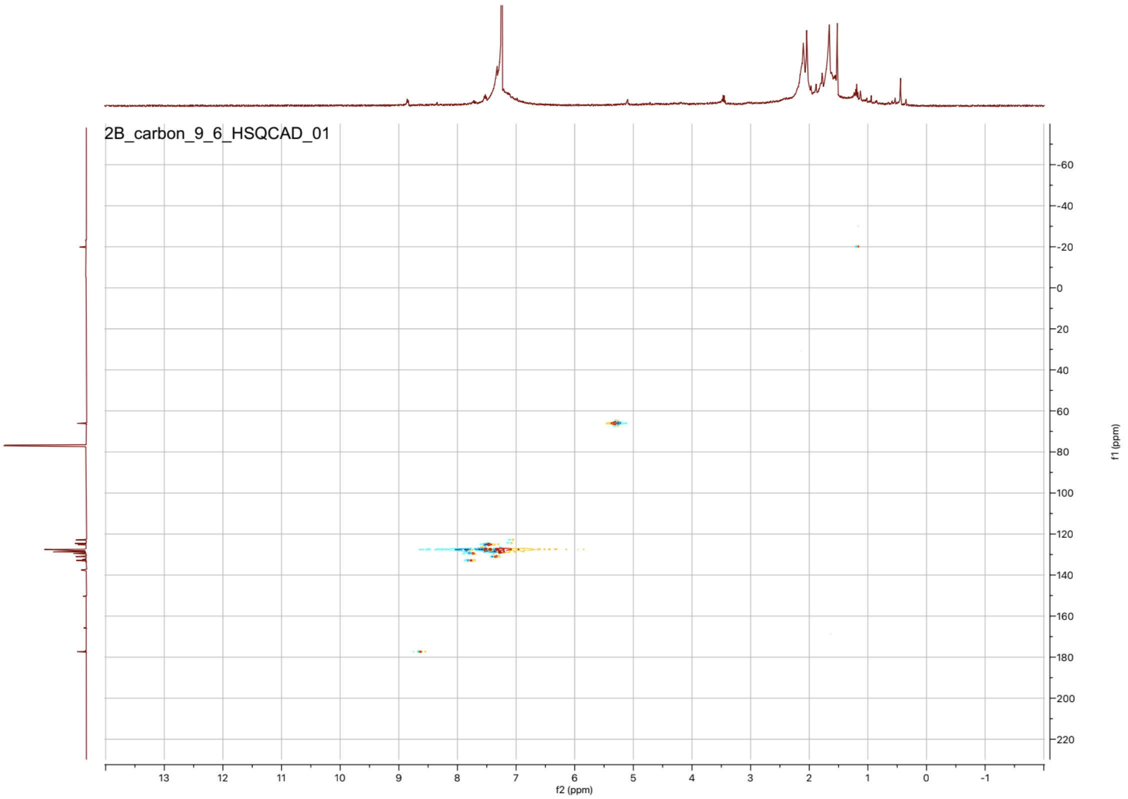

Figure S41. Compound **2B** emission (DCM)

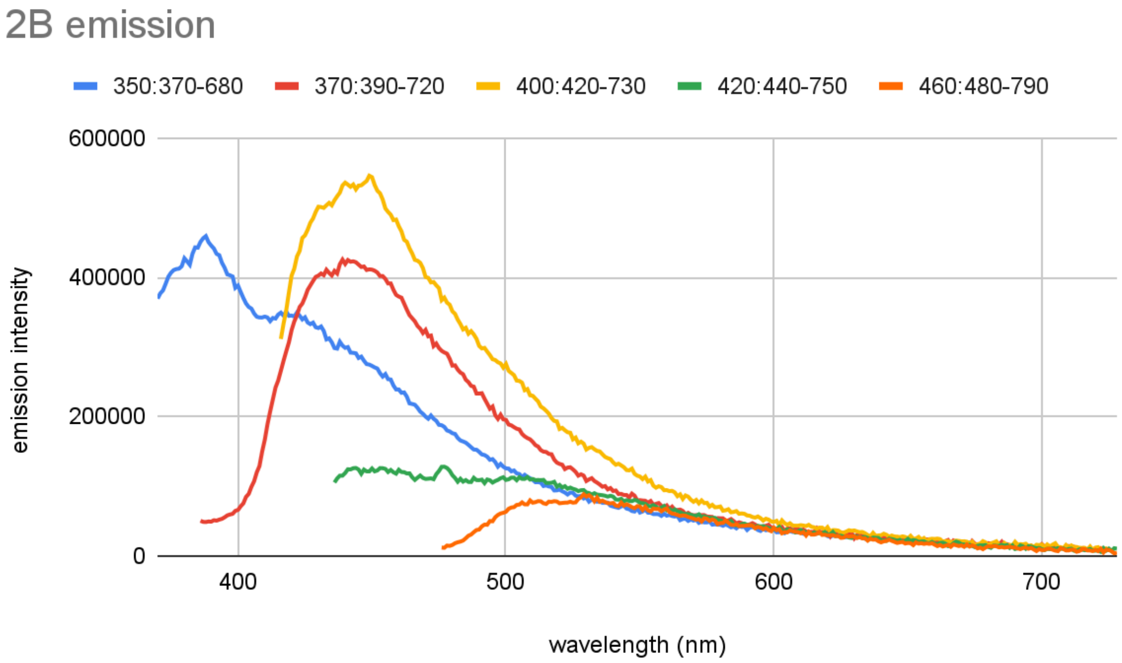

Figure S42. Compound **2B** Uv-vis (DCM)

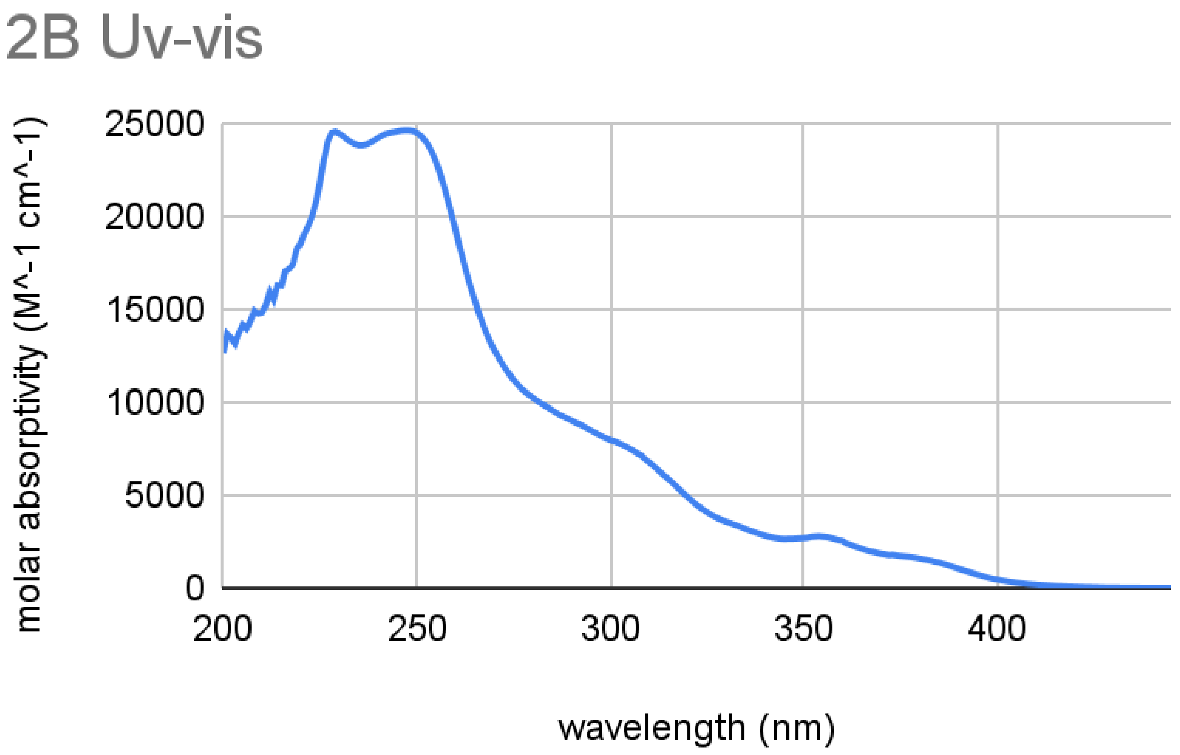

Figure S43. Compound **2B** IR (diamond)

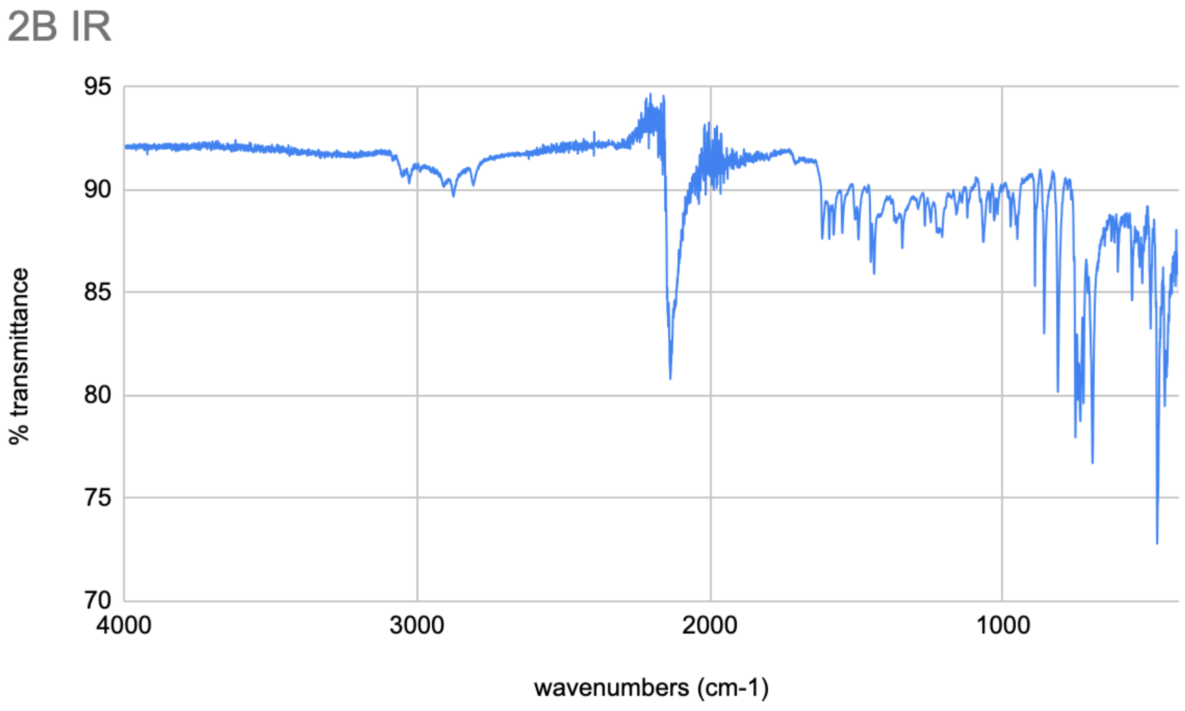

Figure S44. Compound **2B** IR (Ge)

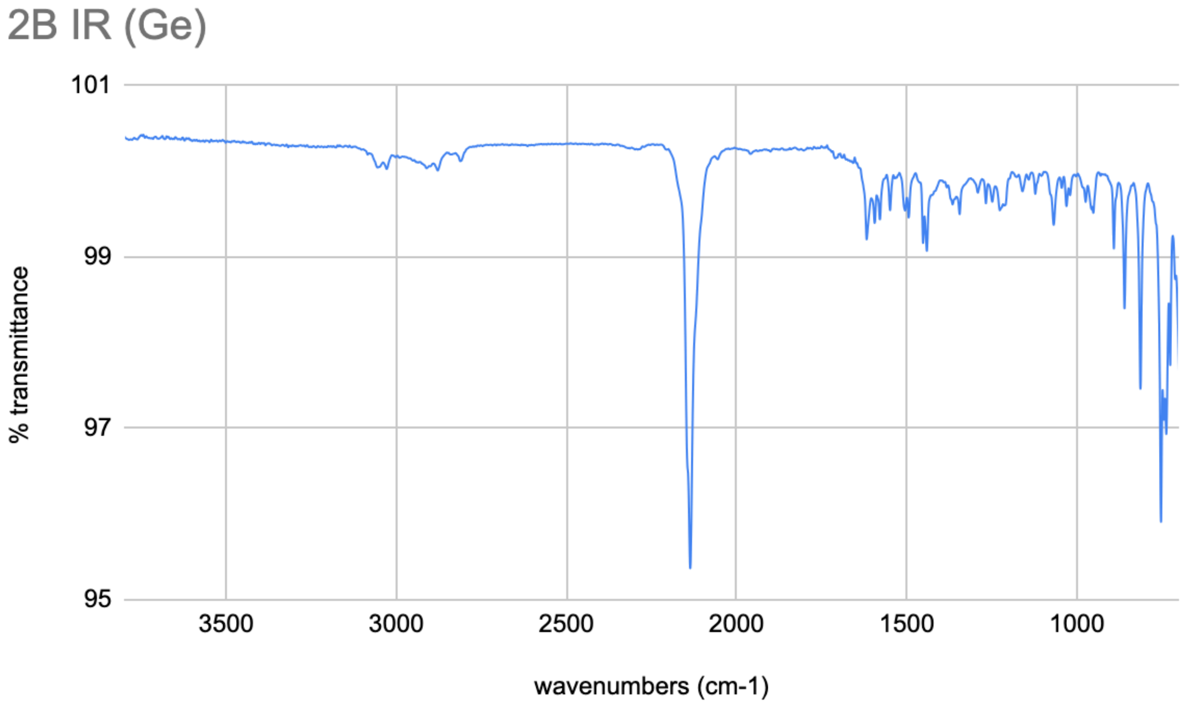

Figure S45. Compound **2C** ^{1}H NMR

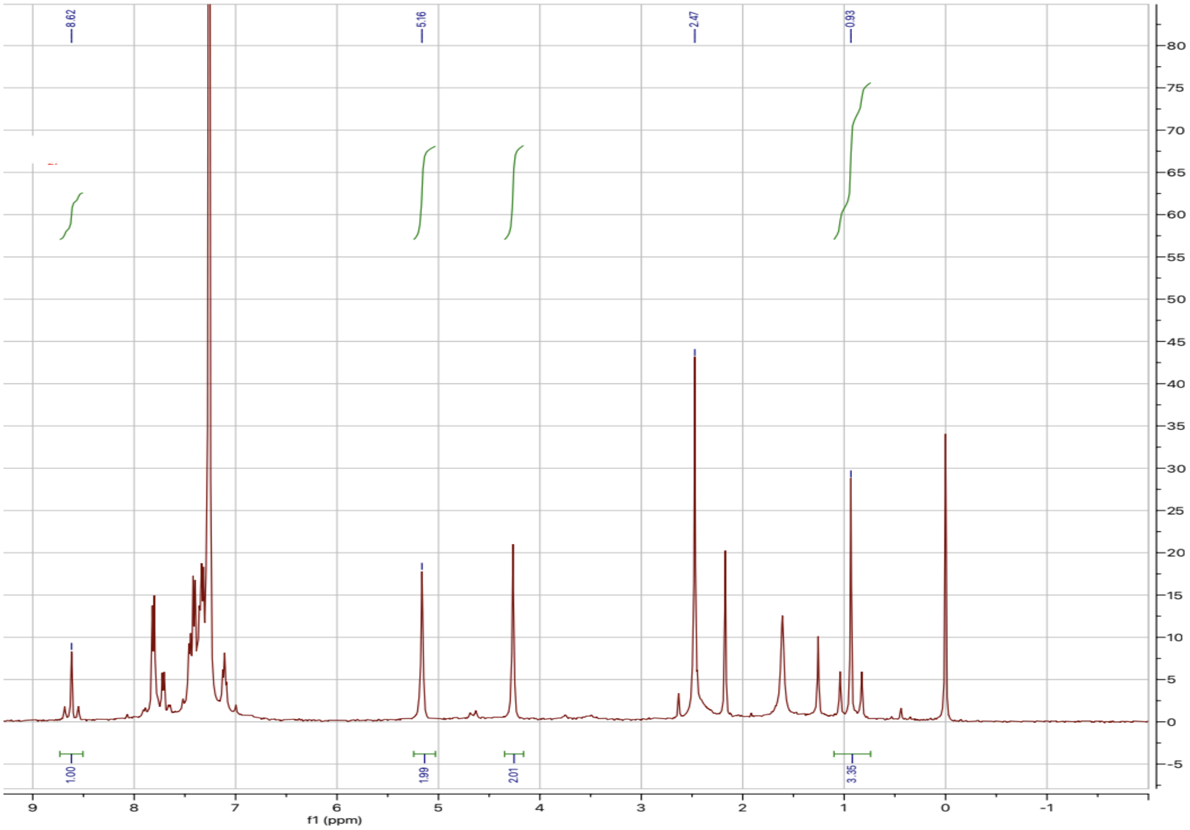

Figure S46. Compound **2C** COSY

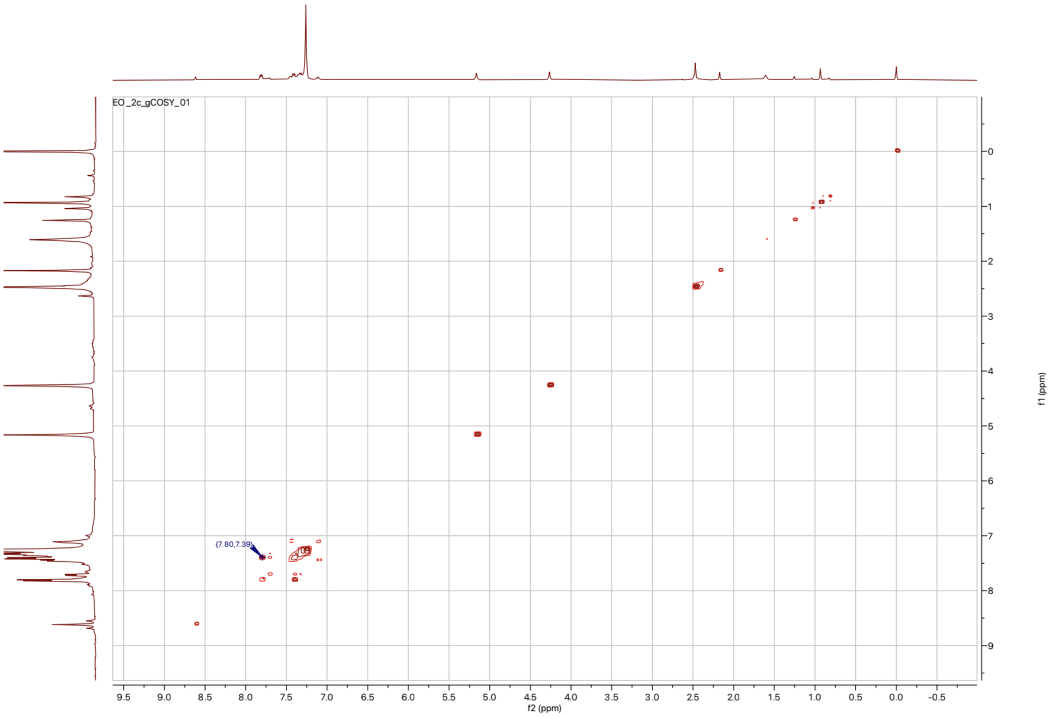

Figure S47. Compound **2C** ^{13}C-NMR

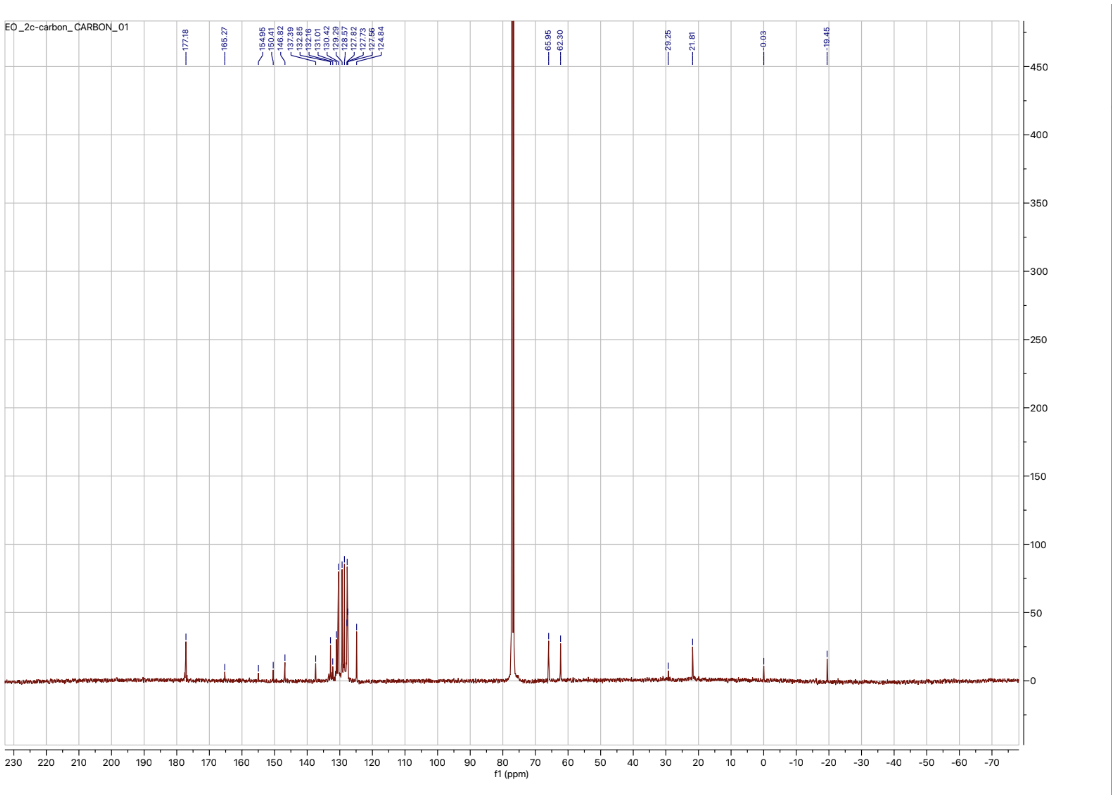

Figure S48. Compound **2C** HSQC

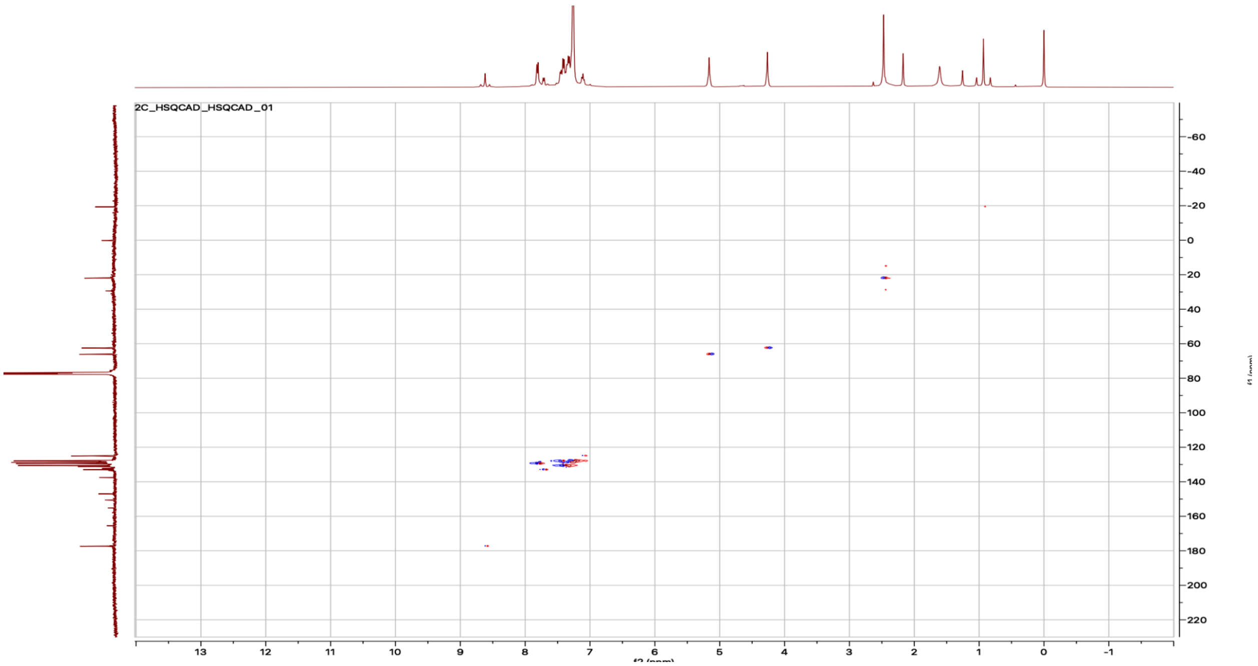

Figure S49. Compound **2C** emission (DCM)

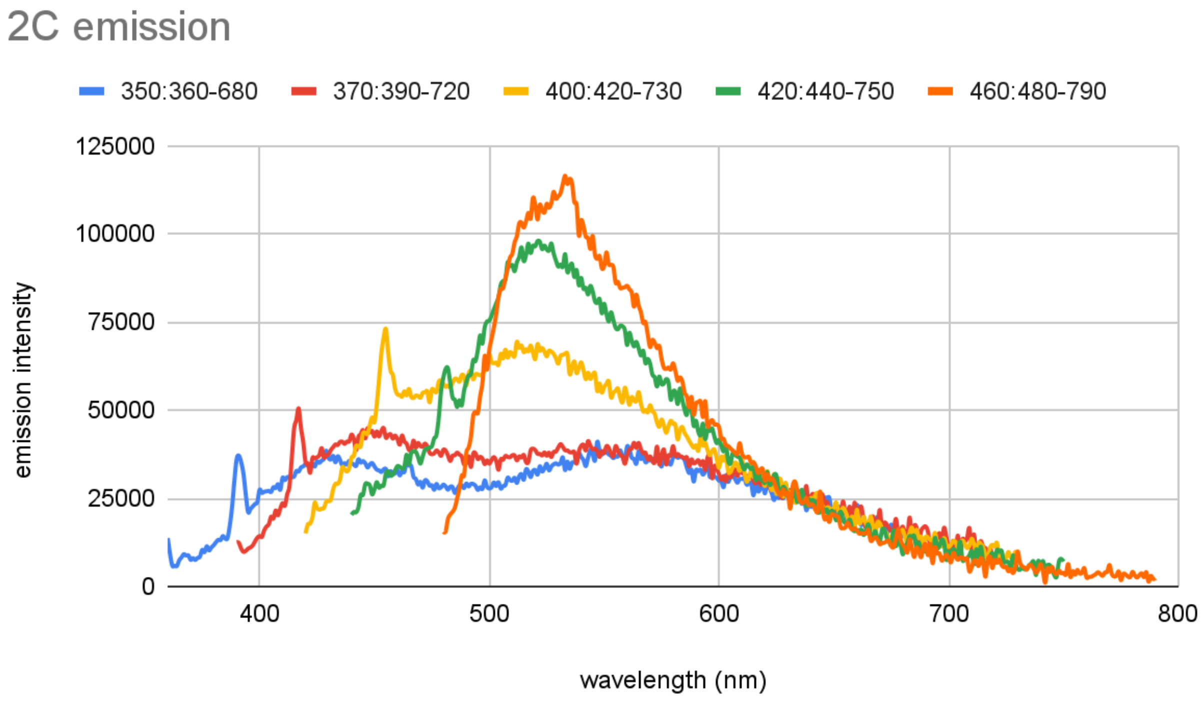

Figure S50. Compound **2C** UV-vis (DCM)

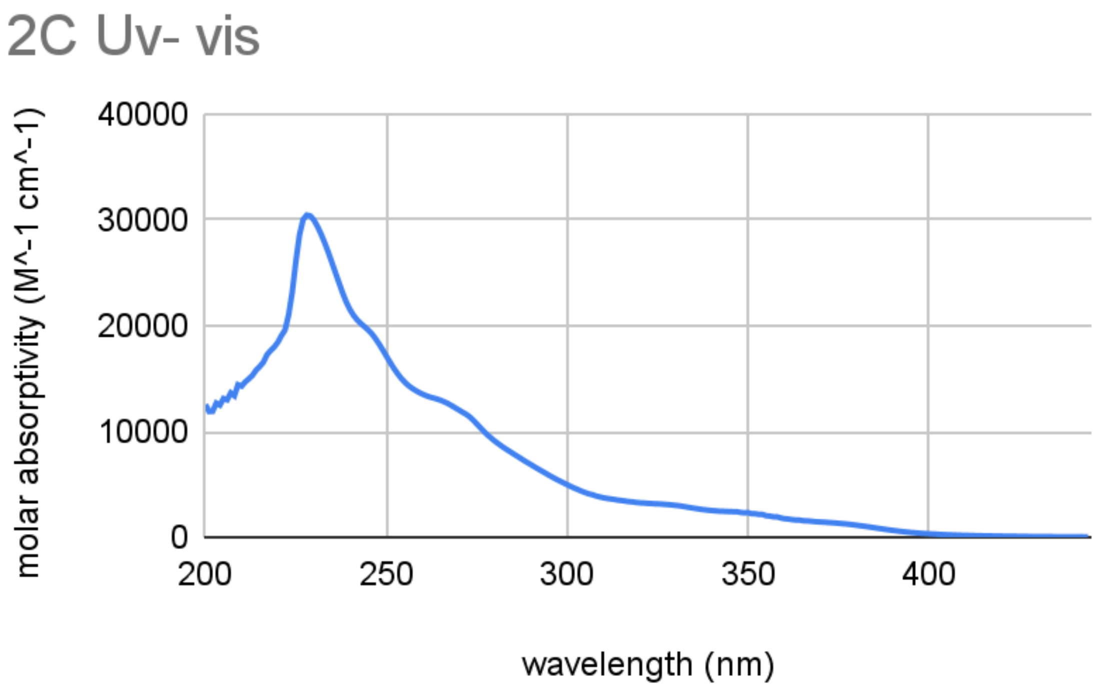

Figure S51. Compound **2C** IR (diamond & Ge)

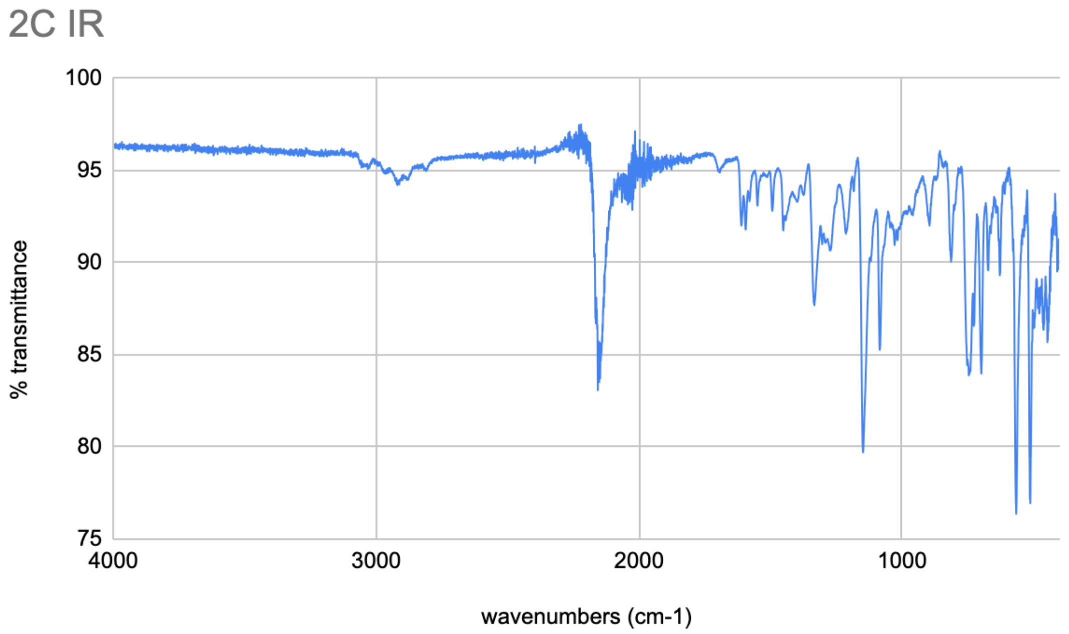

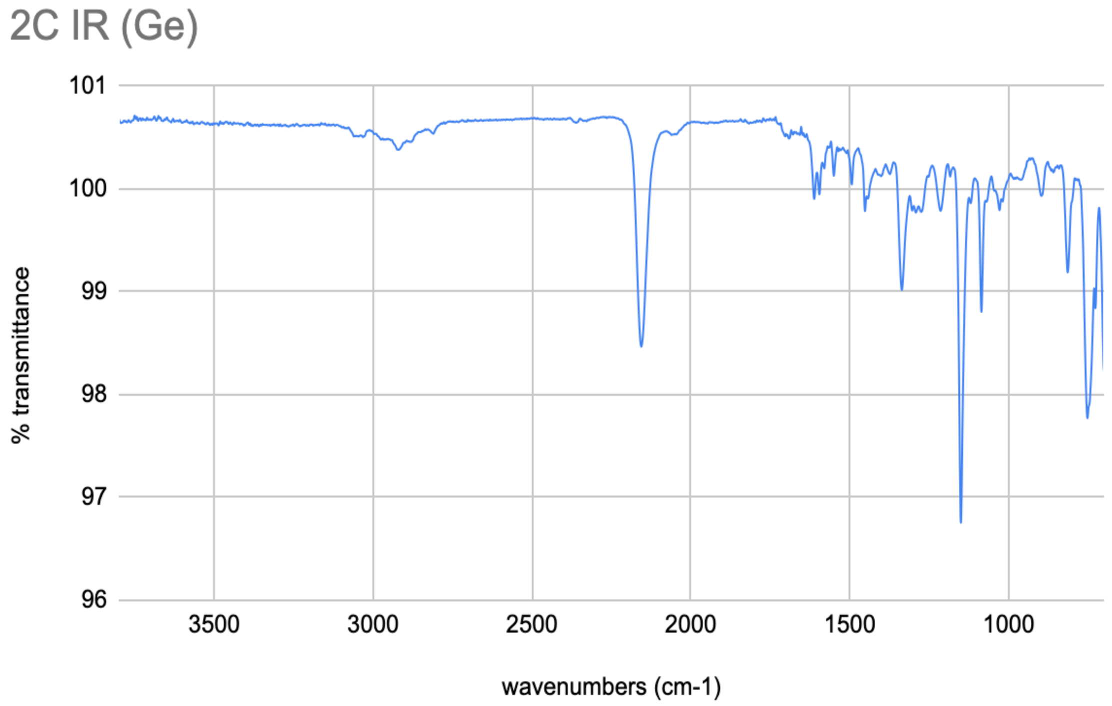

[
Figure S52. Compound **2D** ^{1}H NMR

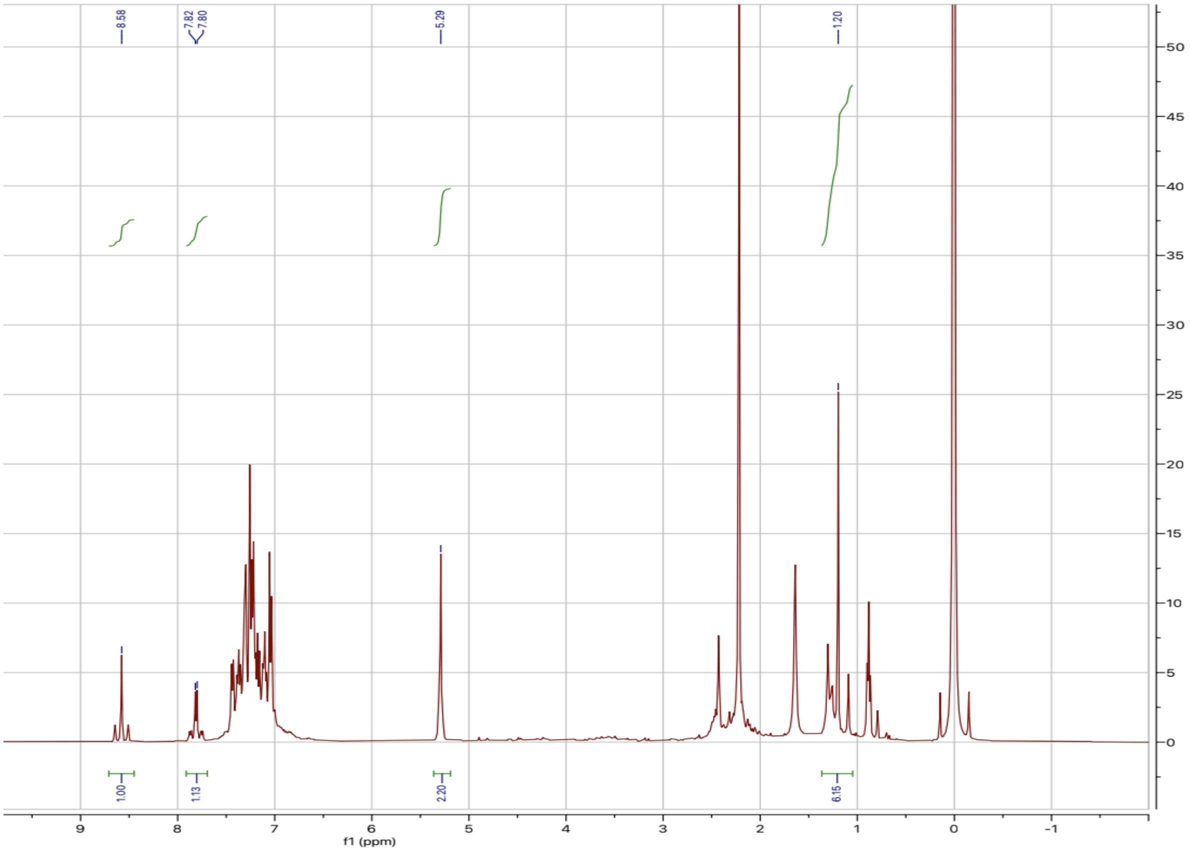

Figure S53. Compound **2D** COSY

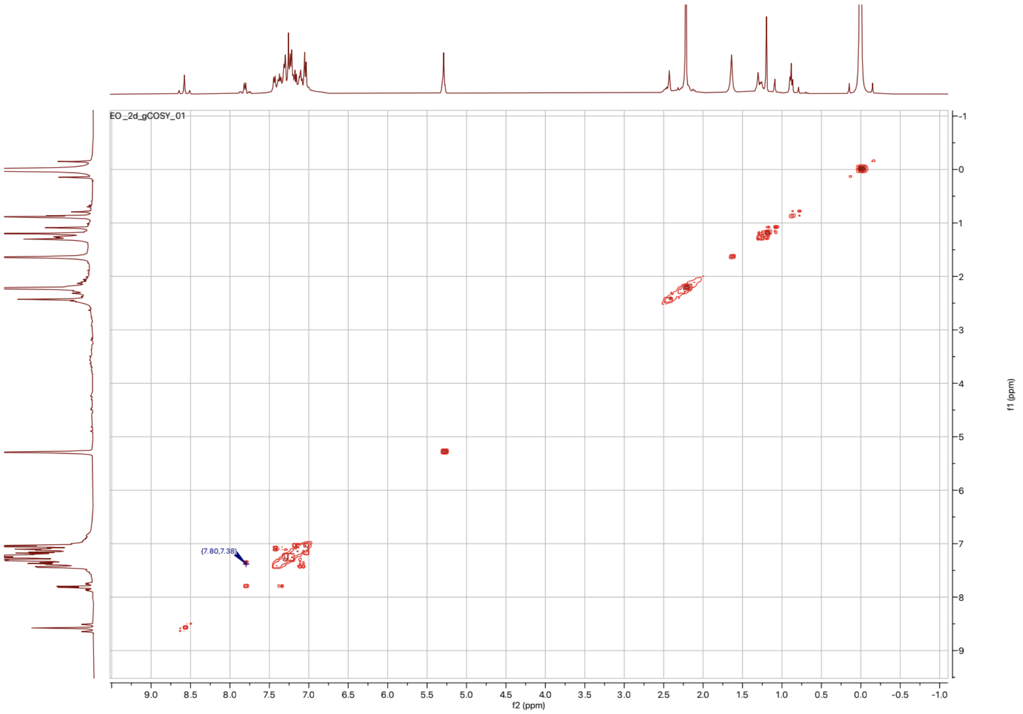

Figure S54. Compound **2D** ^{13}C-NMR

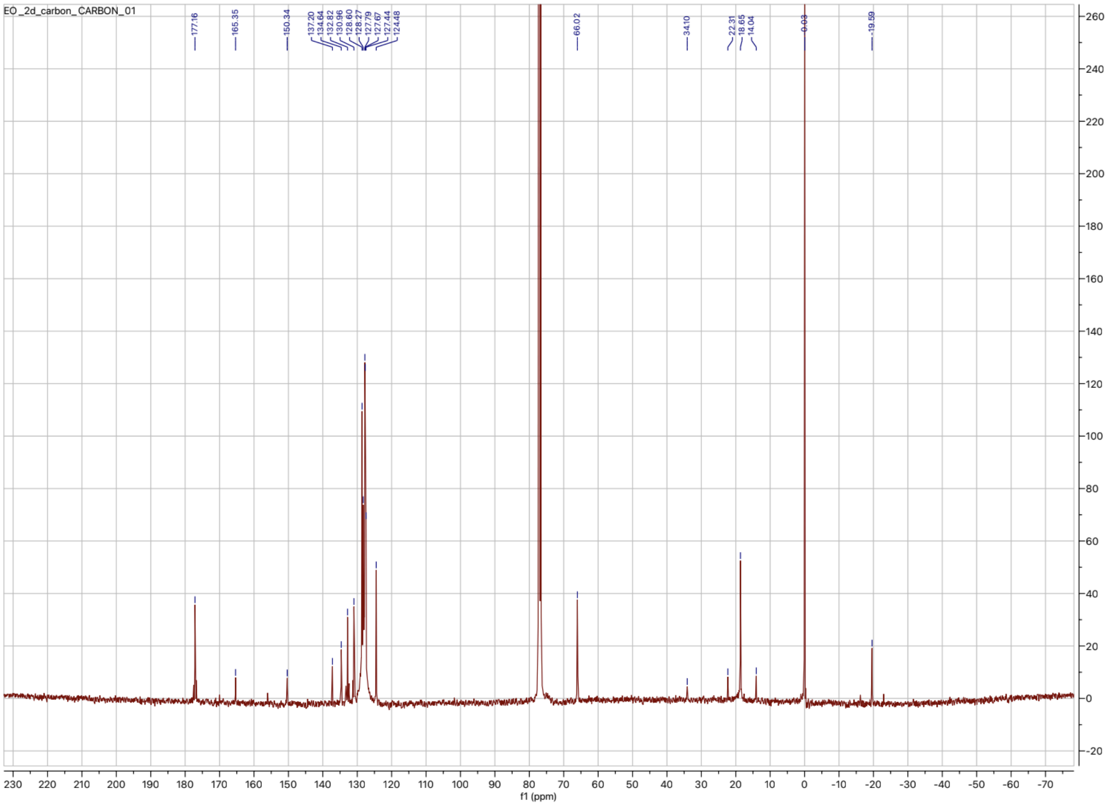

Figure S55. Compound **2D** HSQC

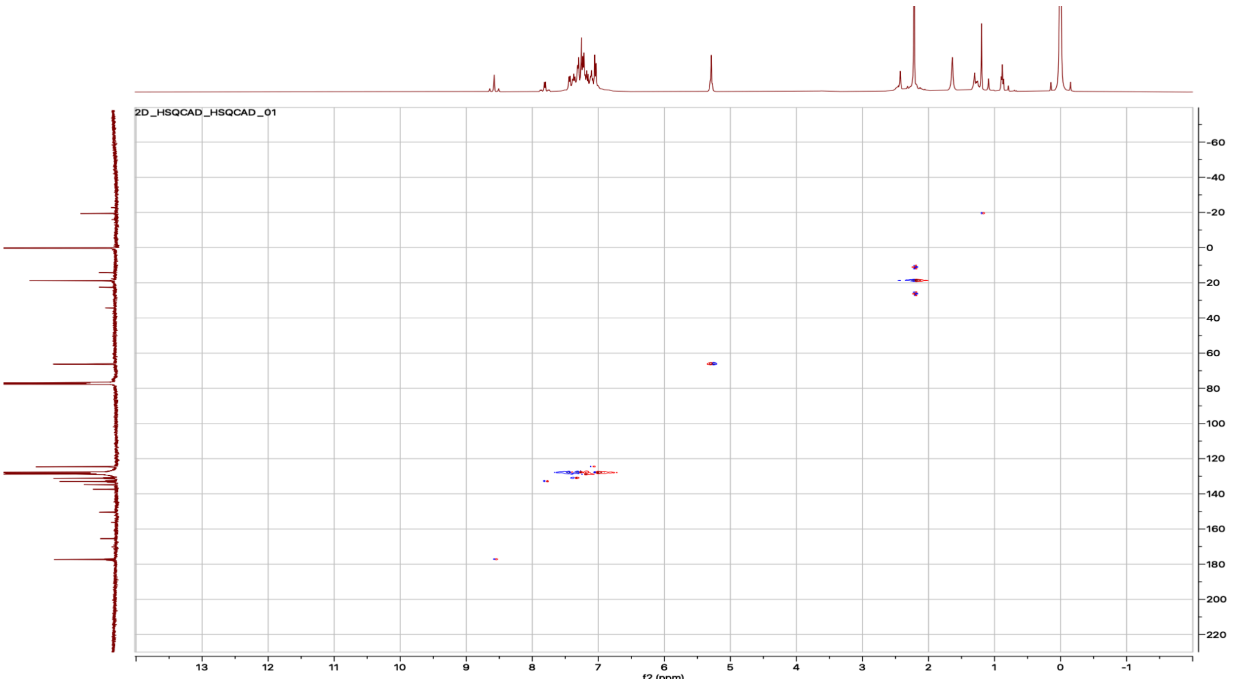

Figure S56. Compound **2D** emission (DCM)

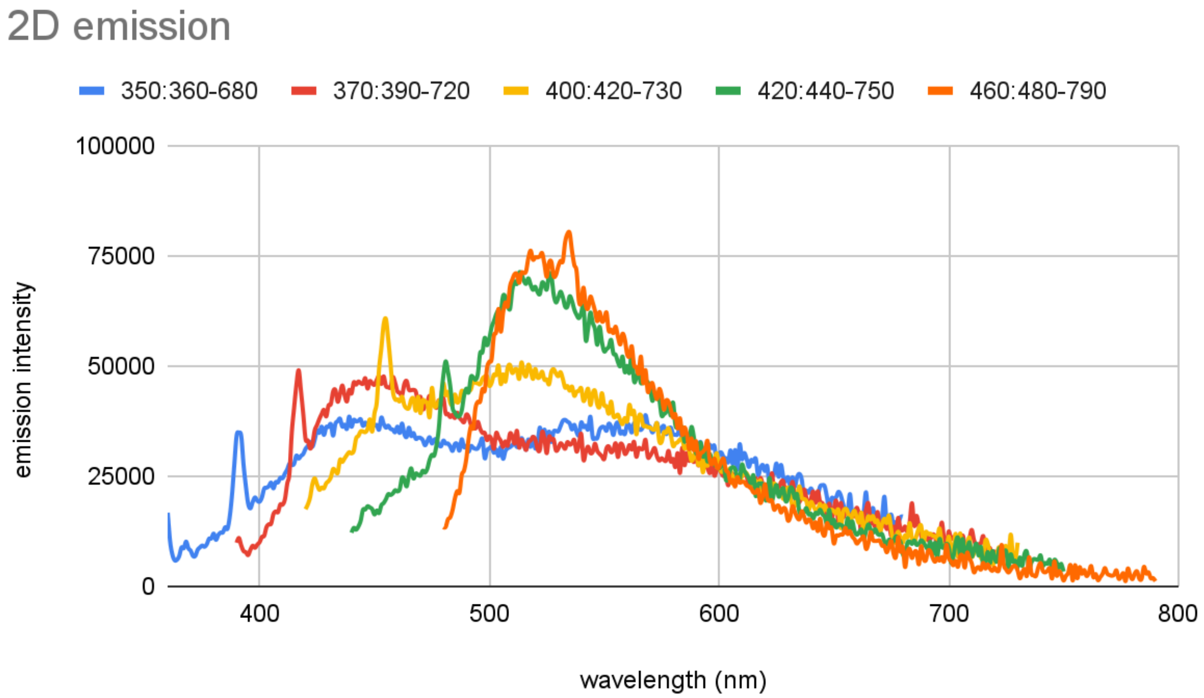

Figure S57. Compound **2D** Uv-vis (DCM)

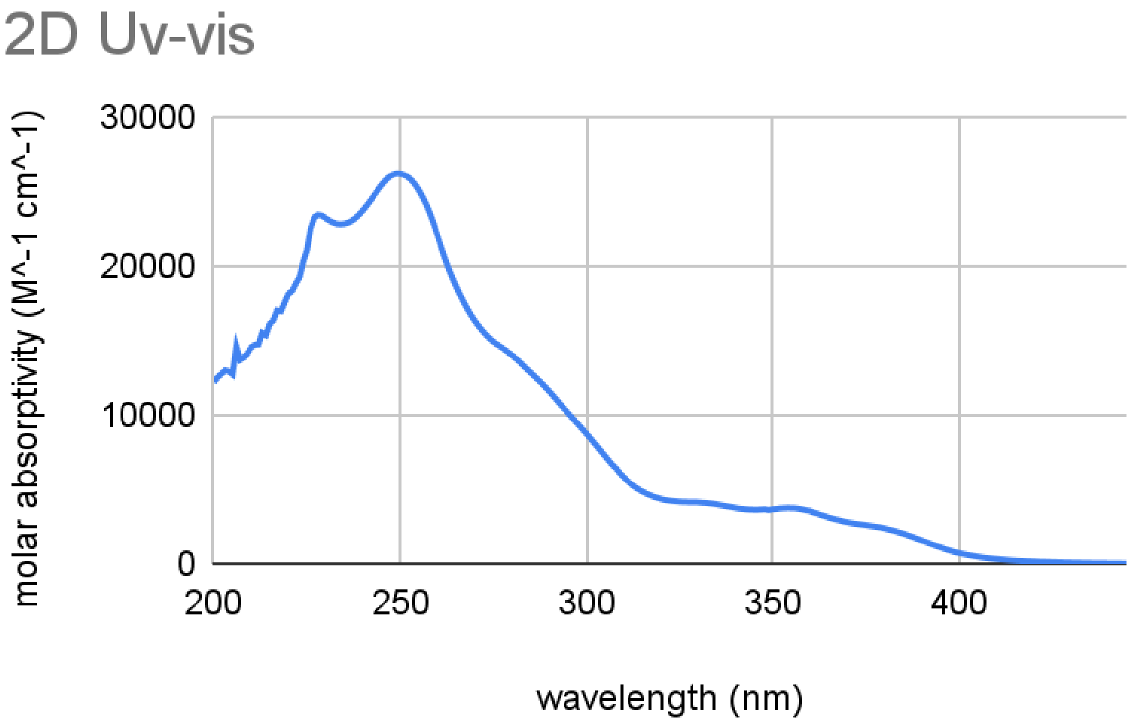

Figure S58. Compound **2D** IR (diamond & Ge)

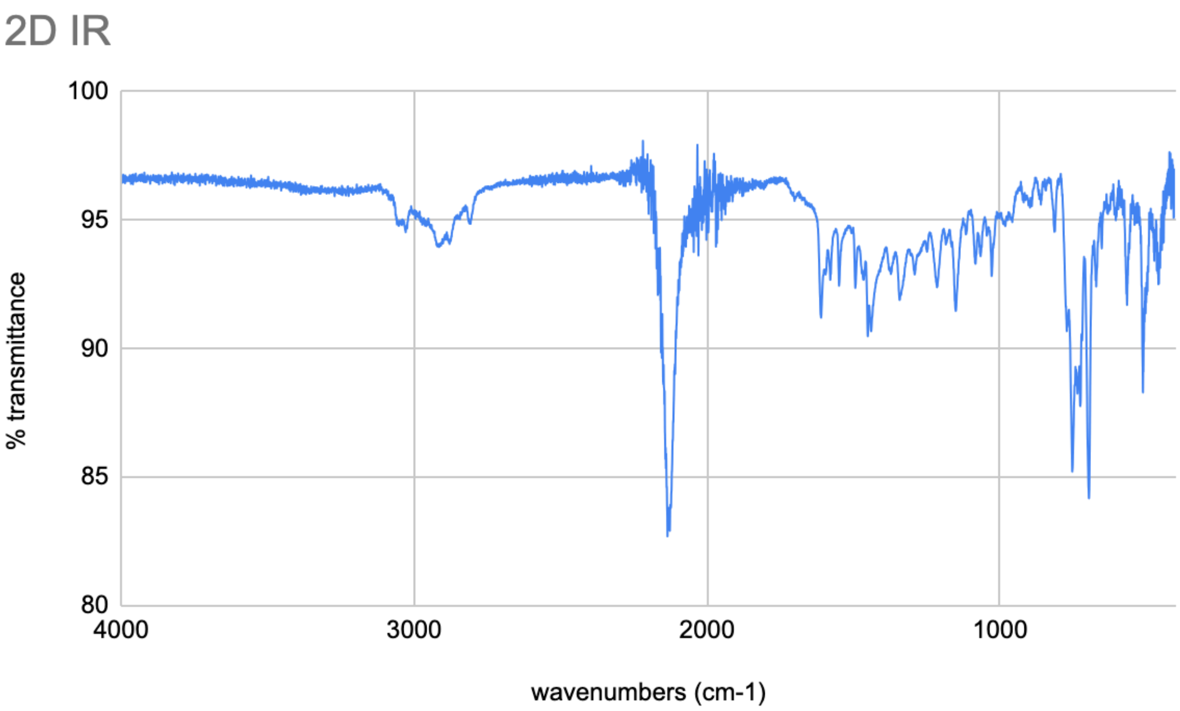

Figure S59. Compound **2D** IR (Ge)

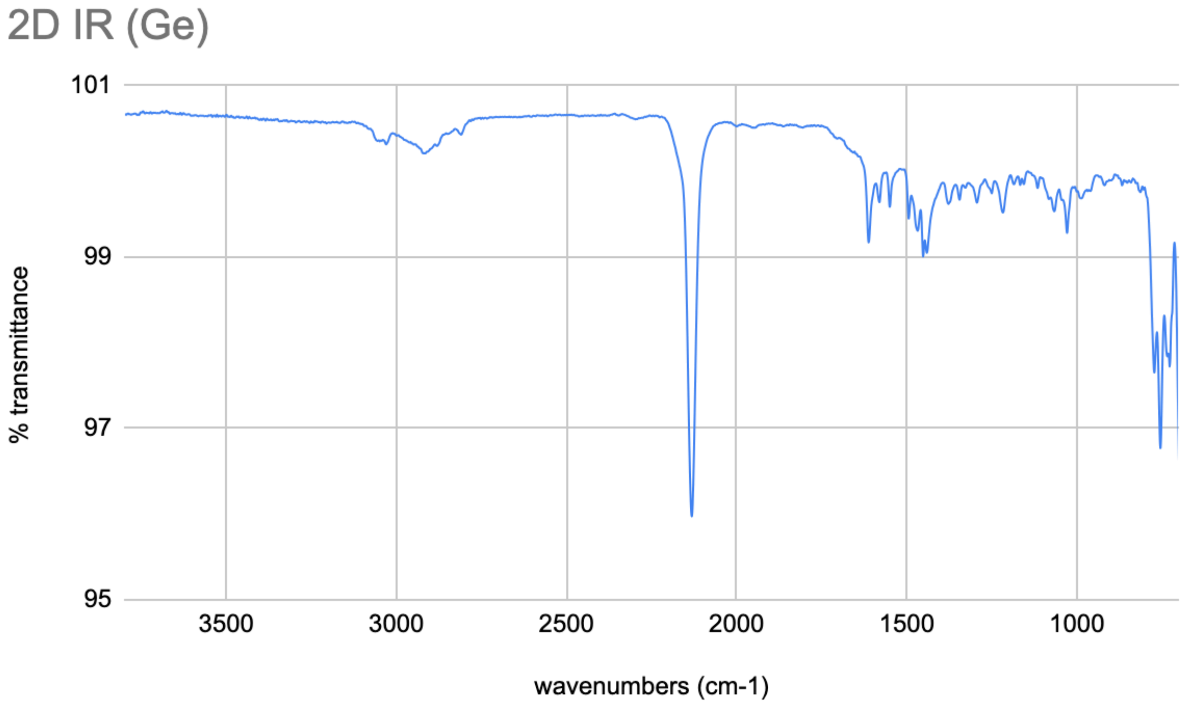

Figure S60. UV-vis spectra for compounds **1A-D** (DCM)

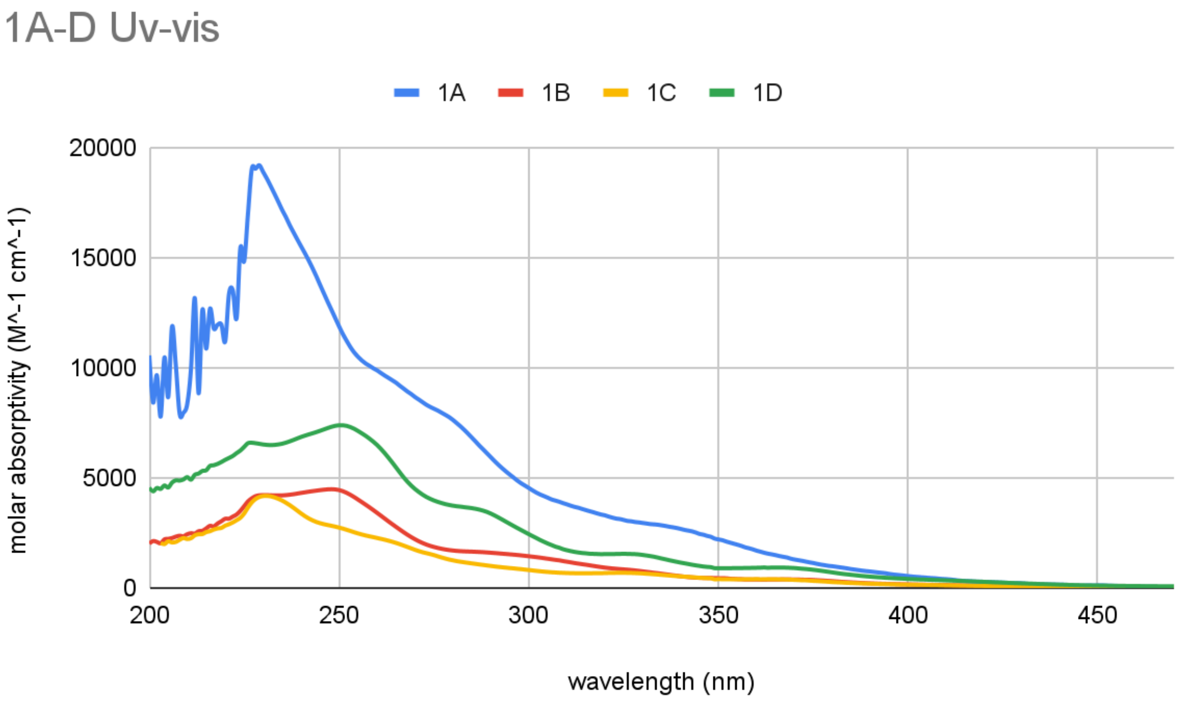

Figure S61. Emission spectra for compounds **1A-D** at 370nm (DCM)

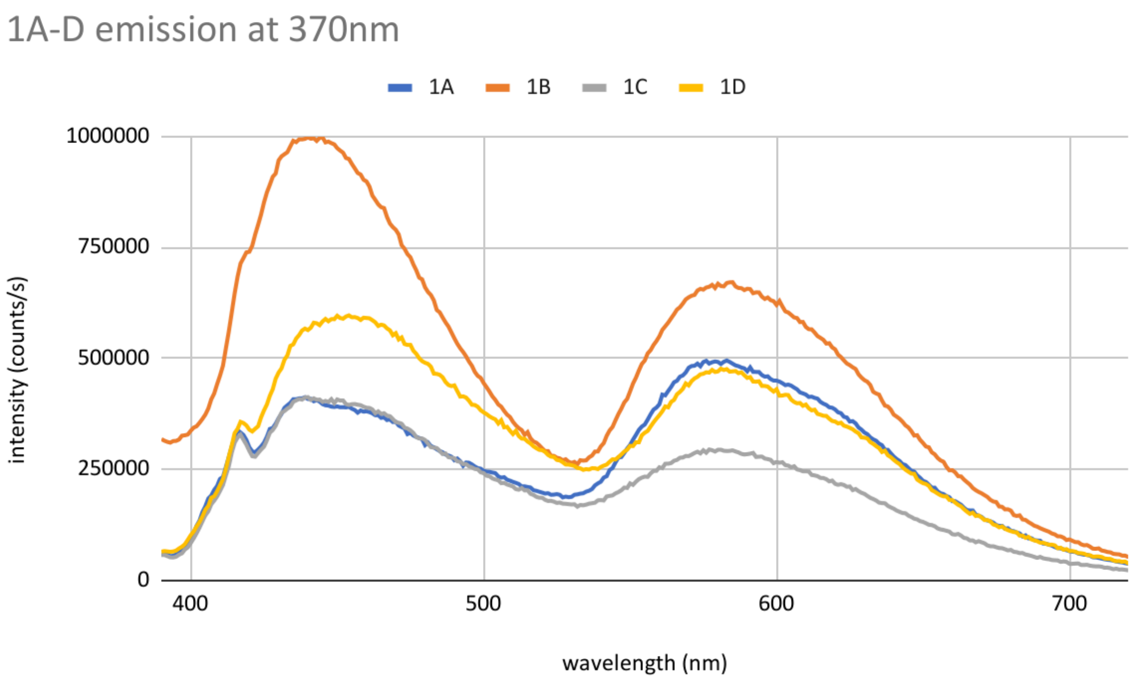

Figure S62. Uv-vis spectra for compounds **2A-D** (DCM)

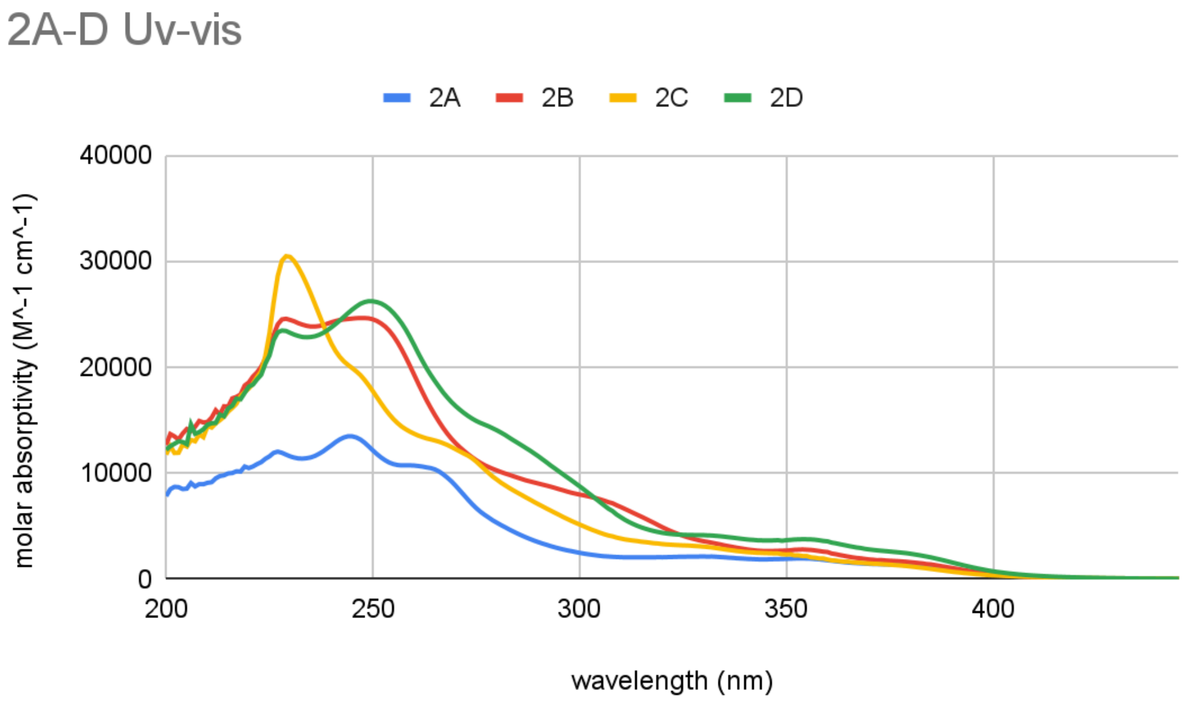

Figure S63. Emission spectra for compounds **2A-D** at 360nm (DCM)

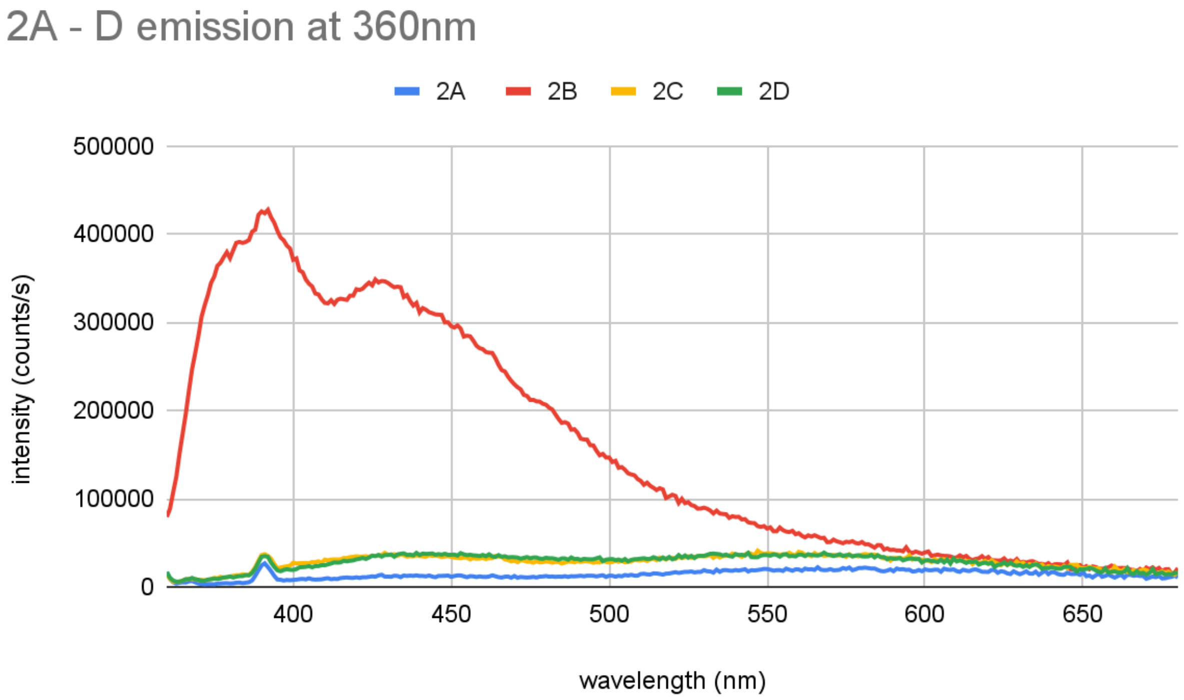

Figure S64. Emission spectra for compounds **2A, 2C, 2D** at 360nm (DCM)

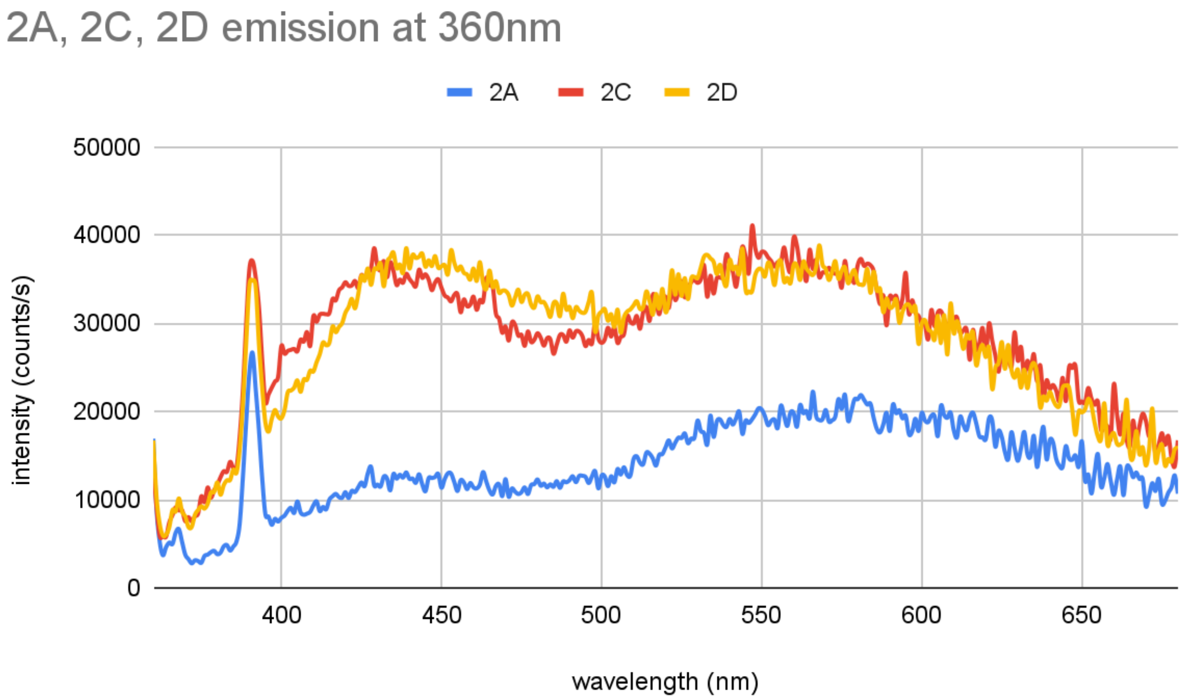

Table S2. Quantum Yields - absolute/integration sphere method.

	1A	1B	1C	1D	2A	2B	2C	2D
Quantum Yield (%) - non-degassed	0.45	1.02	1.15	1.18	0.46	1.80	1.60	1.00
Quantum Yield (%) - degassed	1.24	2.49	1.87	1.49	0.87	1.10	0.41	0.58

Photophysical Measurements

Steady-state emission spectra were recorded by using a PTI QM-400 instrument with a PMT detector, which is sensitive up to 850 nm. In these experiments, the concentration of the platinum complexes ranged from 67-75 μM. The fluorimeter emission spectrum was corrected using a method described in the literature which uses four standard fluorophores to calibrate the response of the instrument.[1] The slits were set at 2.5 nm bandpass for all solution measurements. Solution samples were degassed prior to measurement.

The luminescence lifetimes of the complexes were measured by time-correlated single-photon counting (TCSPC) following excitation with a 365, 405, or 450 nm LED. For TCSPC measurements, the slits were adjusted such that <3% of the LED flashes resulted in a detection event ensuring such events are single photons. Solution samples for lifetime measurements were degassed for 5 min prior to measurement.

Quantum yield measurements were performed as an absolute measurement using a petite integrating sphere (Horiba, *K*-sphere). During quantum yield measurements, the slit sizes were adjusted, and a neutral density filter with an optical density of 1.0 was used to keep the signal in the linear range of the detector (<1 Mcps). Excitation and emission slits were typically set to ~1 nm bandpass. The solvent, DCM, was used as a reference. The integrating sphere was corrected by scaling the solvent emission curve by the ratio of the integrated excitation peaks for the sample and the blank.[2] The accuracy of the absolute measurement using the integrating sphere was checked with $[Ru(bpy)_3]Cl_2$ in water, using the known a quantum yield from the literature of 0.028.[3-5] The measured value of $[Ru(bpy)_3]Cl_2$ in the integrating sphere was 0.035 +/- 0.008 (N=5).

Femtosecond Transient Absorption (fs-TA) spectroscopy was performed at Brookhaven National Laboratory using a Helios Fire (Ultrafast Systems) spectrometer that used a Spitfire Spectra
Physics regenerative amplifier and two TOPAS OPAs operating at 1 kHz repetition rate. fs-TA data reduction and analysis was performed using Surface Xplorer 4.3.0 software (Ultrafast Systems). Raw data were background subtracted and chirp corrected. Singular value decomposition was used to determine the number of principal components. Multi-exponential global analysis fitting was then performed to extract decay associated difference spectra and their corresponding lifetimes.

Table S3. Excited State Lifetimes (150 ns range, degassed)

	Excitation LED (nm)	Emission Wavelength (nm)	χ^2	τ (ns)	Range Fitted (ns)
**1A**	365	445	1.07	2.2	29.64-38.88
	365	580	1.074	3.0, 58	28.91-133.5
	365	620	1.048	4.1, 139	32.13-134.00
**1B**	365	415	1.197	2.9, 8.8	28.91-102.9
	365	580	1.011	3.6, 52	28.17-131.8
	365	620	1.082	2.6, 76.5	28.91-134.3
**1C**	365	580	1.167	8.3	36.03-92.43
	365	620	1.176	8.5	33.04-131.6
**1D**	405	585	1.185	2.2, 8.4	32.97-133.4
	405	620	1.174	4.1, 30	34.65-134.4
**2A**	365	440	1.048	1.7, 9.1	32.43-62.8
	365	560	1.376	1.6, 7.7	31.11-114.2
**2B**	365	460	1.081	3.0, 11.8	30.45-131.4
	365	530	1.031	3.6, 15.2	31.15-133.8
	365	550	1.174	3.5, 16.9	29.50-132.9
	405	460	0.9945	3.3	35.65-50.17
	405	500	1.13	3.7	35.55-49.88
	405	530	1.269	4.7	35.94-42.43
	405	550	1.176	3.4, 9.3	35.41-133.2
	450	530	1.053	1.6, 7.2	30.11-131.11
	450	550	0.9966	1.6, 7.3	29.93-133.0
	450	580	0.9849	1.6, 6.7	29.49-124.3
	365	440	1.220	2.7, 11.4	32.73-98.26

**2C**	365	560	1.177	3.5, 20.1	30.04-99.04
**2D**	365	440	1.182	2.1, 10.7	29.50-101.4
	365	560	1.195	1.6, 9.7	29.42-102.7

Table S4. Photophysical data

**Compound**	**Absorption / Extinction Coefficient (nm)/ ε ($M^{-1}cm^{-1}$) x (10^3)**	**Emission in degassed DCM (nm)**	**Excitation Wavelength (nm)**	**Stokes Shift (($\Delta\lambda_{emsmax}$-λ_{ex})) (nm)**
**1A**	325 (1.9)<br>370 (1.0)<br>410 (0.4)	440, 580	370	70, 210
**1B**	300 (1.4)<br>330 (1.6)<br>350 (1.7)<br>370 (1.8)<br>410 (0.13)	440, 580	370	70, 210
**1C**	330 (1.4)<br>370 (1.5)<br>410 (.12)	450, 580	370	60, 210
**1D**	330 (1.5)<br>370 (1.7)<br>410 (0.12)	450, 580	370	60, 210
**2A**	330 (2.1)<br>355 (2.0)<br>380 (1.3)	450, 580	370	80, 210
		530	460	70
**2B**	300 (1.3)<br>355 (1.5)<br>380 (1.6)	450	370	80
		530	460	70
**2C**	330 (2.2)<br>350 (1.6)<br>380 (1.2)	430, 560	350	80, 210
		530	460	70
**2D**	330 (3.5)<br>355 (3.6)<br>380 (2.4)	440, 560	350	90, 210
		520	460	80

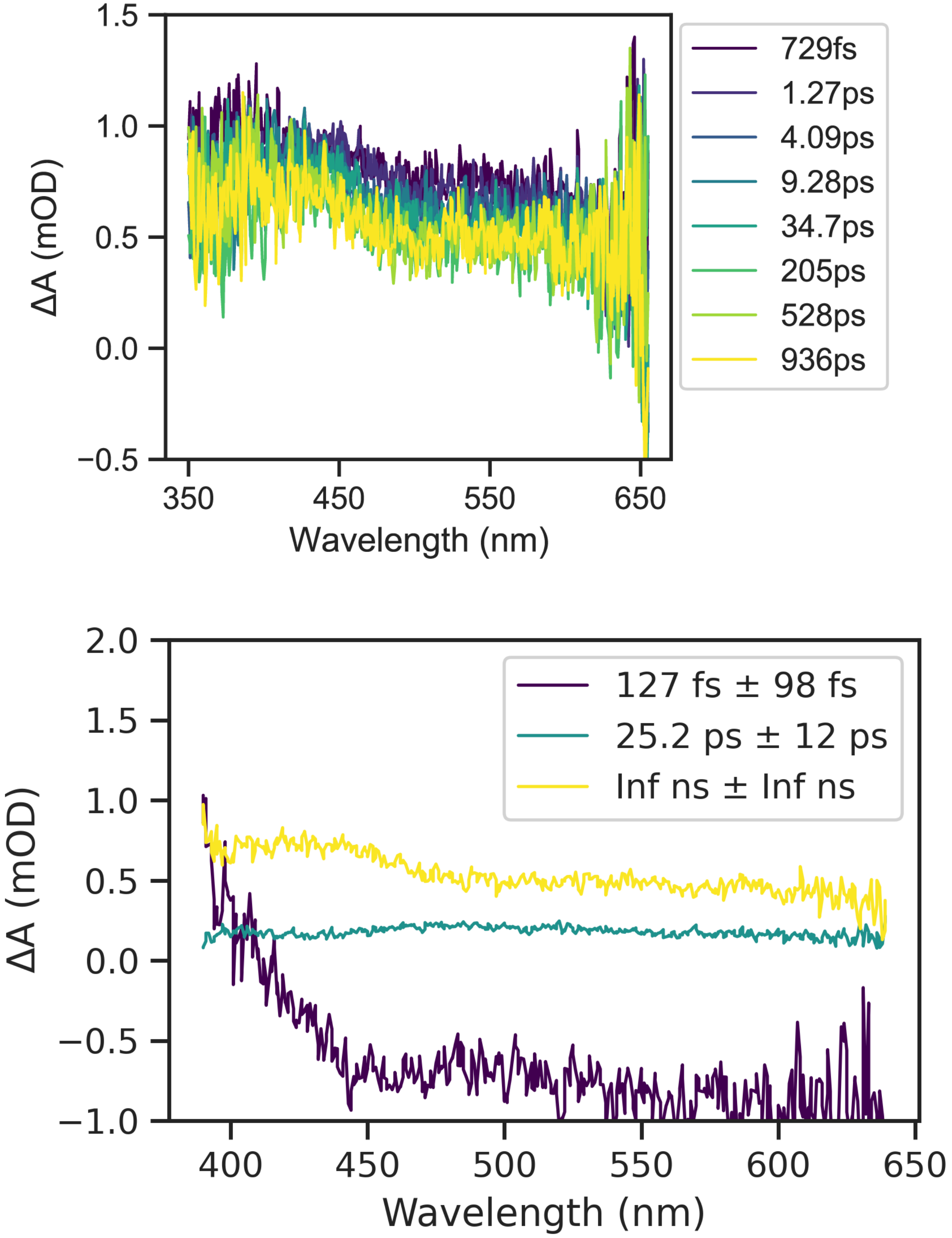

Figure S65 1A fs-TA data. Representative spectra (top) and decay-associated difference spectra (bottom) fit with three principle components.

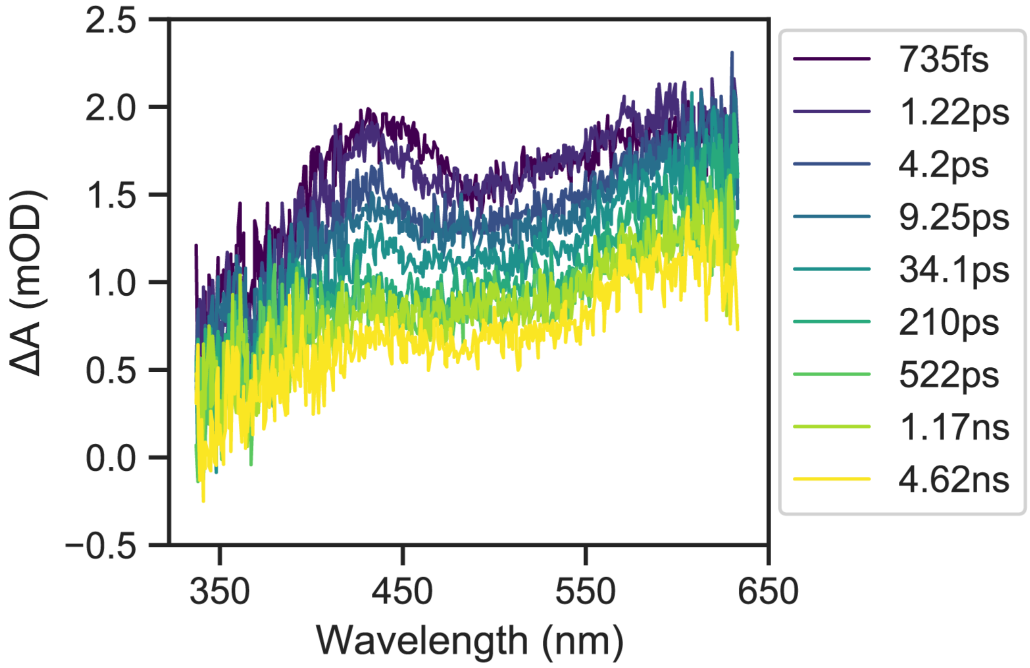

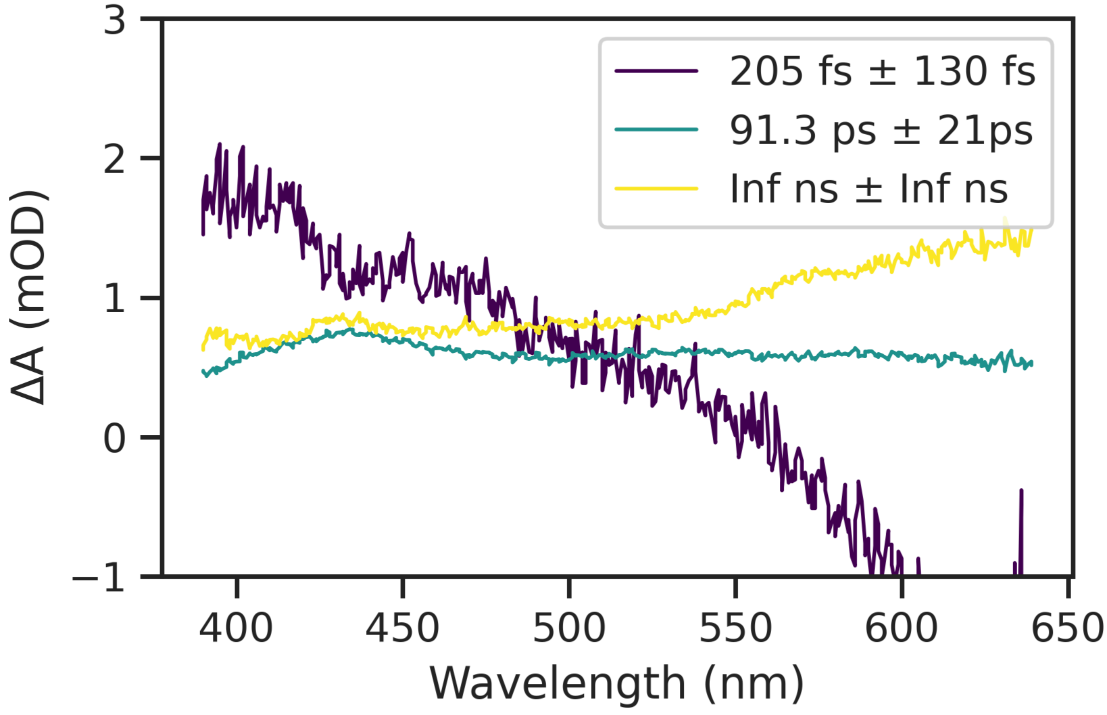

Figure S66 1B fs-TA data. Representative spectra (top) and decay-associated difference spectra (bottom) fit with three principle components.

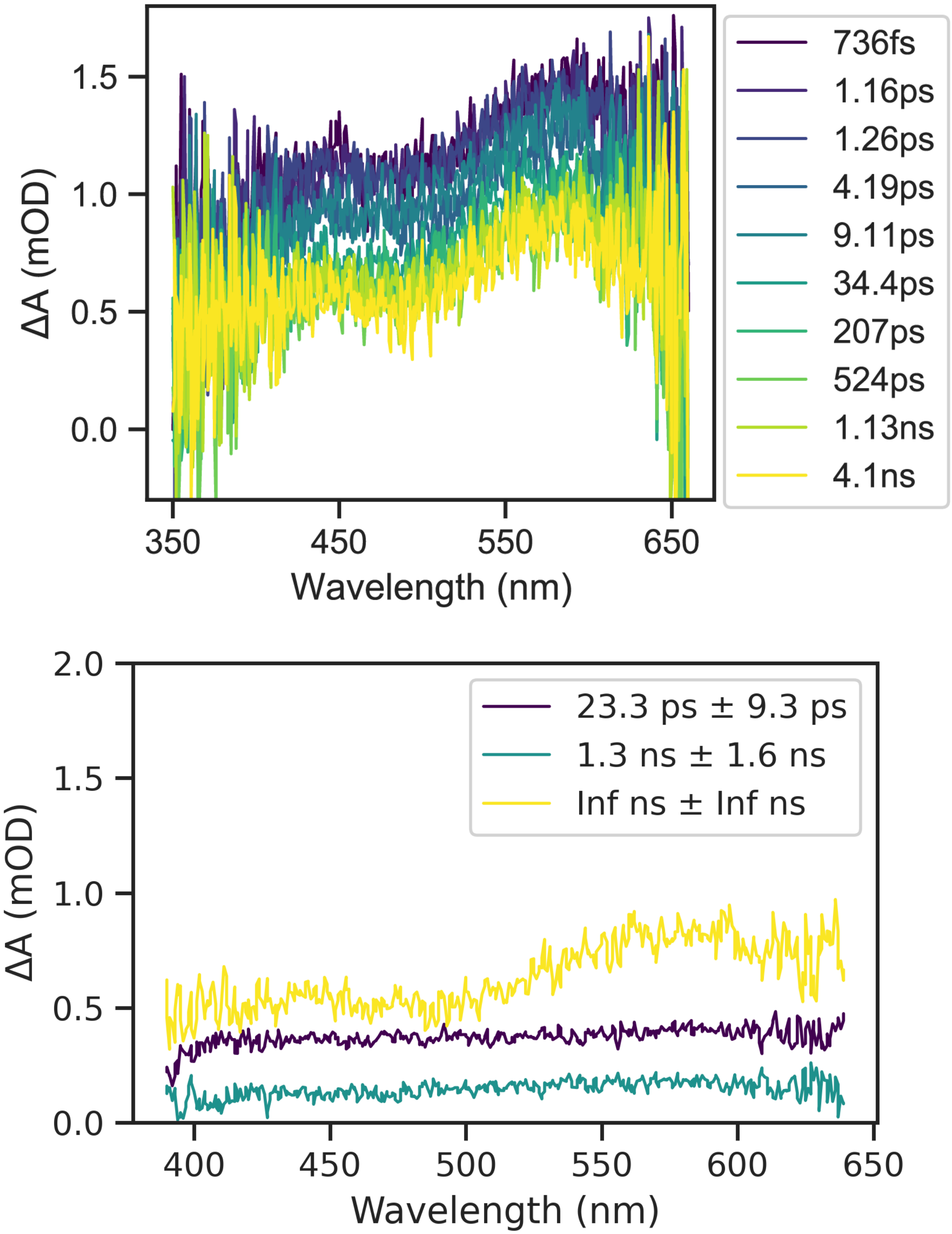

Figure S67 1C fs-TA data. Representative spectra (top) and decay-associated difference spectra (bottom) fit with three principle components.

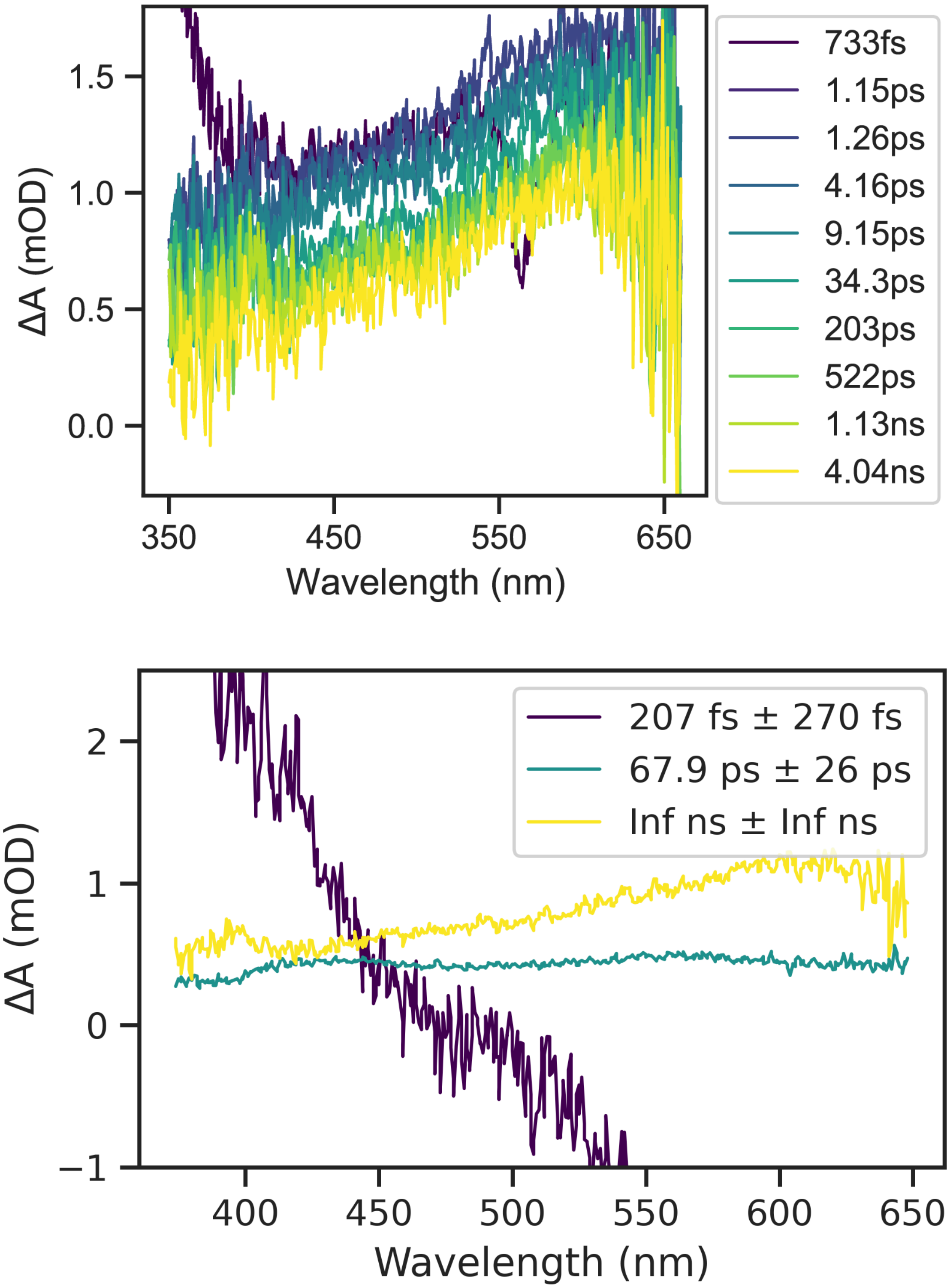

Figure S68 1D fs-TA data. Representative spectra (top) and decay-associated difference spectra (bottom) fit with three principle components.

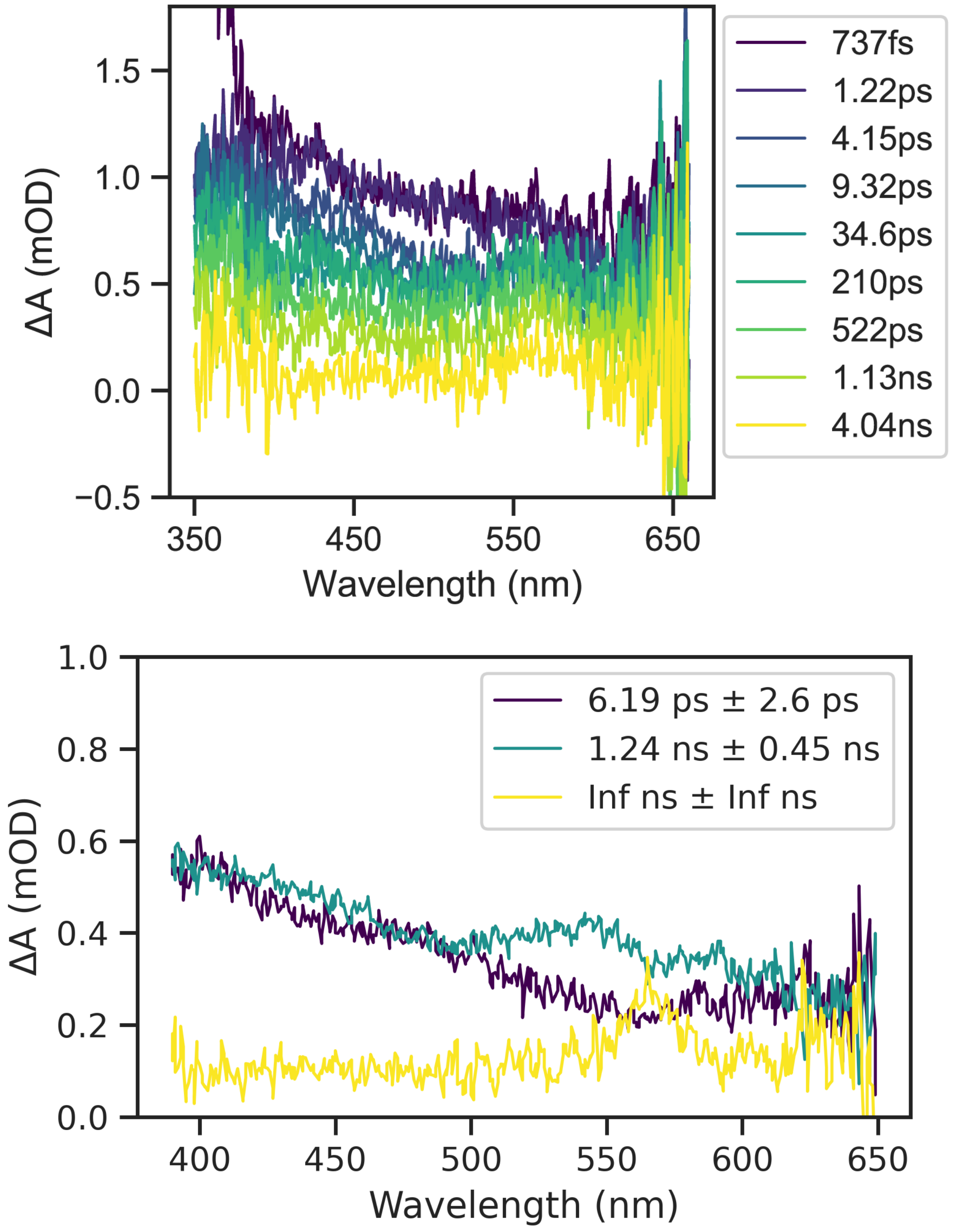

Figure S69 2A fs-TA data. Representative spectra (top) and decay-associated difference spectra (bottom) fit with three principle components.

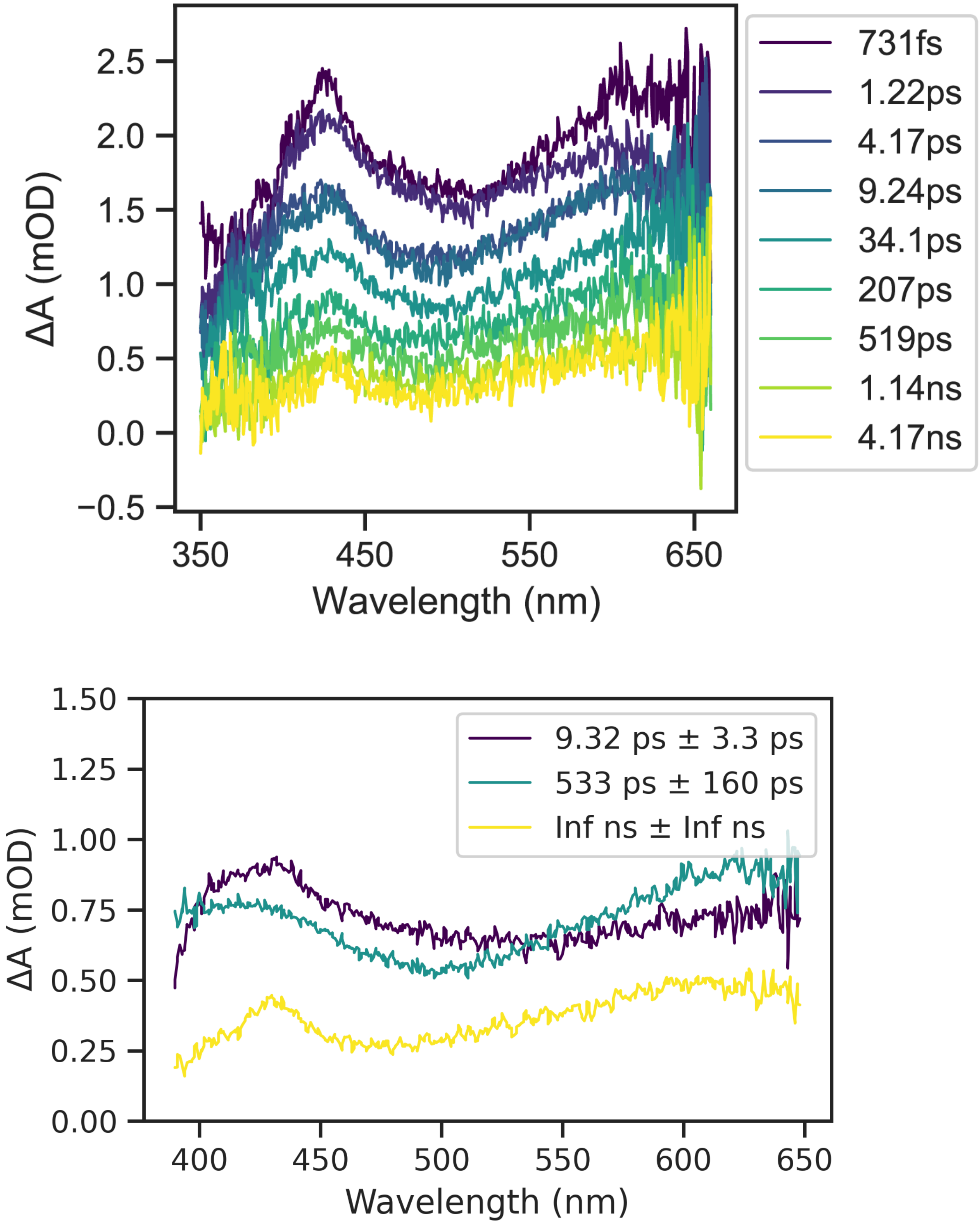

Figure S70 2B fs-TA data. Representative spectra (top) and decay-associated difference spectra (bottom) fit with three principle components.

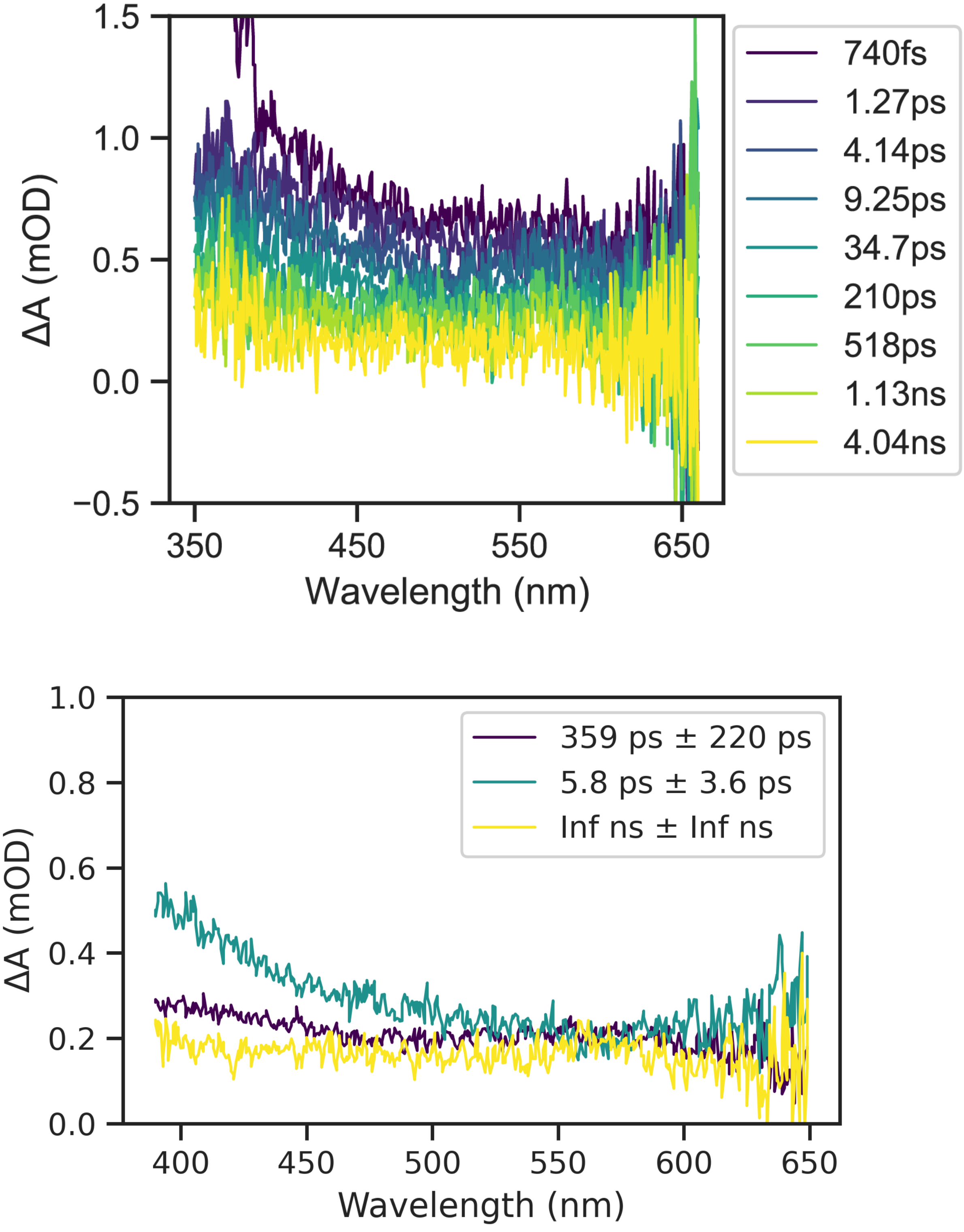

Figure S71 2C fs-TA data. Representative spectra (top) and decay-associated difference spectra (bottom) fit with three principle components.

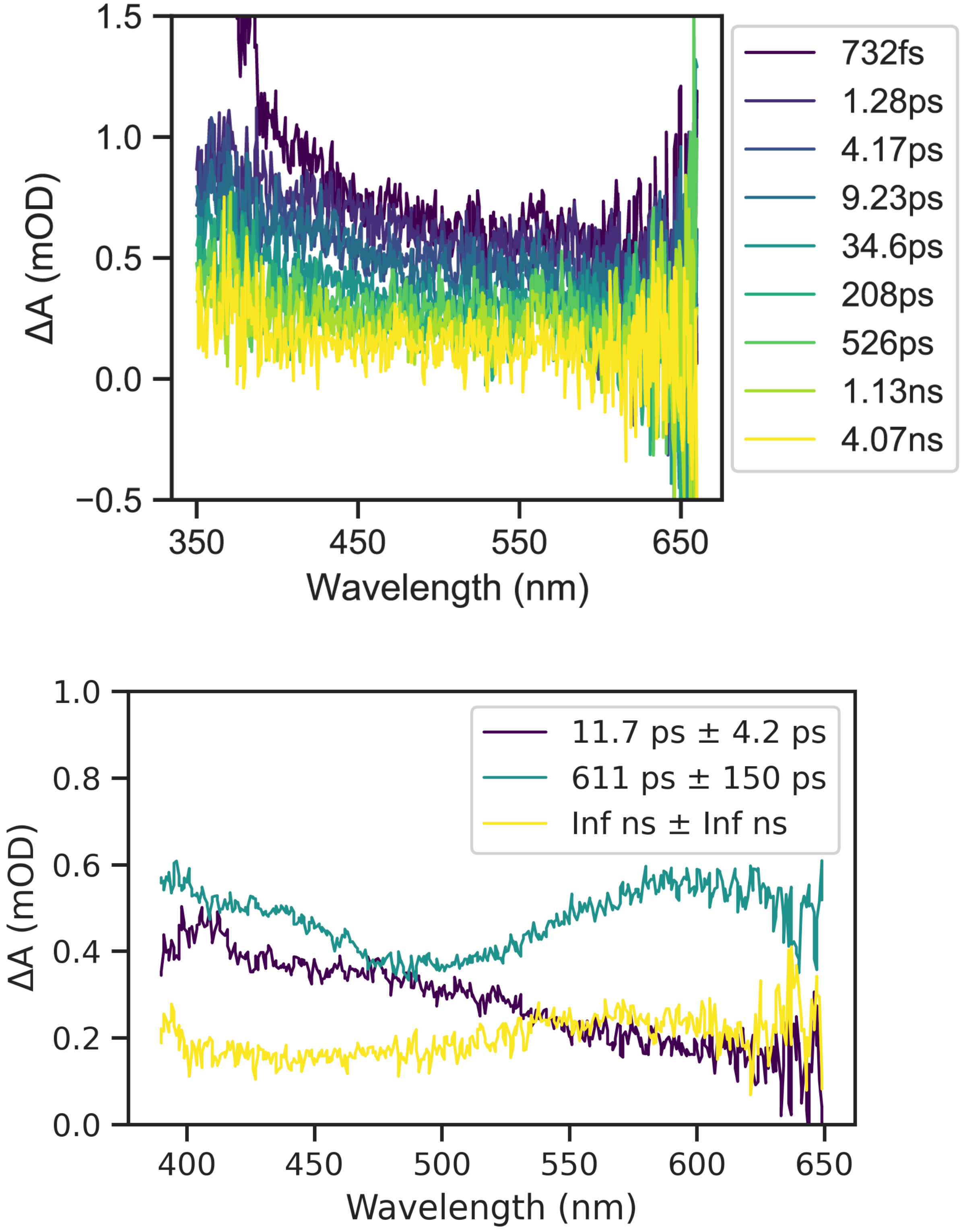

Figure S72 2D fs-TA data. Representative spectra (top) and decay-associated difference spectra (bottom) fit with three principle components.

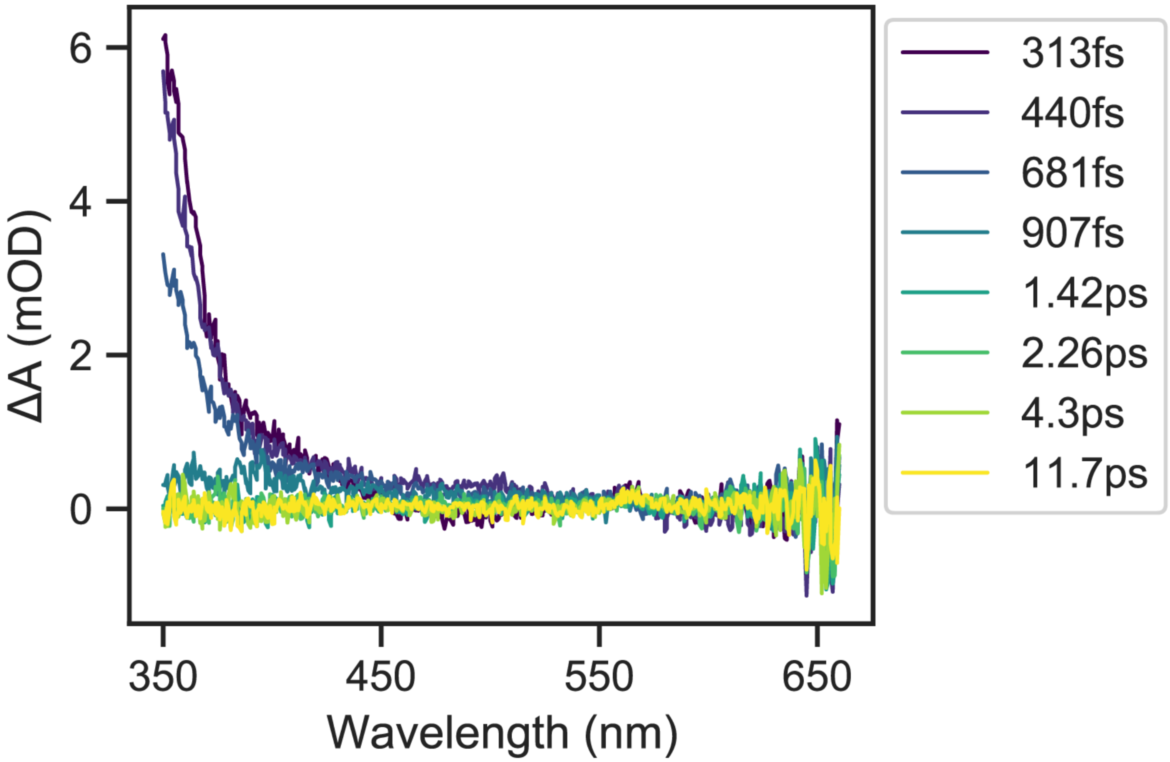

Figure S73. Methylene Chloride TA Representative spectra

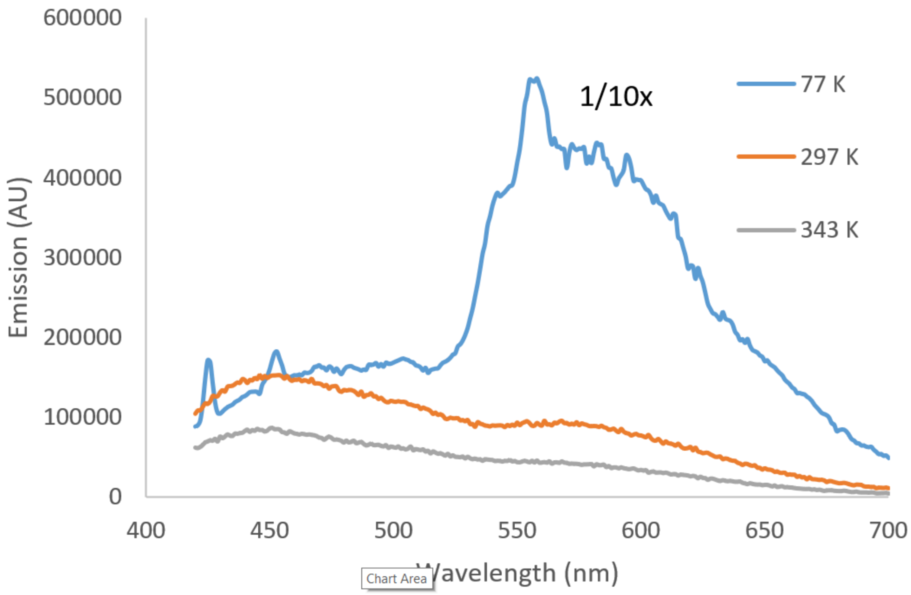

Figure S74. Compound **1A** emission at different temperatures with an excitation at 370 nm and at 297 K, 343 C, and 77 K. Note: The spectrum at 77 K has been scaled down to 1/10 the actual intensity to compare it to the other spectra.

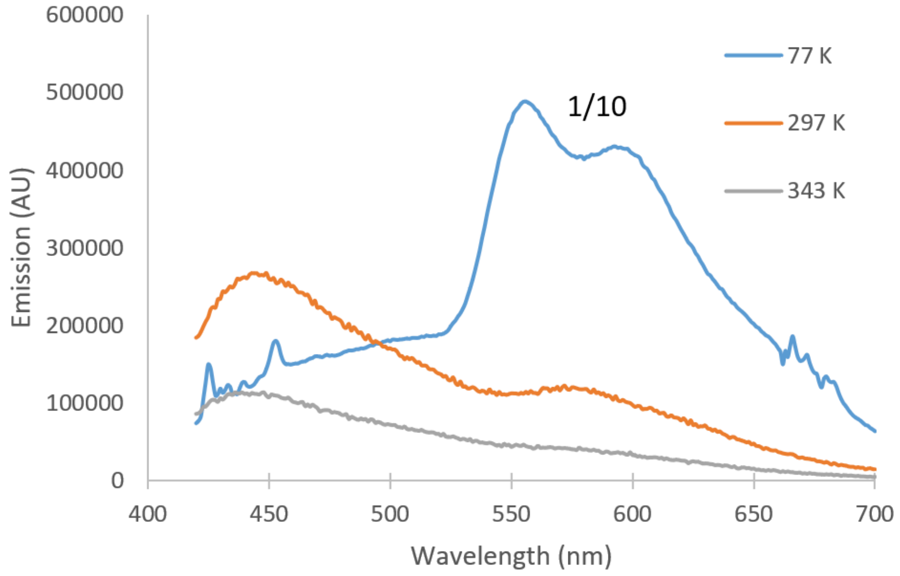

Figure S75 Compound **1D** emission at different temperatures with an excitation at 370 nm and at 297 K, 343 K, and 77 K. Note: The spectrum at 77 K has been scaled down to 1/10 the actual intensity to compare it to the other spectra.

Figure S76: Frontier and near-frontier orbitals of **1A-2D** (one compound per page; pp. 54-60)

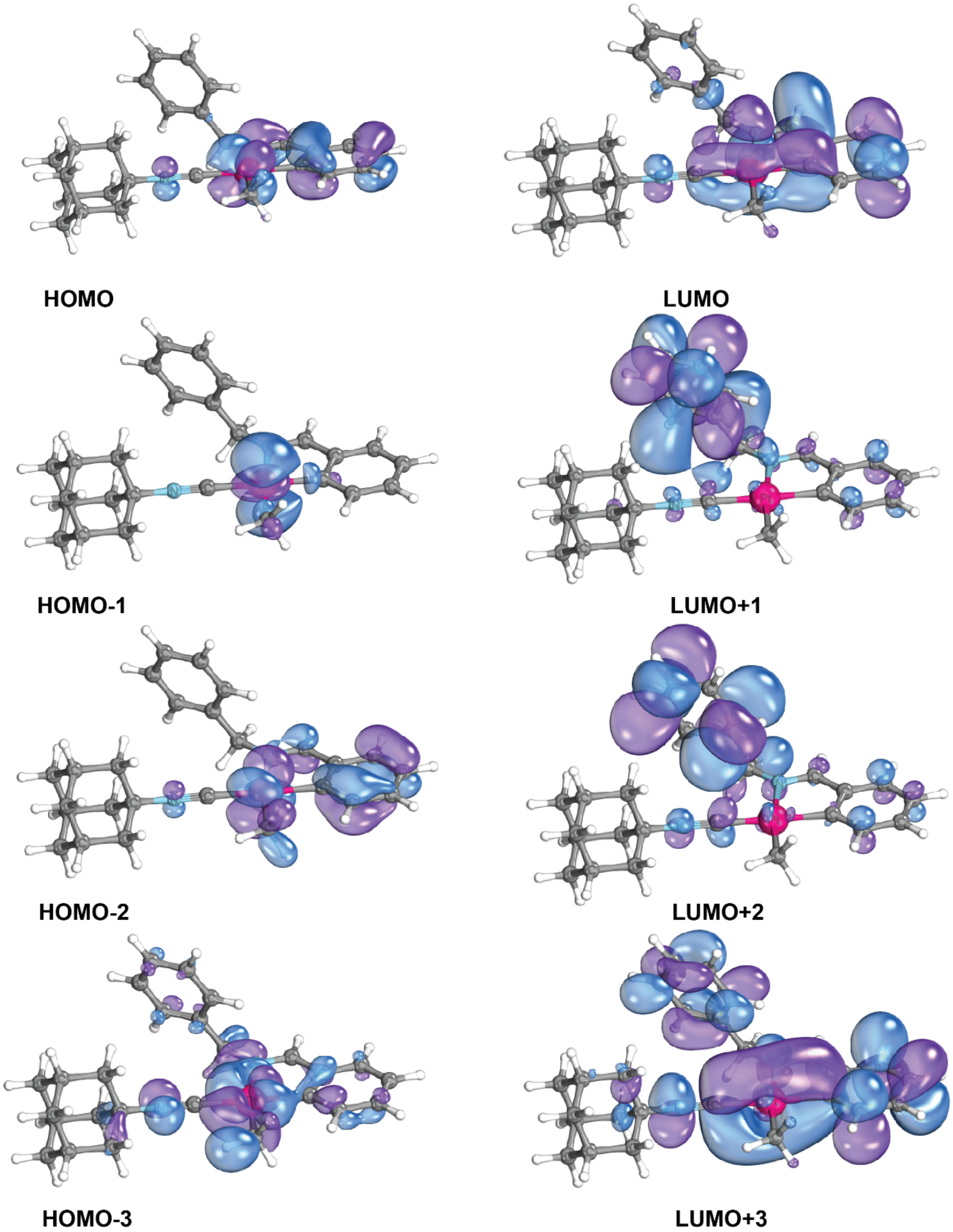

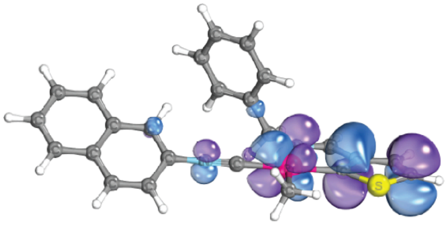

HOMO

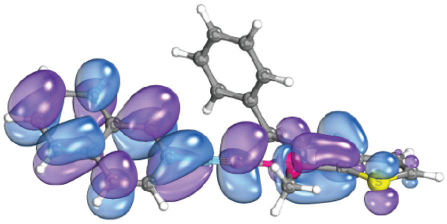

LUMO

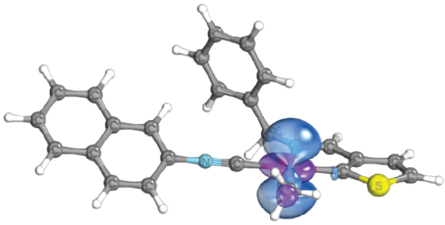

HOMO-1

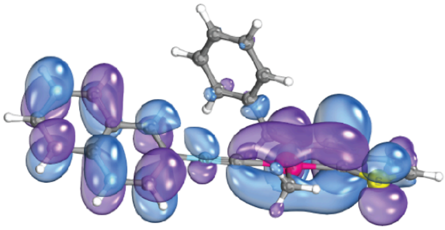

LUMO+1

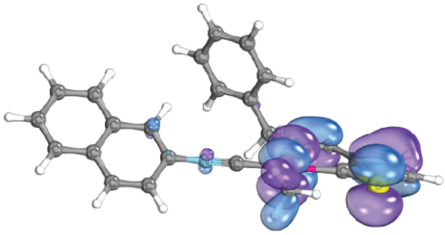

HOMO-2

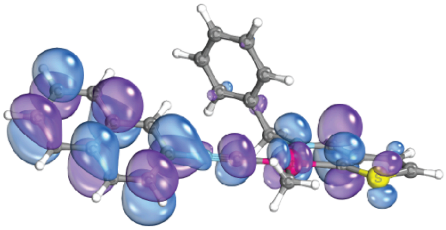

LUMO+2

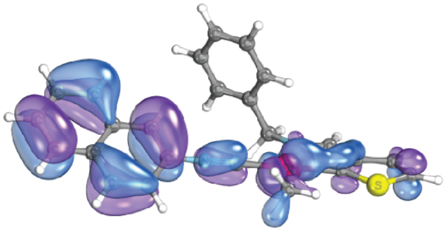

HOMO-3

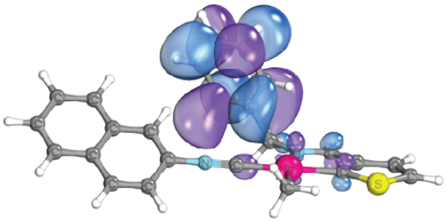

LUMO+3

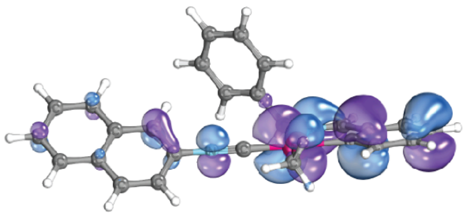

HOMO

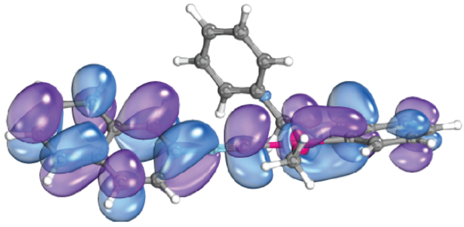

LUMO

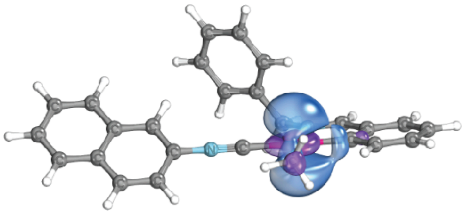

HOMO-1

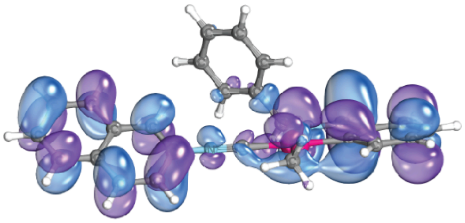

LUMO+1

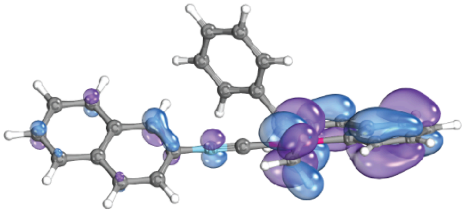

HOMO-2

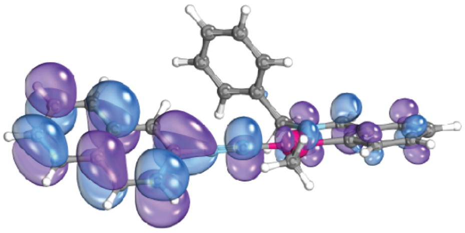

LUMO+2

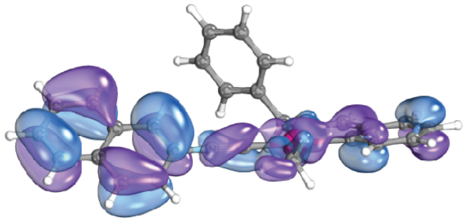

HOMO-3

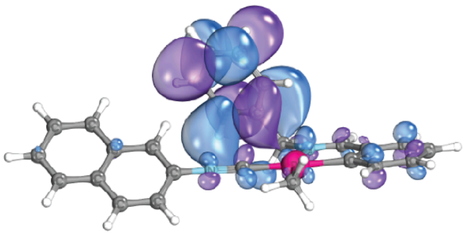

LUMO+3

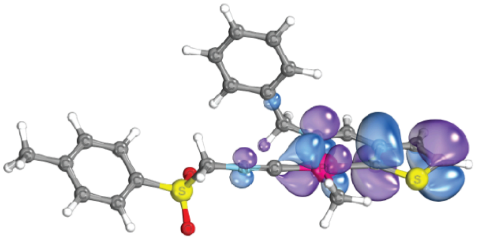

HOMO

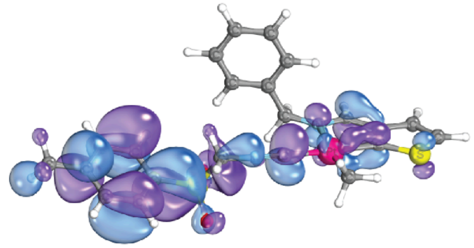

LUMO

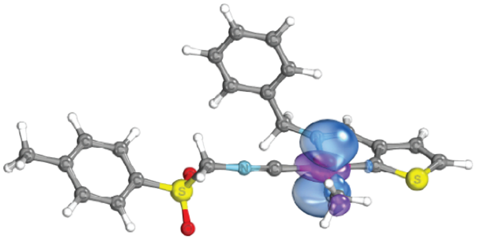

HOMO-1

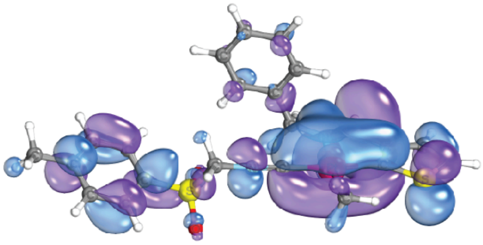

LUMO+1

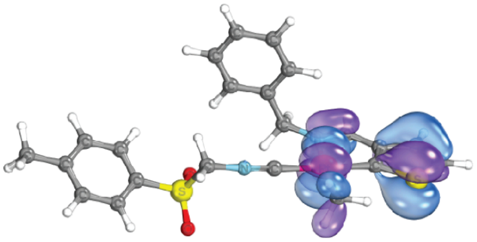

HOMO-2

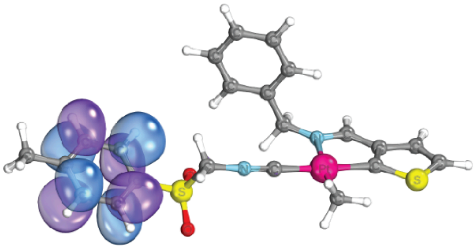

LUMO+2

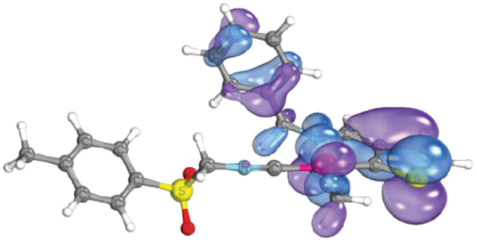

HOMO-3

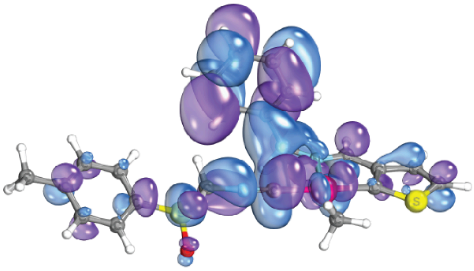

LUMO+3

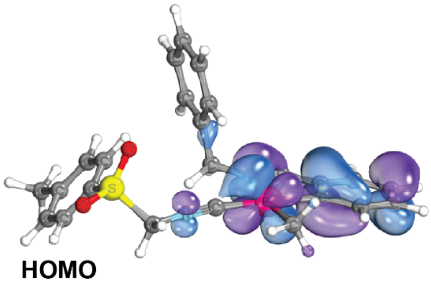

HOMO

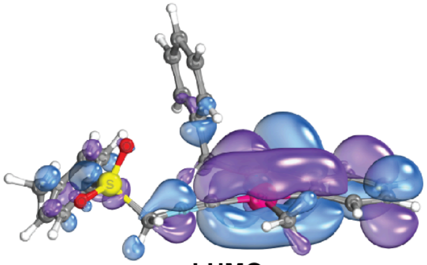

LUMO

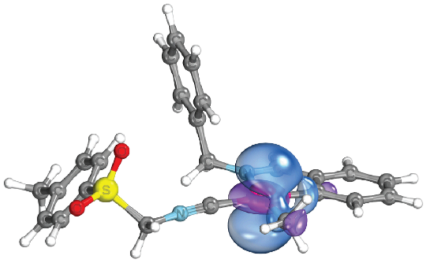

HOMO-1

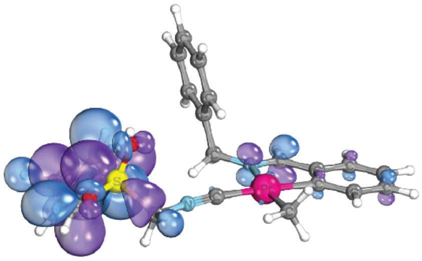

LUMO+1

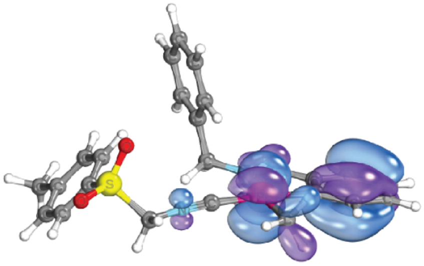

HOMO-2

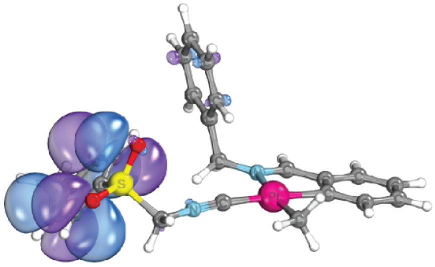

LUMO+2

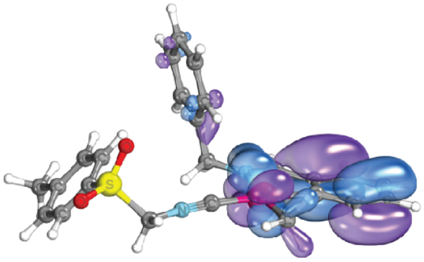

HOMO-3

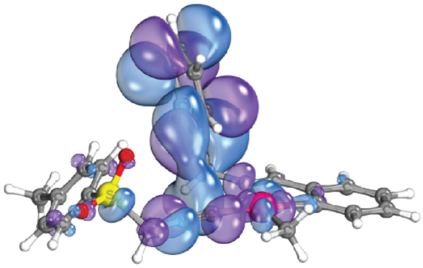

LUMO+3

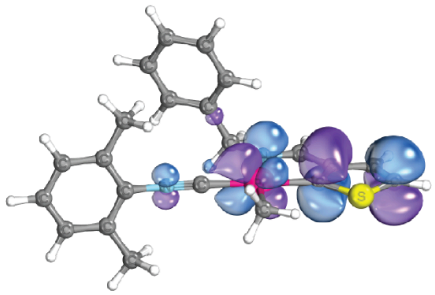

HOMO

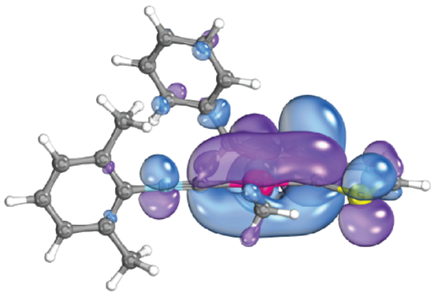

LUMO

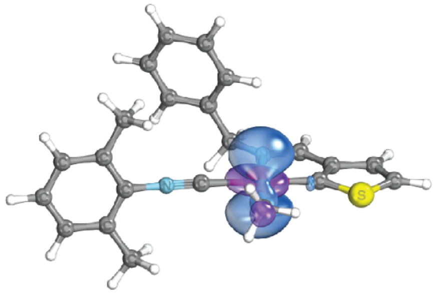

HOMO-1

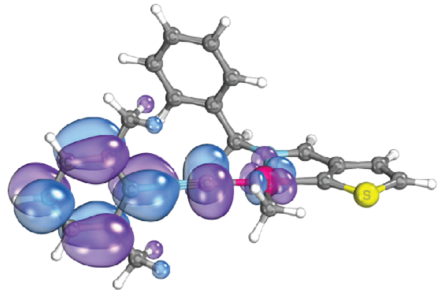

LUMO+1

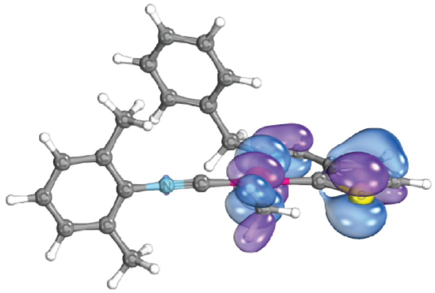

HOMO-2

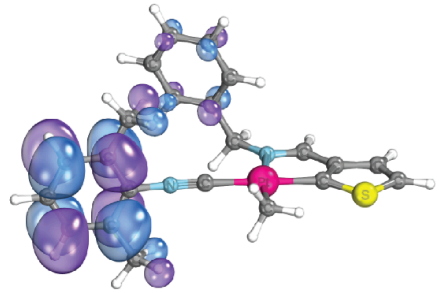

LUMO+2

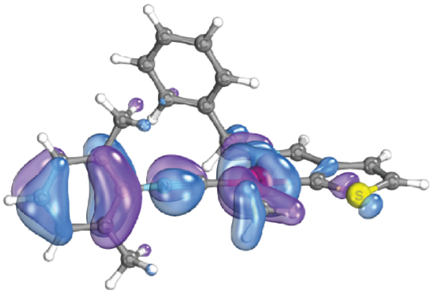

HOMO-3

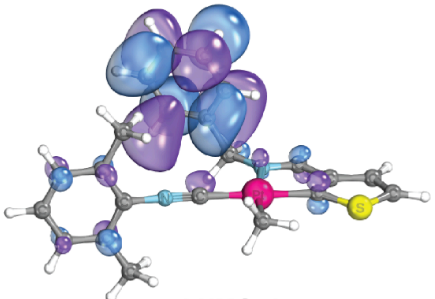

LUMO+3

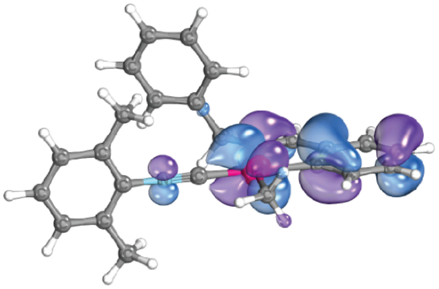

HOMO

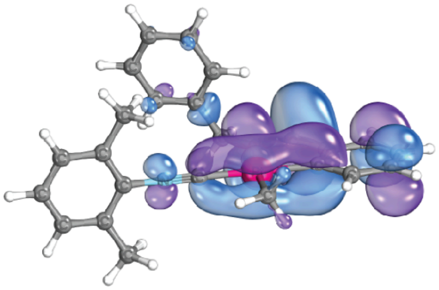

LUMO

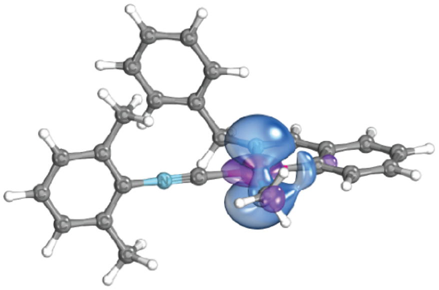

HOMO-1

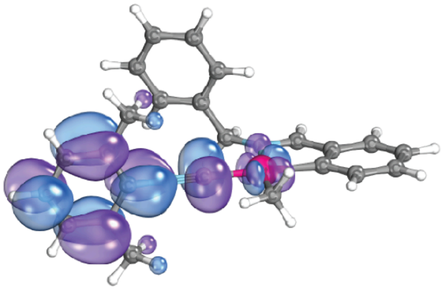

LUMO+1

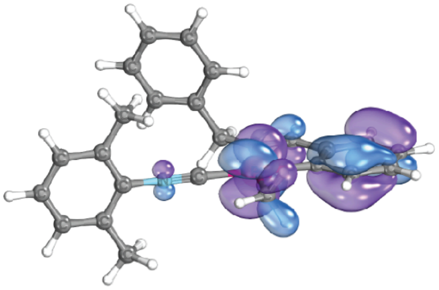

HOMO-2

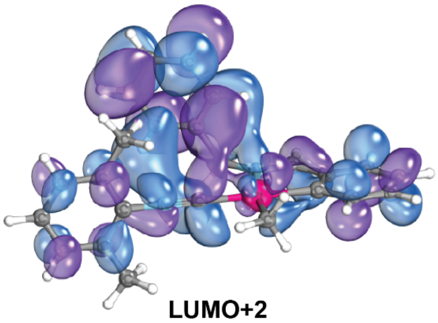

LUMO+2

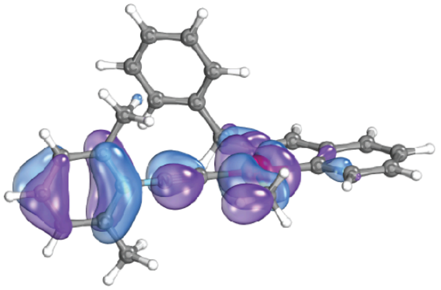

HOMO-3

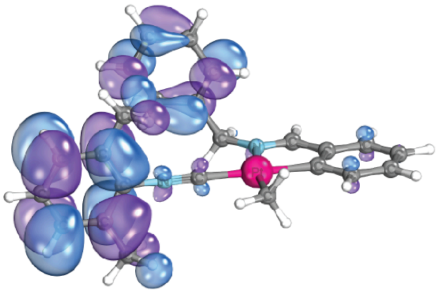

LUMO+3

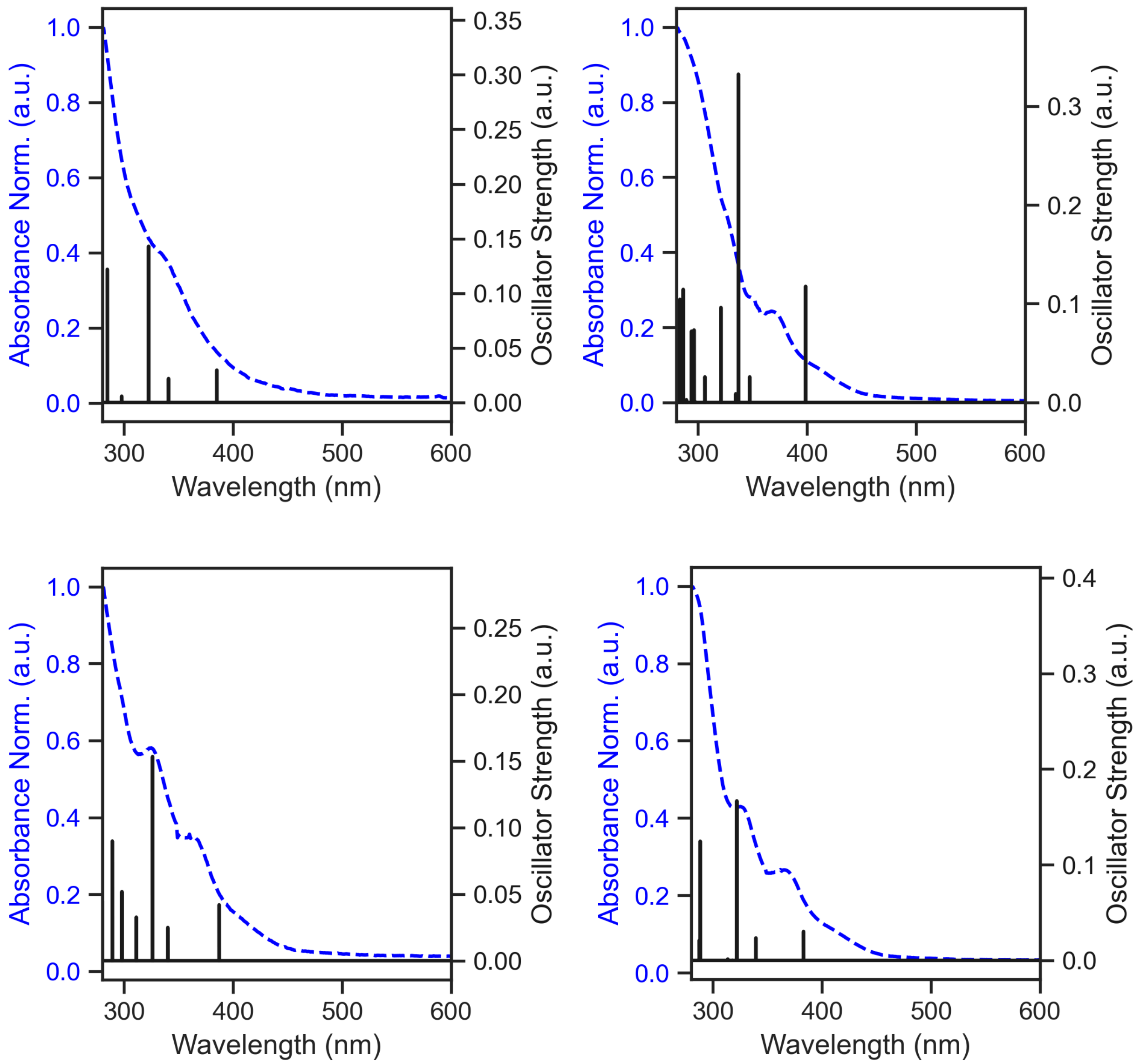

Figure S77 1A-1B (Top) and **1C-1D** (Bottom) normalized absorbance and TD-DFT calculated singlet excitation oscillator strengths from 280 nm to 600 nm. 1C is shown in Figure 11.

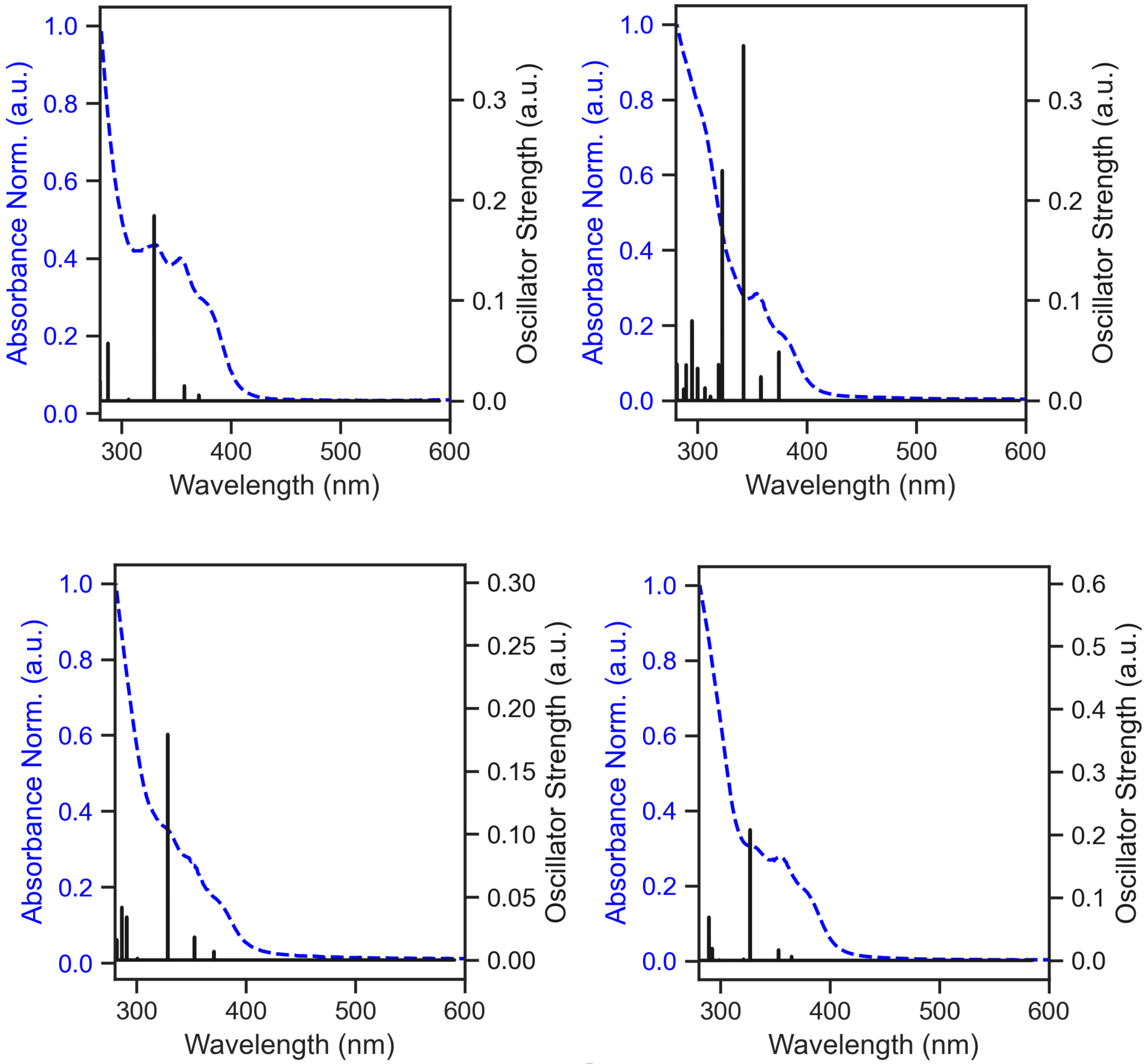

]

Figure S78 2A-2B (Top) and **2C-2D** (Bottom) Normalized absorbance and TD-DFT calculated singlet excitation oscillator strengths from 260 nm to 600 nm

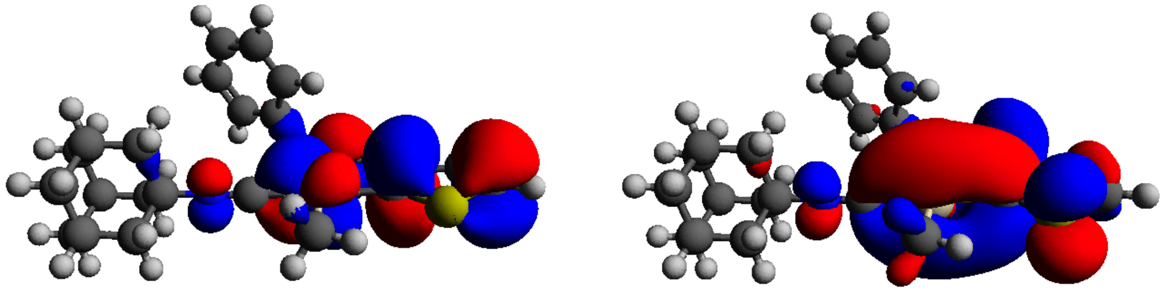

Figure S79. **1A** State 1 (3.219 eV) Major Contributing Hole (left) and Electron (right) Natural Transition Orbitals (n=0.99). Largest MO excitation weight: HOMO → LUMO (n=0.95).

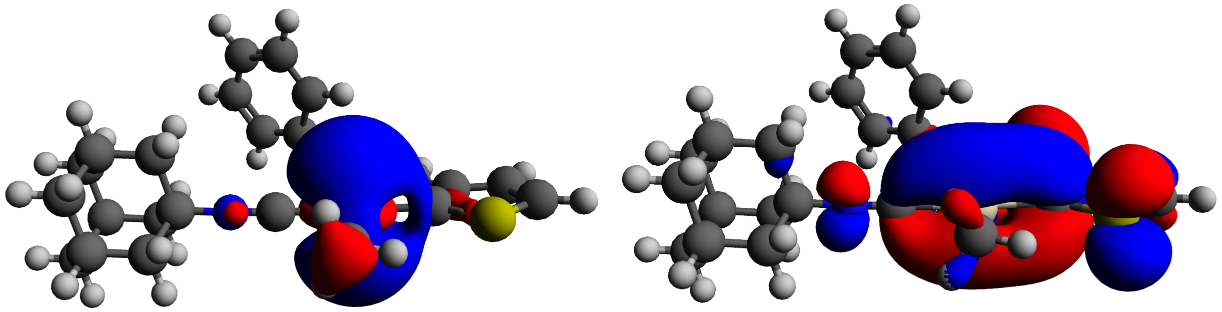

Figure S80. **1A** State 2 (3.638 eV) Major Contributing Hole (left) and Electron (right) Natural Transition Orbitals (n=0.99). Largest MO excitation weight: HOMO-1 → LUMO (n=0.97).

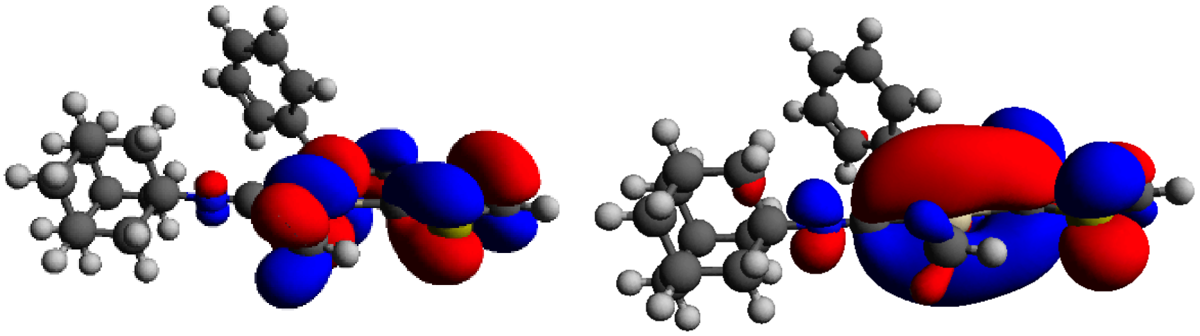

Figure S81 1A State 3 (3.844 eV) Major Contributing Hole (left) and Electron (right) Natural Transition Orbitals (n=0.97). Largest MO excitation weight: HOMO-2 → LUMO (n=0.89).

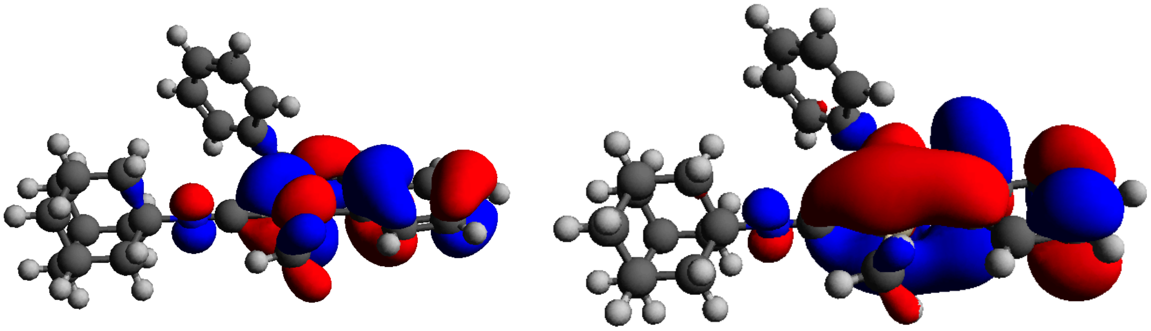

Figure S82. **2A** State 1 (3.346 eV) Major Contributing Hole (left) and Electron (right) Natural Transition Orbitals (0.99). Largest MO excitation weight: HOMO → LUMO (n=0.96).

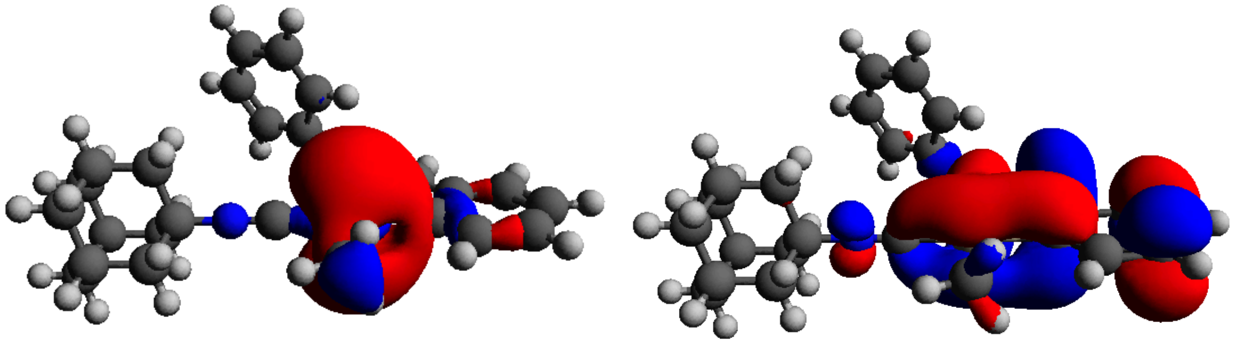

Figure S83. **2A** State 2 (3.470 eV) Major Contributing Hole (left) and Electron (right) Natural Transition Orbitals (n=0.99). Largest MO excitation weight: HOMO-1 → LUMO (n=0.99).

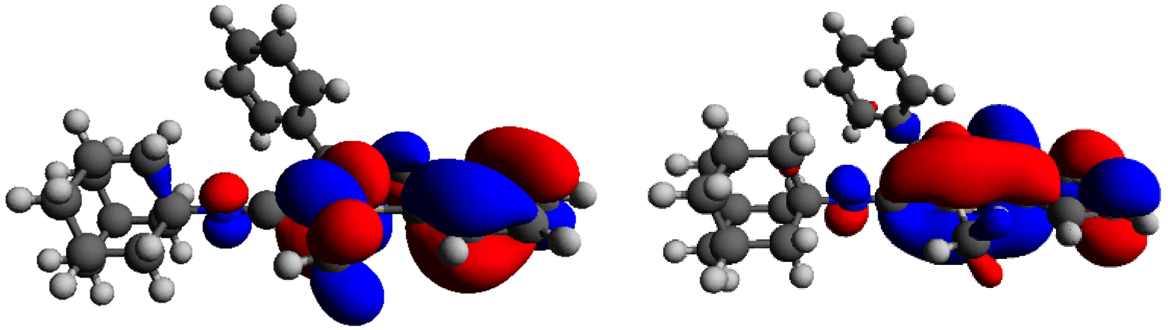

Figure S84. **2A** State 3 (3.761 eV) Major Contributing Hole (left) and Electron (right) Natural Transition Orbitals (n=0.97). Largest MO excitation weight HOMO-2 → LUMO (n=0.94).

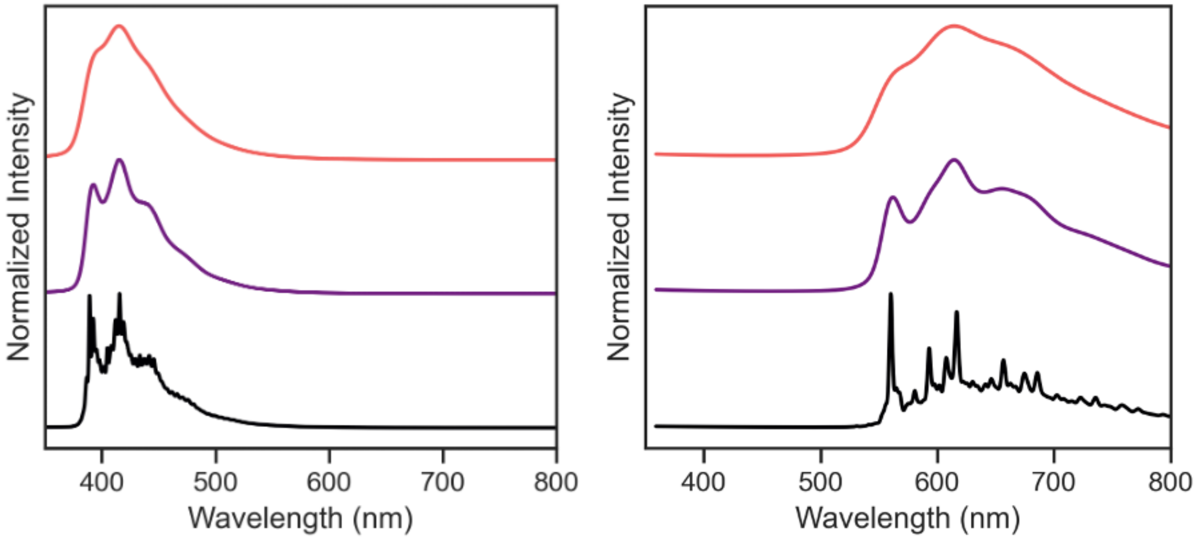

Figure S85. Normalized Franck-Condon intensities for Excited State Dynamics (ESD, Fluor) and ESD (Phosph) calculations of **1A** at the TD-DFT level. Varied Voigt linewidths are used for visualization with input flags INLINEW = 400, 200, 25 cm^{-1} and LINEW=150, 75, 10 cm^{-1} used for producing the output simulated spectra in each figure from bottom to top.

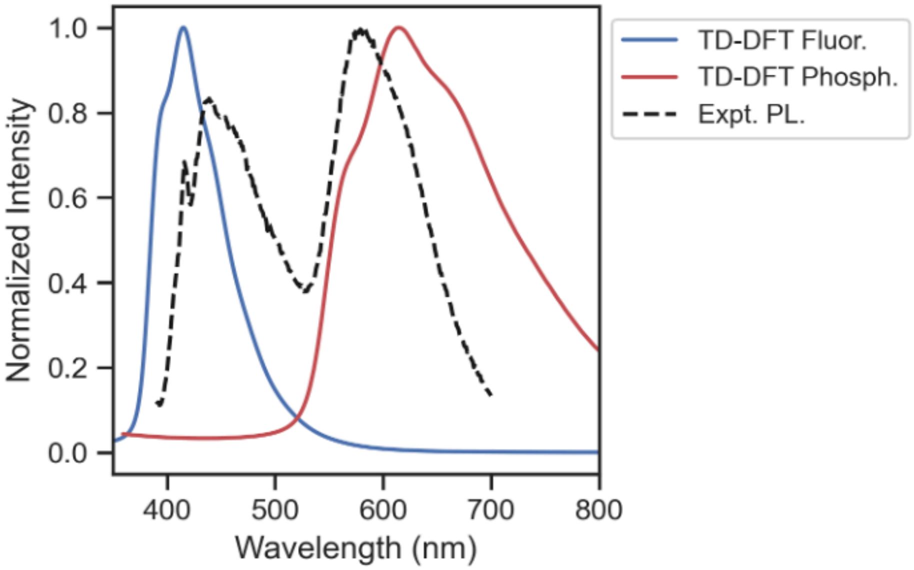

Figure S86. Experimental dual emission photoluminescence for **1A** plotted alongside the calculated normalized ESD fluorescence and phosphorescence spectra at the TD-DFT level.

DFT $\tilde{v}$(C≡N) (cm^{-1})	1A	1B	1C	1D	2A	2B	2C	2D
Complex	2225.9	2202.4	2209.2	2199.5	2219.2	2195.6	2199.9	2191.2
Free Ligand	2210.3	2197.1	2218.77	2190.4	-	-	-	-

Table S5. DFT calculated frequencies for the CN stretch relative to the free ligand.

DFT Bond Lengths (Å)	Pt-CH_3	Pt-Csp^2	Pt-N	Pt-C≡N
1A	2.069	2.033	2.198	1.946
2A	2.069	2.061	2.169	1.950
1B	2.070	2.036	2.200	1.932
2B	2.070	2.064	2.170	1.935
1C	2.070	2.040	2.195	1.922
2C	2.073	2.067	2.172	1.932
1D	2.070	2.037	2.198	1.934
2D	2.070	2.064	2.169	1.938

Table S6. DFT Calculated bond lengths.

|E| (Eh)	1A	1B	1C	1D	2A	2B	2C	2D
S_0	20480.0767	20475.2761	20950.7119	20400.2305	20157.6306	20152.8302	20628.2644	20077.7848
S_1	20479.9719	20475.1768	20950.6198	20400.1284	20157.5321	20152.7317	20628.1675	20077.6858
T_1	20479.9965	20475.1826	20950.6308	20400.1504	20157.5465	20152.7453	20628.1817	20077.6998
T_1-S_0	0.0802	0.0935	0.0810	0.0801	0.0842	0.0849	0.0827	0.0850
S_1-S_0	0.1048	0.0993	0.0921	0.1021	0.0986	0.0985	0.0969	0.0990
S_1-T_1	0.0246	0.0058	0.0110	0.0220	0.0144	0.0136	0.0142	0.0140

Table S7. Calculated energies and energy differences between S1-S0, and T1-S0 states.

ΔE photon λ (nm)	1A	1B	1C	1D	2A	2B	2C	2D
T_1-S_0	568.2	487.5	562.3	569.0	541.5	536.8	551.1	536.2
S_1-S_0	434.6	459.0	494.9	446.4	462.3	462.8	470.3	460.5

Table S8. Calculated wavelengths corresponding to the emission between excited and ground states.

References:

1) Suzuki, K. PhysChemChemPhys 2009, 11, 9850

2) Horiba Scientific Application Note: "PLQY in NIR with QuantaMaster and K-sphere"

3)Van Houten, J.; Watts, R. J. Temperature Dependence of the Photophysical and Photochemical Properties of the Tris(2,2'-Bipyridyl)Ruthenium(II) Ion in Aqueous Solution. *J. Am. Chem. Soc.* 1976, *98* (16), 4853– 4858,

4)Nakamaru, K. Synthesis, Luminescence Quantum Yields, and Lifetimes of Trischelated Ruthenium(II) Mixed-Ligand Complexes Including 3,3′-Dimethyl-2,2′-Bipyridyl. *Bull. Chem. Soc. Jpn.* 1982, *55* (9), 2697– 2705,

5) Lakowicz, J. R. *Principles of Fluorescence Spectroscopy*, 3rd ed.; Lakowicz, J. R., Ed.; Springer US: USA, 2006
